# Global ubiquitylation analysis of mitochondria in primary neurons identifies endogenous Parkin targets following activation of PINK1

**DOI:** 10.1126/sciadv.abj0722

**Published:** 2021-11-12

**Authors:** Odetta Antico, Alban Ordureau, Michael Stevens, Francois Singh, Raja S. Nirujogi, Marek Gierlinski, Erica Barini, Mollie L. Rickwood, Alan Prescott, Rachel Toth, Ian G. Ganley, J. Wade Harper, Miratul M. K. Muqit

**Affiliations:** 1 MRC Protein Phosphorylation and Ubiquitylation Unit, School of Life Sciences, University of Dundee, Dundee DD1 5EH, UK.; 2 Department of Cell Biology, Harvard Medical School, Boston, MA 02115, USA.; 3 Aligning Science Across Parkinson’s (ASAP) Collaborative Research Network, Chevy Chase, MD 20815, USA.; 4 Data Analysis Group, Division of Computational Biology, School of Life Sciences, University of Dundee, Dundee DD1 5EH, UK.; 5 Dundee Imaging Facility, School of Life Sciences, University of Dundee, Dundee DD1 5EH, UK.

## Abstract

How activation of PINK1 and Parkin leads to elimination of damaged mitochondria by mitophagy is largely based on cell lines with few studies in neurons. Here, we have undertaken proteomic analysis of mitochondria from mouse neurons to identify ubiquitylated substrates of endogenous Parkin. Comparative analysis with human iNeuron datasets revealed a subset of 49 PINK1 activation–dependent diGLY sites in 22 proteins conserved across mouse and human systems. We use reconstitution assays to demonstrate direct ubiquitylation by Parkin in vitro. We also identified a subset of cytoplasmic proteins recruited to mitochondria that undergo PINK1 and Parkin independent ubiquitylation, indicating the presence of alternate ubiquitin E3 ligase pathways that are activated by mitochondrial depolarization in neurons. Last, we have developed an online resource to search for ubiquitin sites and enzymes in mitochondria of neurons, MitoNUb. These findings will aid future studies to understand Parkin activation in neuronal subtypes.

## Teaser

Identification of neuronal targets of PD-linked enzyme Parkin by quantitative ubiquitin-based proteomics.

## INTRODUCTION

Mitochondria perform diverse functions within eukaryotic cells that are essential to their survival; however, under physiological conditions, they are exposed to pleiotropic stress including reactive oxygen species and misfolded and aggregated proteins that cause mitochondrial dysfunction (*1*, *2*). This is particularly relevant in terminally differentiated cell types such as neurons in which aberrant mitochondrial homeostasis is linked to diverse neurological syndromes and neurodegenerative disorders (*3*, *4*). Mitochondrial quality control pathways have evolved to sense and protect against mitotoxic stress, of which elimination to lysosomes via the autophagy pathway (mitophagy) has been extensively studied in recent years (*2*, *5*). Autosomal recessive mutations in human phosphatase and tensin homolog (PTEN)–induced kinase 1 (PINK1) and the RING-In-Between-Ring (IBR)-RING ubiquitin E3 ligase Parkin (encoded by *PARK6* and *PARK2* genes, respectively) are causal for early-onset Parkinson’s disease (PD) (*6*, *7*). Landmark cell-based studies have demonstrated that these proteins function within a common pathway to regulate stress-evoked mitophagy via a ubiquitin-dependent mechanism (*8*–*10*). Upon loss of mitochondrial membrane potential that can be induced artificially by mitochondrial uncouplers [e.g., antimycin A/oligomycin (AO)], PINK1 becomes stabilized and activated on the mitochondrial outer membrane (MOM) where it phosphorylates both ubiquitin and Parkin at their respective Ser^65^ residues, leading to stepwise recruitment and activation of Parkin at the MOM (*11*–*15*). An analogous pathway is activated upon accumulation of misfolded proteins in the mitochondrial matrix or upon mitochondrial damage occurring in cells lacking mitofusin proteins (MFN1 and MFN2), indicative of physiologically relevant mitochondrial stress pathways (*16*, *17*). Active Parkin ubiquitylates myriad substrates including voltage-dependent anion channels (VDACs), MitoNEET/CDGSH iron-sulfur domain-containing protein 1 (CISD1), MFNs, hexokinase 1 (HK1), and mitochondrial Rho GTPases (guanosine triphosphatases) (MIROs)/RHOTs, leading to its further recruitment and retention at the MOM via a Ser^65^–phosphorylated ubiquitin (phospho-ubiquitin)–dependent feed-forward amplification mechanism (*15*, *18*–*22*). The presence of ubiquitin and/or phospho-ubiquitin on damaged mitochondria stimulates recruitment of autophagy adaptor receptors of which nuclear domain 10 protein 52 (NDP52), optineurin (OPTN), and, to a small extent, Tax1bp1 are required for efficient clearance of damaged mitochondria (*19*, *23*, *24*).

The initial mechanisms by which Parkin is activated and identification of bona fide substrates have been mainly studied in human cancer lines overexpressing Parkin, and to date, very few studies have assessed its molecular function in physiologically relevant neuron cell types at the endogenous level. Previous analysis of ubiquitylation in HeLa cells overexpressing Parkin found highest levels of ubiquitylation in the abundant VDAC1/2/3 proteins and also revealed moderate ubiquitylation of specific sites within MFN2, CISD1, and mitochondrial import receptor subunit TOM20 homolog (TOMM20) proteins (*19*, *20*, *25*). Ubiquitylation analysis in human embryonic stem cell–derived iNeurons (expressing markers of excitatory cortical neurons) has revealed endogenous PINK1–dependent accumulation of phospho-ubiquitin: a 70-, 14-, and 2-fold increase in K63, K11, and K6 ubiquitin linkages (the latter being lower than that observed in HeLa cells overexpressing Parkin) and 134 ubiquitylation sites spanning 83 proteins of which the majority are localized at the MOM including VDAC1/3 and MFN2 (*18*, *19*). Collectively these studies have elaborated a model that suggests that PINK1- and Parkin-dependent accumulation of ubiquitin chain types mainly at the MOM is sufficient for downstream signaling and mitochondrial clearance (*18*, *26*).

In previous studies, we have reported that endogenous PINK1 and Parkin activation can be robustly measured in primary cortical neurons derived from mouse embryonic-derived neuronal progenitors, that is, an established and widely validated cell system to investigate neurobiological mechanisms relevant to mature neurons (*27*–*29*). Here, we have undertaken global di-Glycine (diGLY) ubiquitylation analysis in primary cortical neurons of wild-type and homozygous PINK1 knockout (KO) mice to map the ubiquitin architecture of mitochondria under normal basal conditions and upon activation of PINK1 upon mitochondrial depolarization. After 5 hours of stimulation, we detect ubiquitylation of 58 mitochondrial proteins located mainly within the MOM, associated with an increase in K63 ubiquitin chain linkage type, and a low level of mitochondrial protein turnover at this time point that is blocked in PINK1 KO neurons. We further demonstrate the regulation of mitochondrial substrates by Parkin using cell-based assays in Parkin KO neurons and Parkin reconstitution in vitro catalytic activity assays. Comparative analysis with human iNeurons datasets elaborated a PINK1/Parkin-dependent ubiquitylation signature on depolarized mitochondria in neurons comprising 49 sites across 22 MOM proteins conserved between mouse and human neurons that represent a cellular readout for PINK1 and Parkin activity and a potential proxy readout of PD-linked mitochondrial dysfunction in future studies.

## RESULTS

### Generation and characterization of wild-type and PINK1 KO mouse neurons

**Fig. 1.**
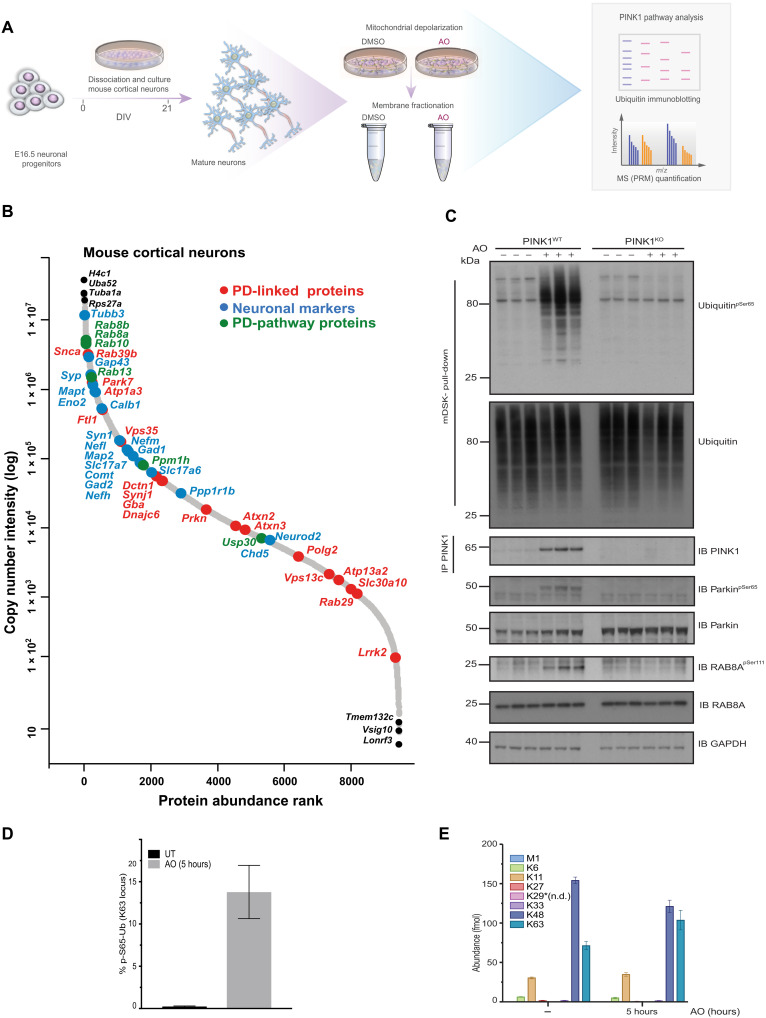
PINK1 signaling in mouse cortical neurons. (**A**) Experimental workflow in primary mouse neurons. E16.5 cortical neurons were cultured for 21 DIV, and membrane enrichment was performed after mitochondrial depolarization induced with 10 μM antimycin A combined with 1 μM oligomycin for 5 hours. DMSO, dimethyl sulfoxide; *m*/*z*, mass/charge ratio. (**B**) Copy number: Rank abundance plot depicting the protein copy number abundance of cortical neurons. The *x* axis denotes copy number abundance rank, and the *y* axis denotes log-transformed copy number intensity. PD-linked genes, neuronal markers, and PINK1-Parkin pathway components are highlighted in red, blue, and green colored circles and text, respectively. (**C**) Immunoblots showing comparative analysis of phospho-Ser^65^ ubiquitin levels in primary cortical neuron cultures from wild-type (WT) and PINK1 KO mice. Cultures were stimulated with antimycin A and oligomycin for 5 hours before membrane enrichment. Phospho-Ser^65^ ubiquitin was detected by immunoblotting after ubiquitin enrichment by incubating with ubiquitin-binding resin derived from Halo-multiDSK (mDSK). Affinity captured lysates were also subjected to immunoblotting with total ubiquitin antibody. Immunoprecipitation (IP) showed PINK1 protein stabilization after mitochondrial depolarization. Phospho-Ser^111^ Rab8A and phospho-Ser^65^ Parkin were detected by immunoblotting. GAPDH, glyceraldehyde-3-phosphate dehydrogenase. Lysates were also subjected to immunoblotting with indicated antibodies for loading and protein expression controls. IB, immunoblot. (**D** and **E**) C57BL/6J mouse cortical neurons (DIV 21) were depolarized with AO for 5 hours, and whole-cell lysates were subjected to Pt-PRM (parallel reaction monitoring) quantification. Abundance (fmol) for individual ubiquitin chain linkage types. Untreated (UT) (D) and percentage of phospho-Ser^65^ ubiquitin (C) is plotted. Error bars represent SEMs (*n* = 3). n.d., not determined.

To characterize PINK1 and Parkin activity in neurons, we established primary cortical neuron cultures from embryonic day 16.5 (E16.5) mouse embryos at 21 days in vitro (DIV) (Fig. 1A). Immunofluorescence analysis of wild-type mouse DIV21 cultures using anti-microtubule associated protein 2 (MAP2) (neuronal marker) and anti–glial fibrillary acidic protein (GFAP; glial marker) antibodies confirmed that cultures were highly enriched for neuronal cells (~85%) (fig. S1A). We next undertook deep proteomic profiling of wild-type cortical neurons using the histone-based proteomic ruler method to estimate the copy number of neuronal markers and PD-linked proteins (*30*). This revealed high copy number of multiple neuronal markers including Tubb3 (~11.6 million copies per cell), Syp (~1.6 million copies per cell), and Mapt and Eno2 (~1 million and 1.1 million copies per cell, respectively) (Fig. 1B). Parkin has lower abundance (~18 thousand copies per cell) and ranked at 3672 on our list of 9431 most abundant proteins, whereas we were unable to detect PINK1 peptides under the conditions used (Fig. 1B). Of the other PD-linked proteins, from highest to lowest abundance, included SNCA (α-synuclein) (~4.7 million copies per cell), Parkinson disease protein 7 homolog (PARK7/DJ1) (~1.5 million copies per cell), Vacuolar protein sorting-associated protein 35 (VPS35) (~175 thousand copies per cell), VPS13C and polyamine-transporting ATPase 13A2 (ATP13A2) (both 2 thousand copies per cell), and Leucine-rich repeat serine/threonine-protein kinase 2 (LRRK2) (~133 copies per cell). Other PINK1 pathway components included Ras-related protein Rab-8B (Rab8B) (~4.5 million copies per cell), Ras-related protein Rab-8A (Rab8A) (~4.5 million copies per cell), Ras-related protein Rab-13 (Rab13) (~1.4 million copies per cell), and Ubiquitin carboxyl-terminal hydrolase 30 (USP30) (~7 thousand copies per cell) (Fig. 1B and table S1). We also generated quantitative global proteomic analysis of cortical neurons that are treated with antimycin A (10 μM)/oligomycin (1 μM) stimulation for 5 hours [inhibition of respiratory chain enzyme complex II/III and adenosine triphosphate (ATP) synthase respectively; AO]. We undertook data-independent acquisition (DIA) that led to identification and quantification of 6300 protein groups. We did not detect a substantial portion of the proteome being altered by AO at this time point with only three proteins significantly increased (Fam216a, Mtm1, and Smarcd2) and several decreased including Smyd4, Med29, Ccdc106, and Nktr (fig. S1B and table S1). We next undertook high-resolution respirometry of DIV21 cultures of wild-type and PINK1 KO neurons under the influence of different substrates and inhibitors (fig. S2, A and B). Overall, we did not observe any major difference between wild-type and PINK1 KO neurons in their utilization of substrates (fig. S2B). There was a mild global impairment of mitochondrial respiration in PINK1 KO primary compared to wild-type neurons [repeated measure analysis of variance (ANOVA), *P* ≤ 0.05]. In addition, we observed a mild decrease in the phosphorylation system control ratio (P/E control ratio) in PINK1 KO neurons (fig. S2C), suggesting an enhanced coupling of oxidation and phosphorylation in PINK1 KO primary neurons that may compensate for the reduction in mitochondrial respiration and maintain physiological ATP levels.

### Biochemical characterization of PINK1-induced ubiquitin signaling in mouse neurons

We undertook a time course analysis of PINK1 activation in primary mouse neurons following AO stimulation. Endogenous PINK1 levels were detectable following immunoprecipitation-immunoblot analysis, revealing very low expression in neurons under basal conditions and increased expression over time upon mitochondrial depolarization (fig. S3A). Immunoblot analysis of Parkin demonstrated basal expression that was stable upon mitochondrial depolarization up to 9 hours (fig. S3A). We optimized the detection of phospho-ubiquitin using HALO-multiDSK and immunoblotting with anti–phospho-Ser^65^ ubiquitin antibody and signal was abolished using a mutant form of HALO-multiDSK (fig. S3B). Furthermore, we did not observe any difference in the detection of phospho-ubiquitin using tandem-repeated ubiquitin-binding entities (TUBE) pulldowns with HALO-multiDSK and HALO-UBA^UBQLN1^ (fig. S3B). Upon mitochondrial depolarization, we observed robust accumulation of phospho-ubiquitin at 3 hours, and this was maximal at 5 hours; similarly, we also detected phospho-Parkin upon mitochondrial depolarization (fig. S3A). We therefore undertook all subsequent analysis at this time point. We next investigated signaling in PINK1 KO neurons and observed complete loss of phospho-ubiquitin (Fig. 1C). We have previously found that PINK1 activation leads to induction of phosphorylation of a subset of Rab GTPases including Rab8A at Ser^111^ in human cancer cell lines (*31*), and upon mitochondrial depolarization, we observed Ser^111^-phosphorylated Rab8A (phospho-Rab8A) in wild-type neurons, and this was abolished in PINK1 KO neurons (Fig. 1C). Upon mitochondrial depolarization of wild-type and Parkin KO neurons, we observed loss of phospho-Parkin in Parkin KO neurons and, while the level of phospho-ubiquitin was substantially reduced, it was not abolished, suggesting that other unknown ubiquitin E3 ligases may contribute ubiquitin marks that can be phosphorylated by PINK1 in mature neurons (fig. S3C), a finding consistent with studies in Parkin-deficient iNeurons (*19*). Furthermore, we observed decreased PINK1 levels in Parkin KO neurons, consistent with previous observations in human Parkin S65A iNeurons (*19*) and human Parkin S65N patient–derived fibroblasts (*29*), suggesting a potential feedback mechanism of Parkin activation on PINK1 stabilization. We have previously observed that PINK1-induced phospho-Rab8A can occur in HeLa cells that lack Parkin or in Parkin KO mouse embryonic fibroblasts (MEFs), and consistent with this, we did not observe any difference in phospho-Rab8A in wild-type or Parkin KO neurons following mitochondrial depolarization (fig. S3C). It has been reported that additional monogenic forms of Parkinson’s may interplay with the PINK1/Parkin pathway including the Asp620Asn (D620N) mutation of the retromer-associated VPS35 gene (*32*, *33*). However, we did not observe any alteration in phospho-ubiquitin or phospho-Rab8A in VPS35 D620N homozygous and heterozygous neurons compared to wild-type controls following mitochondrial depolarization (fig. S4).

We next used targeted parallel reaction monitoring (PRM) proteomic analysis in whole-cell lysates of mature DIV21 neurons to quantitatively assess ubiquitin changes upon AO stimulation and observed an approximate 52-fold increase in phospho-ubiquitin with a stoichiometry of ubiquitin phosphorylation of ~0.13 at 5-hour treatment (Fig. 1D). Under basal conditions, we detected nearly all ubiquitin chain linkage types including K6, K11, K27, K33, K48, and K63, and upon 5 hours of AO treatment, we only observed an increase in K63 chain linkages (Fig. 1E). In parallel studies, we undertook PRM analysis of less mature DIV12 neurons in which Parkin expression is lower (*28*) and stimulated these cells with AO for less time at 3 hours. In TUBE pulldowns of whole-cell lysates or mitochondrial-enriched fractions, we observed an approximate 300-fold and 40-fold increase in phospho-ubiquitin, respectively, that was not associated with any significant increase in ubiquitin chain linkage types (fig. S5). This suggests that basal mitochondrial ubiquitin levels in neurons may be sufficient to promote the initial generation of phospho-ubiquitin under conditions where Parkin activity is not maximal.

### Quantitative proteome and diGLY proteome of mitochondria in mouse cortical neurons under basal conditions and mitochondrial depolarization

**Fig. 2.**
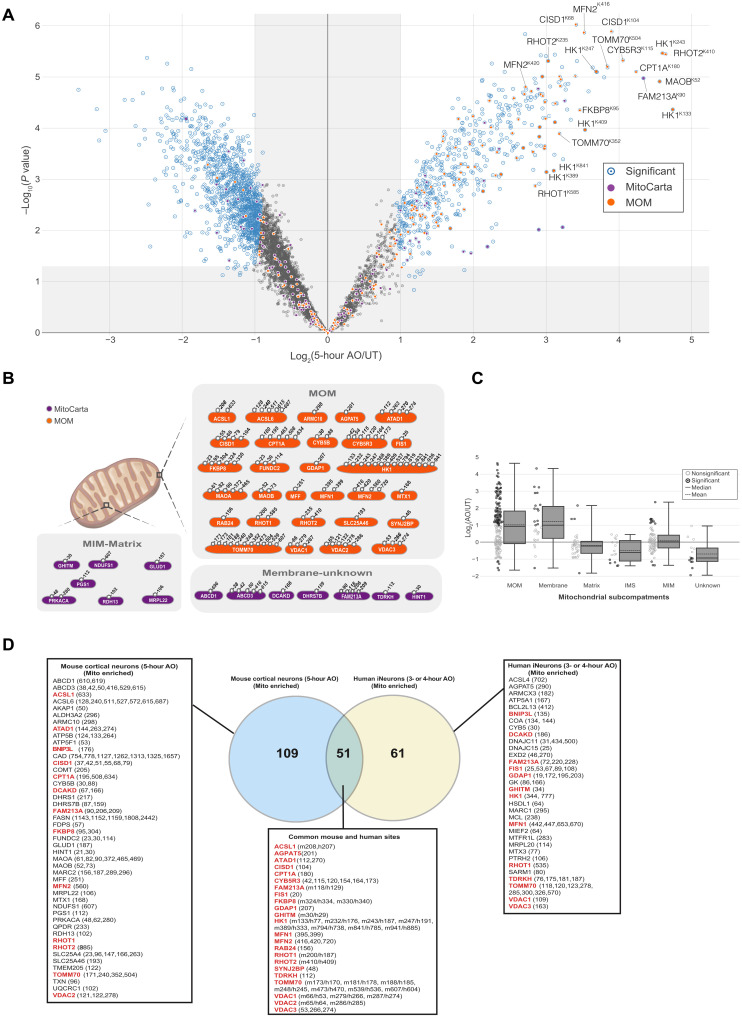
Global ubiquitylation analysis of mitochondria in neurons upon mitochondrial depolarization. (**A**) C57BL/6J primary cortical neurons were depolarized with AO (5 hours), and membrane-enriched lysates were subjected to diGLY capture proteomics. Fold increase for individual ubiquitylated targets is shown in the volcano plot. The *x* axis specifies the fold changes, and the *y* axis specifies the negative logarithm to the base 10 of the *t* test *P* values. Dots (1606) reflect the significant hits [Welch’s t test (S0 = 2), corrected for multiple comparison by permutation-based false discovery rate (FDR; 1%)]. Five hundred fifty-nine and 1047 dots represent ubiquitylated targets up-regulated or down-regulated after mitochondrial depolarization, respectively. diGLY peptide of proteins associated with mitochondria (MitoCarta 3.0) or MOM localization are indicated. (**B**) Schematic showing sites of ubiquitylation in mouse cortical neurons according to MitoCarta 3.0. Residue numbers for diGLY-modified Lys residues are shown. (**C**) Distribution of changes in diGLY peptides for proteins that localize in the mitochondrial subcompartments: matrix, MIM, MOM, IMS, membrane, and unknown (MitoCarta 3.0). (**D**) Venn diagram of overlapping diGLY sites observed in site observed for mitochondrial enriched mouse cortical neurons (5 hours after depolarization) and sites observed from mitochondrial enriched human iNeurons (3 or 4 hours after depolarization) (*18*). All peptides used were increased by at least twofold (log_2_ ratio > 1.0), with *P* < 0.05. diGLY sites or proteins common to mouse and human datasets are marked in red bold.

We next used a tandem mass tagging (TMT)–MS3–based pipeline (*25*) (Fig. 2A) to quantify the mitochondrial proteome abundance and the mitochondrial ubiquitylome under basal conditions and upon mitochondrial depolarization that has been previously deployed in human iNeurons (*18*). We isolated mitochondria-containing membrane fractions from quintuplicate cultures of DIV21 C57BL/6J mouse primary cortical neurons that were untreated or treated with 10 μM antimycin A and 1 μM oligomycin (AO) for 5 hours (fig. S6, A and B). We observed few changes in the proteome abundance following mitochondrial depolarization; of the 6255 proteins quantified (table S2), 4 proteins [Calsyntenin-2 (CLSTN2), Synaptotagmin-5 (SYT5), Myocardin-related transcription factor A (MKL1), and Histone H2B type 3-A (HIST3H2BA)] were significantly reduced and 1 protein [Protein phosphatase 1H (PPM1H)] significantly increased after AO treatment (fig. S6C). There was a slight shift to the left in the AO proteome compared to the untreated proteome, suggesting a low level of turnover (fig. S6C). This is consistent with analysis undertaken in iNeurons stimulated with AO (*18*).

We next determined the mitochondrial ubiquitylome in mouse neurons using diGLY affinity capture coupled with quantitative proteomics (Fig. 2A and fig. S6, A and B). Tryptic peptides from membrane-enriched extracts of mouse primary cortical neurons untreated or treated with AO for 5 hours were subjected to α-diGLY immunoprecipitation, and samples were analyzed using 11-plex TMT-MS^3^ with diGLY peptide intensities normalized with total protein abundance measured in parallel (Fig. 2A and table S2; see Materials and Methods). We detected and quantified ~9154 diGLY-containing Kgg peptides, of which 5616 are unique sites. Of those unique sites, 559 are statistically up-regulated (normalized for total proteome) upon AO treatment (Fig. 2A). We next analyzed the data for the number of ubiquitylated mitochondrial proteins using the recently published MitoCarta 3.0 database containing data on 1140 mouse gene encoding proteins that are strongly localized to mitochondria including their submitochondrial location (*34*). Three hundred forty-five unique diGLY-containing Kgg peptides were detected in a total of 139 mitochondrial proteins under basal and induced conditions, of which 54 proteins were located in the MOM, 42 proteins were located in the mitochondrial inner membrane (MIM), 19 proteins were located in the matrix, 10 proteins are of unknown mitochondrial sublocation, 9 proteins are membrane associated, and 5 proteins are in the intermembranous space (IMS) (Fig. 2, B and C). The major portion of ubiquitylated proteins up-regulated by AO were MOM localized, accounting for 16.6% (MitoCarta, 21.3%) after 5 hours of mitochondrial depolarization (Fig. 2, B and C). We also detected Kgg peptides of several mitochondrial membrane proteins, Tudor and KH domain-containing protein (TDRKH), ATP-binding cassette sub-family D member 3 (ABCD3), Dephospho-CoA kinase domain-containing protein (DCAKD), and Dehydrogenase/reductase SDR family member 7B (DHRS7B); MIM proteins Retinol dehydrogenase 13 (RDH13) and CDP-diacylglycerol--glycerol-3-phosphate 3-phosphatidyltransferase (PGS1); and matrix proteins Glutamate dehydrogenase 1 (GLUD1) and 39S ribosomal protein L22 (MRPL22) that were increased about twofold or more following AO (table S2). On the basis of MS1-abundance information and TMT quantification, we performed a simple ranking analysis of Kgg peptides cross-referenced to MitoCarta 3.0 (fig. S7A) to determine the most abundant MOM ubiquitylation sites, and these included Carnitine O-palmitoyltransferase 1 (CPT1α) (K180, K195, and K508), Cytochrome b5 type B (CYB5B) (K30), Voltage-dependent anion-selective channel protein 1 (VDAC1) (K122 and K187), HK1 (K819, K243, and K247), CISD1 (K68 and K79), NADH-cytochrome b5 reductase 3 (CYB5R3) (K115 and K120), and Mitochondrial import receptor subunit TOM70 (TOMM70) (K171 and K240) (fig. S7A). Conversely, we also performed similar analysis on MOM ubiquitylation sites that are most reduced following AO treatment (fig. S7B). This revealed down-regulated sites for PARK7/DJ1 (K93), VDAC1 (K33), phoshoglycerate mutase 5 (PGAM5) (K73, K140, K161), and TOMM20 (K56, K61) (fig. S7B). Comparison of the basal ubiquitin levels of the most up-regulated and down-regulated MOM sites did not generally predict their subsequent modification following AO treatment; for example HK1, CYB5R3, and TOMM70 exhibited basal ubiquitin at sites in the range of 1.5 × 10^6^ but became highly modified (fig. S7, A and B).

Overall, 160 Kgg peptides from 70 mitochondrial proteins were significantly elevated by AO treatment (Fig. 2, B and D) and this compared to 112 Kgg peptides from 56 mitochondrial proteins that were significantly elevated by AO treatment in human iNeurons (*18*). Consistent with previous analysis in human iNeurons, we found that the majority of proteins were MOM localized (Fig. 2, B and C), and 51 ubiquitylation sites spanning 23 mitochondrial proteins were conserved between mouse neurons and human iNeurons, namely, acyl–coenzyme A synthetase long chain 1 (ACSL1), 1-acyl-sn-glycerol-3-phosphate acyltransferase epsilon (AGPAT5), Outer mitochondrial transmembrane helix translocase (ATAD1), CISD1, CPT1α, CYB5R3, Peroxiredoxin-like 2A (FAM213A), Mitochondrial fission 1 protein (FIS1), FKBP8 (FK506 binding protein 8), Ganglioside-induced differentiation-associated protein 1 (GDAP1), Growth hormone-inducible transmembrane protein (GHITM), HK1, MFN1/2, RAB24, RHOT1/2, Synaptojanin-2-binding protein (SYNJ2BP), TDRKH, TOMM70, and VDAC1/2/3 (Fig. 2, B and D). In addition, we identified two proteins whose ubiquitylation was increased in both mouse neurons and human iNeurons, although the sites were distinct, namely, the mitophagy receptor BCL2/adenovirus E1B 19 kDa protein-interacting protein 3-like (BNIP3L), DCAKD (Fig. 2D). Of mitochondrial substrates specific to mouse neurons, we interestingly detected ubiquitylation of enzymes linked to dopamine and catecholamine metabolism including monoamine oxidase A (MAO-A), MAO-B, and catechol-*O*-methyltransferase (COMT), which degrade dopamine and are established drug targets for symptomatic treatment of PD (table S2). Previous diGLY analysis found that MAOB was ubiquitylated in response to mitochondrial depolarization in HeLa, HCT116, and SH-SY5Y cells (*20*). COMT is expressed in iNeurons, but there was no increase in ubiquitylation, while MAO-A and MAO-B were not expressed (*18*).

Previous TMT analysis of HeLa cells (overexpressing Parkin) has indicated that activation of PINK1 and Parkin may stimulate recruitment of a large panoply of proteins to depolarized/damaged mitochondria, as determined by the detection of 137 ubiquitylation sites derived from 85 cytosolic proteins including Parkin and autophagy receptors such as OPTN and Tax1-binding protein 1 homolog (TAX1BP1) in mitochondrial-enriched fractions (*25*). Under endogenous conditions, we were unable to detect ubiquitylation sites for Parkin or autophagy receptors in our analysis but could detect unmodified peptides for OPTN and TAX1BP1 (table S2). Comparative analysis of datasets revealed approximately 80 cytosolic proteins whose ubiquitylation was increased upon AO treatment in both mouse neuron and HeLa cell analyses, and in a significant number of proteins, the site of ubiquitylation was identical between datasets (table S3). These common sites included the p97 ubiquitin-binding cofactor FAS-associated factor 2 (FAF2) (K167), the amyotrophic lateral sclerosis (ALS)–linked protein TAR DNA-binding protein 43 (TDP-43) (K181), the protein kinase AKT1 (K426), and Small ubiquitin-related modifier (SUMO) E3 ligase, Transcription intermediary factor 1-beta (TRIM28) (K320) (table S3 and fig. S7C) (*25*). In neurons, we also found a large number of ubiquitylation sites in cytosolic kinases that were up-regulated upon AO treatment including glycogen synthase kinase 3β, calcium/calmodulin-dependent protein kinase 2α/β/γ (CAMK2α/β/γ), several protein kinase C (PRKC) isoforms, Unc-51 like autophagy activating kinase 1 (ULK1) (K162), and LRRK2 (K1132) (table S4 and fig. S7C). Recent studies have indicated a role for ULK1 in the initiation of mitophagy (*35*, *36*), and LRRK2 has also been shown to regulate basal mitophagy (*37*); in future work, it will be interesting to determine whether other cytosolic kinase and phosphatase signaling components identified in our screen may be implicated in mitophagy mechanisms (table S4).

### Quantitative diGLY proteome analysis in mitochondria of wild-type and PINK1 KO mouse cortical neurons

**Fig. 3.**
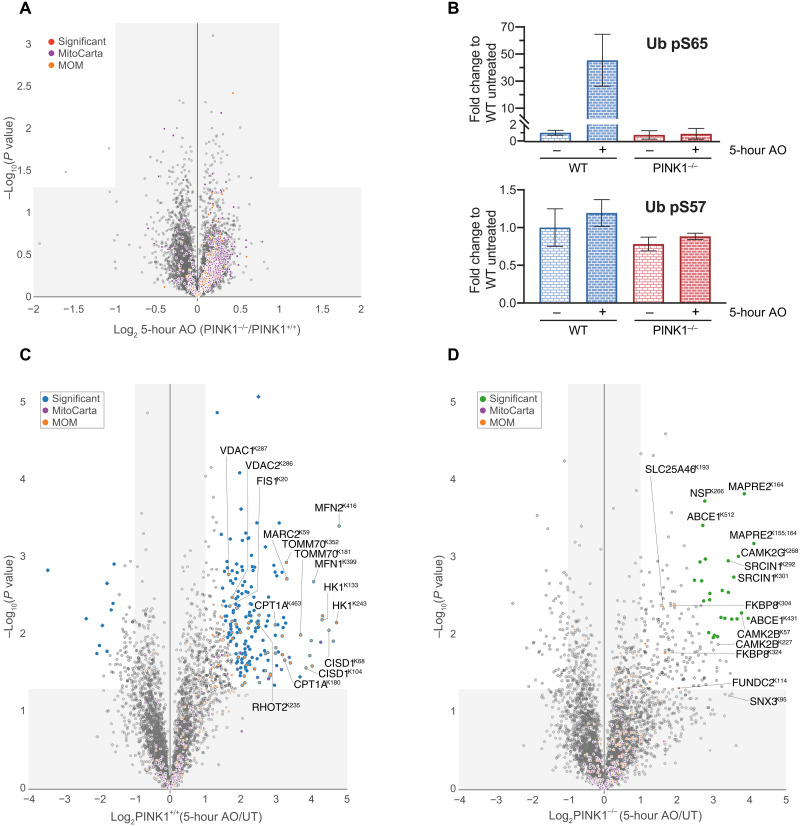
Global ubiquitylation analysis of mitochondria in neurons of PINK1 wild-type and KO neurons. (**A**) Total protein abundance of membrane-enriched lysates in PINK1^+/+^ or PINK1^−/−^ neurons. Fold increase for individual protein is shown in the volcano plot. The *x* axis specifies the fold changes, and the *y* axis specifies the negative logarithm to the base 10 of the *t* test *P* values [Welch’s *t* test (S0 = 0.585), corrected for multiple comparison by permutation-based FDR (5%)]. Proteins associated with mitochondria (MitoCarta 3.0) or MOM are indicated. (**B**) Abundance for phospho-Ser^65^ (top) or phospho-Ser^57^ (bottom) of ubiquitin (Ub) was quantified and plotted as fold change to untreated wild-type samples. Error bars represent SEMs (*n* = 3). Error bars represent SEMs (*n* = 3, 3, 3, and 2). (**C** and **D**) The same as (A) but diGLY-containing peptides derived from (C) PINK1^+/+^ or (D) PINK1^−/−^ cells. (C) One hundred eighty-seven and 11 sites or (D) 26 and 0 sites, respectively, represent statistically significant ubiquitylated targets up-regulated or down-regulated after mitochondrial depolarization. [Welch’s *t* test (S0 = 1), corrected for multiple comparison by permutation-based FDR (1%)]. diGLY peptides associated with mitochondria (MitoCarta 3.0) or MOM are indicated. Square-shaped dots indicate diGLY peptides not normalized to its protein abundance (not determined).

We next determined which ubiquitylated substrates were dependent on activation of PINK1 (fig. S8A). We isolated mitochondria from cultures of primary cortical neurons from crosses of wild-type and PINK1 KO mice that were untreated or treated with AO for 5 hours (fig. S8, A and B). We compared the proteome of triplicate cultures of wild-type and PINK1 KO mouse cortical neurons that were untreated or treated with AO for 5 hours. Similar to the previous experiment, we found very little change in the proteome abundance of wild-type neurons and the total proteome was slightly reduced in wild-type neurons following AO treatment, indicating increased turnover (Fig. 3A and fig. S8C). This mild reduction was completely blocked in the PINK1 KO neurons (Fig. 3A and fig. S8C). These results are consistent with similar observation of the proteome of wild-type and Parkin S65A iNeurons following AO treatment for 6 hours (*18*). Furthermore, quantitative TMT analysis demonstrated a ~50-fold increase in Ser^65^–phospho-ubiquitin upon AO in wild-type neurons that was abolished in PINK1 KO neurons (Fig. 3B). In parallel, we also measured Ser^57^–phospho-ubiquitin that is independent of AO treatment, and there was no difference between wild-type and PINK1 KO neurons (Fig. 3B).

We next determined the mitochondrial ubiquitylome in mouse neurons using diGLY affinity capture coupled with quantitative proteomics (fig. S6, A and B) (*18*). Tryptic peptides from mitochondrial enriched extracts of primary cortical neurons of wild-type and PINK1 KO mice with or without mitochondrial depolarization (5 hours) were subjected to α-diGLY immunoprecipitation, and samples were analyzed using 11-plex TMT-MS^3^ (wild type: three untreated/three AO; KO: three untreated/two AO) with diGLY peptide intensities normalized with total protein abundance measured in parallel (Fig. 3, C and D, and fig. S8, A and B) (see Materials and Methods).

We quantified a total of ~7210 diGLY-containing Kgg peptides of which ~3951 are unique sites (Fig. 3C). From these, we identified 177 ubiquitylation sites in 99 proteins in wild-type neurons after 5 hours of AO stimulation, whose abundance was significantly increased (Fig. 3C), and the majority of sites had been detected in AO-treated C57BL/6J neurons (Fig. 2). AO treatment of PINK1 KO mouse neurons for 5 hours led to substantially reduced AO-induced ubiquitylation of mitochondrial proteins including all but one of the common set of 23 MOM proteins (FKBP8) identified previously (Fig. 3D and fig. S6D). In contrast, we observed ubiquitylation of a number of cytosolic proteins including CAMK2α/β/γ and Sorting nexin-3 (SNX3) whose ubiquitylation was independent of PINK1 (Fig. 3D and fig. S8D). Principal components analysis revealed that the 5-hour depolarization data of wild type were not similar to the untreated wild-type and/or PINK1 KO samples, which were also more similar to each other (fig. S8E).

### Validation of Parkin-dependent substrates using cell-based and in vitro studies

**Fig. 4.**
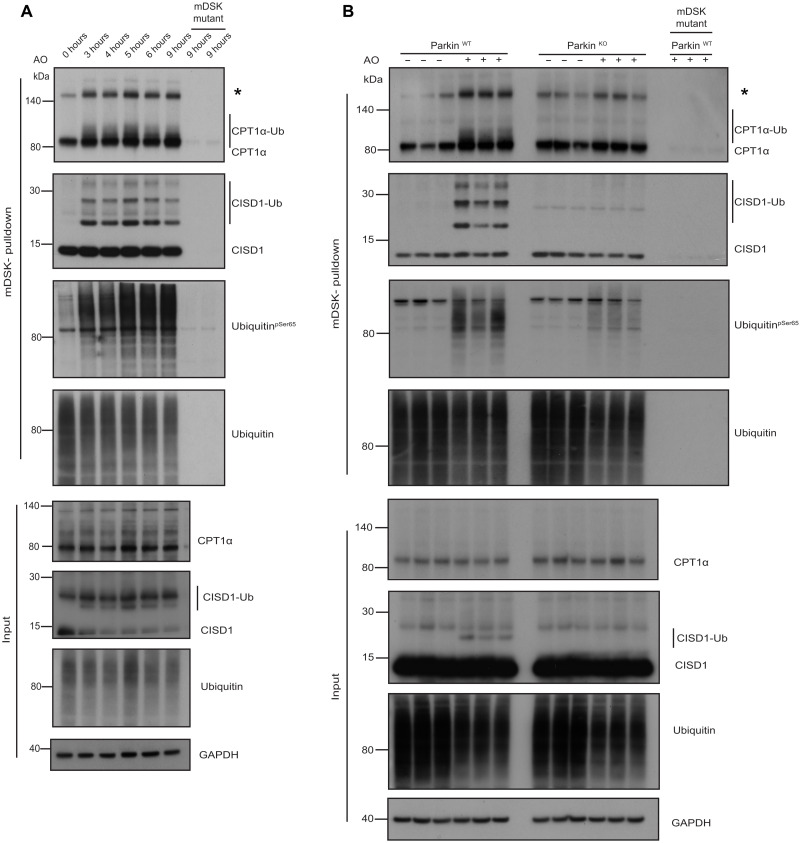
Validation of Parkin-dependent substrates in cell-based studies. (**A**) Time course analysis of CPT1α and CISD1 ubiquitylation following AO stimulation in C57BL/6J cortical neurons. Halo-multiDSK (mDSK) pulldown immunoprecipitated and inputs were subjected to immunoblot with anti-CPT1α, anti-CISD1, anti–phospho-Ser^65^ ubiquitin, and anti-ubiquitin antibodies. Asterisk (*) indicates high molecular weight CPT1α reactive species. (**B**) CPT1α and CISD1 ubiquitylation is abrogated in Parkin KO neurons. Membrane lysates of Parkin wild-type and KO cortical neurons after 5 hours of AO stimulation were subjected to ubiquitylated-protein capture by Halo-multiDSK (mDSK), before immunoblot with anti-CPT1α, anti-CISD1, anti–phospho-Ser^65^ ubiquitin and anti-ubiquitin antibodies. Relative inputs are shown at the bottom. Asterisk (*) indicates high molecular weight CPT1α reactive species.

Based on the ranking of the most abundant ubiquitylated (Kgg) mitochondrial sites (Fig. 2, A and B, and fig. S7A), we proceeded to investigate whether we could detect endogenous substrate ubiquitylation via biochemical analysis in wild-type C57BL/6J neurons that were either untreated or stimulated with AO for 5 hours. Mitochondrial-enriched fractions from neurons were subjected to TUBE pulldowns with HALO-UBA^UBQLN1^ or HALO-multiDSK (and nonbinding multiDSK mutant) and immunoblotted with specific antibodies for each substrate and phospho-ubiquitin to confirm Parkin activation (fig. S9). This confirmed previous observations (*28*, *29*) that CISD1 can undergo multi-monoubiquitylation upon AO treatment and the signal was similar for HALO-UBA^UBQLN1^ or HALO-multiDSK pulldowns. We also observed robust monoubiquitylation of CPT1α, an integral outer membrane protein that converts activated fatty acids into acylcarnitines and facilitates their transport into mitochondria (fig. S9) (*38*). This was more easily detected with HALO-multiDSK pulldown, and we used HALO-multiDSK to undertake a time course analysis and confirmed that CPT1α undergoes time-dependent ubiquitylation similar to CISD1 following AO treatment (Fig. 4A). We observed a high–molecular weight species of ~140 kDa that was immunoreactive to the CPT1α antibody following HALO-multiDSK pulldown (Fig. 4 and figs. S9 and S10A). This was present under basal conditions but increased after AO treatment. CPT1α is reported to form complexes at the MOM and oligomerize (*39*), and we cannot rule out that the upper band represents oligomerized species of ubiquitylated CPT1α.

We next assessed CPT1α ubiquitylation alongside CISD1 in triplicate biological replicates of Parkin KO (Fig. 4B) and PINK1 KO neurons (fig. S10A) with their respective wild-type littermate controls. Mitochondrial fractions from neurons untreated or stimulated with AO for 5 hours were subjected to TUBE pulldowns with HALO-multiDSK, demonstrating that CPT1α monoubiquitylation was abolished in Parkin and PINK1 KO neurons and this paralleled loss of CISD1 ubiquitylation (Fig. 4B and fig. S10A). Furthermore, the increased ubiquitylation of the ~140-kDa CPT1α-immunoreactive species was also attenuated in PINK1 and Parkin KO neurons. We also explored whether CPT1α ubiquitylation can independently be detected in a different cell system and analyzed human SH-SY5Y neuroblastoma cell lines that express endogenous PINK1 and Parkin (fig. S10B). Upon addition of AO, we observed time-dependent accumulation of CPT1α ubiquitylation that was maximal at 9 hours associated with an increase in endogenous phospho-Parkin (fig. S10B). From 9 to 24 hours, the levels of total Parkin and phospho-Parkin significantly reduced, and this was accompanied by a decrease in CPT1α ubiquitylation, consistent with its Parkin dependence (fig. S10B). The detection of AO-induced ubiquitylation of other mitochondrial proteins varied depending on the ubiquitin-affinity method used including TDRKH and ATAD1 that were selectively detected via HALO-UBA^UBQLN1^ pulldown (fig. S9). There were also several mitochondrial proteins exhibiting substantial basal ubiquitylation including AGPAT5, MFN2, and TOMM70 that prevented unambiguous detection of enhanced ubiquitylation upon AO treatment by our TUBE-based assay (fig. S9).

**Fig. 5.**
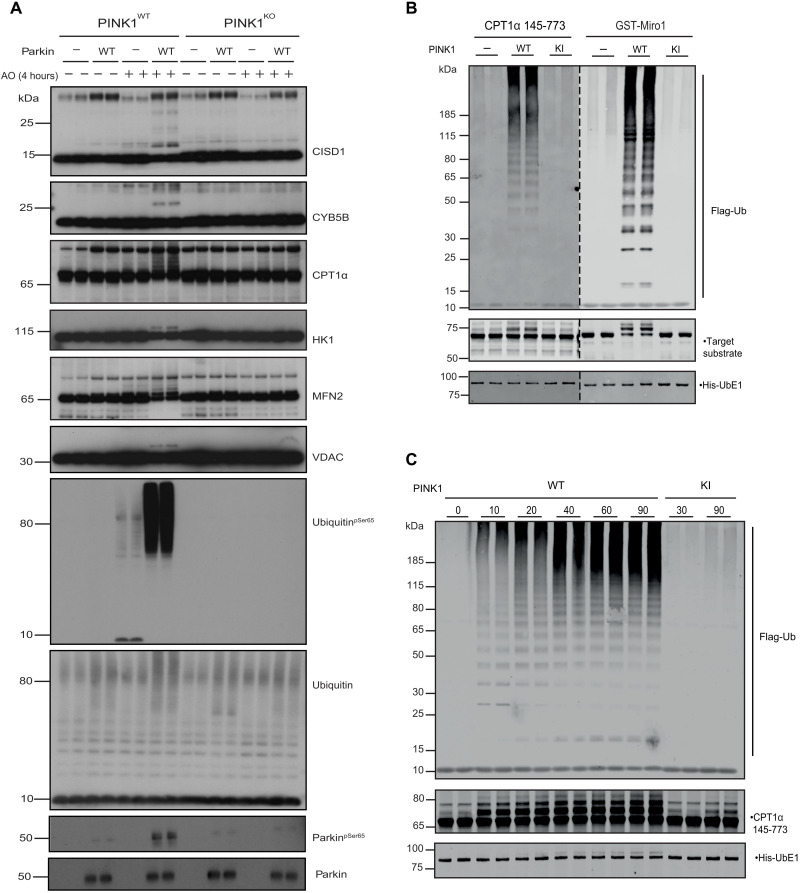
Validation of Parkin-dependent substrates in in vitro studies. (**A**) MEF PINK1 wild-type and KO were depolarized with AO for 4 hours; mitochondria were isolated and incubated with recombinant Parkin for in vitro ubiquitylation assay against endogenous mitochondrial substrates. Ubiquitylated proteins were detected with the indicated antibodies. (**B** and **C**) Parkin ubiquitylates CPT1α in vitro. (B) Recombinant CPT1α protein was assessed for ubiquitylation assay by Parkin with full-length PINK1 (WT), Kinase-inactive (KI), and water (-) as negative control. Miro1 recombinant protein was used as positive control. (C) Time course for CPT1α showed ubiquitylation mediated by Parkin in a time-dependent manner.

The stoichiometry of ubiquitylation of these substrates may also be at levels below the sensitivity of detection of this assay. To address this, purified mitochondrial extracts from wild-type or PINK1 KO MEFs (that express near-undetectable levels of endogenous Parkin) treated with or without AO were incubated together with or without recombinant human Parkin in vitro with a reaction mix containing E1 ubiquitin-activating ligase, UbcH7 conjugating E2 ligase, ubiquitin, and Mg-ATP. After 120 min, reactions were terminated with SDS sample buffer in the presence of 2-mercaptoethanol and heated at 100°C, and substrate ubiquitylation was assessed by immunoblot analysis with antibodies that detected CISD1, CPT1α, CYB5B, HK1, MFN2, and total VDAC (Fig. 5A). In the absence of AO treatment, PINK1 and Parkin were inactive in the assay with no evidence of phospho-ubiquitin or substrate ubiquitylation, respectively (Fig. 5A). In AO-stimulated mitochondria, addition of Parkin led to multi-monoubiquitylation of CISD1 and a smear of CPT1α ubiquitylation, consistent with the TUBE pulldown assays (Fig. 5A). Under these assay conditions, we were also able to observe robust monoubiquitylation of CYB5B, HK1, MFN2, and VDAC accompanied by substantial high molecular–weight phospho-ubiquitin signal (Fig. 5A). These signals were abolished in PINK1 KO cells, consistent with PINK1-dependent activation of Parkin in the assay (Fig. 5A). We observed that in AO-stimulated mitochondria without Parkin, ubiquitin was predominantly phosphorylated as mono-ubiquitin, indicating that PINK1 may initially target free mono-ubiquitin at the mitochondria before Parkin-catalyzed ubiquitin of substrates and chains that is consistent with our PRM analysis of DIV12 neurons where Parkin is expressed at low levels (fig. S5). To confirm that these results were also relevant to human mitochondria, we undertook similar analysis of HeLa cells (that express no Parkin) incubated with or without recombinant Parkin and again observed robust ubiquitylation of CISD1, CPT1α, CYB5B, HK1, MFN2, and VDACs only in AO-stimulated mitochondria incubated with Parkin and not in dimethyl sulfoxide (DMSO)–stimulated mitochondria with Parkin (fig. S11).

We have previously reported an entirely in vitro reconstitution assay of Parkin E3 ligase activity that monitors multi-monoubiquitylation of RHOT1/Miro1 (residues 1 to 592) and the formation of free poly-ubiquitin chains (*40*). To assess whether Parkin (activated by phosphorylation of PINK1) directly ubiquitylates CPT1α, we phosphorylated untagged full-length Parkin with the active insect ortholog of PINK1, *Tribolium castaneum* (TcPINK1), in the presence of ATP and then added a reaction mix containing E1 ubiquitin-activating ligase, UbcH7 conjugating E2 ligase, ubiquitin, Mg-ATP, and a fragment of CPT1α (residues 145 to 773) alongside glutathione *S*-transferase (GST)–Miro1 (residues 1 to 592) that were expressed and purified in *Escherichia coli* (fig. S12). After 30 min, reactions were terminated with SDS sample buffer in the presence of 2-mercaptoethanol and heated at 100°C; substrate ubiquitylation was assessed by immunoblot analysis with antibodies that detect ubiquitin (FLAG), CPT1α, and Miro1. Consistent with previous findings in the absence of PINK1 phosphorylation, Parkin was inactive, as no evidence of free ubiquitin chain formation or Miro1 ubiquitylation was observed; with the addition of wild-type TcPINK1, Miro1 multi-monoubiquitylation in addition to free polyubiquitin chain formation was observed (Fig. 5B). No significant Miro1 ubiquitylation or polyubiquitin chain formation was observed in the presence of the kinase-inactive TcPINK1 (D359A) (Fig. 5B). Under these conditions, we observed multi-monoubiquitylation of CPT1α, indicating that this is also a direct substrate of Parkin. To determine the temporal dynamics of CPT1α ubiquitylation, we undertook a time course analysis and observed the appearance of multi-monoubiquitylation at 10 min following addition of activated Parkin, and this increased in a time-dependent manner and paralleled polyubiquitin chain formation (Fig. 5C). We also tested additional mitochondrial substrates identified from our diGLY mass spectrometry (MS) screen including FAM213A, MAO-A, MAO-B, and ACSL1 (residues 46 to end) that forms a complex with CPT1α (and VDAC) to promote influx of activated fatty acids into the mitochondria (Fig. 2D and fig. S12) (*38*). All underwent mono-ubiquitylation following addition of PINK1-activated Parkin, further suggesting that Parkin preferentially catalyzes mono/multi-monoubiquitylation of substrates in vitro (fig. S13).

We next investigated whether nonmitochondrial proteins whose ubiquitylation was increased following AO treatment are direct Parkin substrates (Figs.2A and 3D and fig. S14A). Under similar conditions described above, we incubated SNX3, CAMK2α, and CAMK2β with activated Parkin in vitro [fig. S14, B to D (left)]. We observed free polyubiquitin chain formation but no significant substrate phosphorylation, indicating that these are not Parkin substrates and unlikely to be regulated by active Parkin in neurons. MS data did not reveal any difference in sites of ubiquitylation in these proteins between wild-type and PINK1 KO neurons following stimulation with AO, suggesting that additional ubiquitin E3 ligases may be activated and recruited to the mitochondria upon AO treatment in a PINK1-independent manner [Fig. 3D and fig. S14, B to D (right)].

## DISCUSSION

Our groups have previously demonstrated the utility of primary mouse neurons and human iNeurons to study endogenous Parkin activation including conservation of Parkin activation by PINK1-dependent phosphorylation of Ser^65^ (*18*, *19*, *29*). Combining a diGLY/TMT-MS^3^ workflow (*25*) in mouse neurons with comparative data from previous analysis in human iNeurons (*18*) has elaborated a defined set of endogenous substrates of Parkin conserved in mouse and human neuron cell types (Fig. 2). Notably, from many thousands of peptides detected by MS in the screen, we found 49 unique diGLY sites in 22 proteins in which ubiquitylation was increased greater than twofold or more in neurons upon activation of PINK1 by mitochondrial depolarization (Figs. 2 and 3). We also identified two proteins, BNIP3L and DCAKD, whose ubiquitylation was increased in both mouse and human iNeurons albeit at different sites (Fig. 2). Furthermore, all of the sites were present on proteins localized at the MOM, consistent with previous studies on how activation of PINK1 and Parkin on the MOM is sufficient to induce clearance of damaged mitochondria via mitophagy (*8*–*10*). This was associated with the enhanced formation of phospho-ubiquitin and K63 chain linkages in mouse neuronal mitochondria (Fig. 1, D and E), which was also found to be increased 70-fold in human iNeurons upon mitochondrial depolarization (*18*).

Of the common substrates identified in our neuronal systems, a number have been previously quantified in HeLa and human iNeurons including CISD1, HK1, MFN1/2, RHOT1/2, TOMM70, and VDAC1/2/3, and their collective ubiquitylation together with other substrates has been associated with mitophagic signaling and clearance (*18*, *19*). Our analysis also found ATAD1 (Msp1) to be ubiquitylated upon Parkin activation (Fig. 2). Akin to p97/Cdc48, Msp1 extracts misfolded proteins from the MOM, providing a direct link between a Parkin substrate and mitochondrial quality control component (*41*, *42*). We identified common ubiquitylation sites on FKBP8 (also known as FKBP38) and SYNJ2BP (also known as OMP25) that are both anchored to the MOM via translocase of outer mitochondrial Membrane (TOM)–independent “tail anchor” mitochondrial targeting signals within a C-terminal juxtamembrane sequence known as the CSS (*43*). The CSS of FKBP38 has also been demonstrated in MEFs to mediate its escape from the mitochondria to the endoplasmic reticulum (ER) during mitophagy, thereby avoiding its degradation, and this was also dependent on Parkin activity (*44*). However, in our studies, we found that FKBP8 was regulated independent of PINK1, suggesting that an alternate ubiquitin E3 ligase targets this protein, and in future studies, it will be interesting to assess whether FKBP8 undergoes escape to the ER in neurons and dissect the mechanism. Activation of Parkin in neurons may also be associated with proximal signaling events independent of mitophagy, and several of the common neuronal sites that we identified occur on proteins involved in fatty acid oxidation and metabolism in the mitochondria including CPT1α, ACSL1, VDACs, and CYB5R3 (Figs. 2 and 3). Recently, several in vivo physiological studies have suggested a link between fatty acid oxidation and mitophagy mechanisms in non-neuronal tissues (*45*, *46*), and in future work, it will be interesting to investigate whether Parkin-induced ubiquitylation of these proteins affects fatty acid metabolism at the molecular level in neurons.

Since this is the first study to define common substrates regulated by human and mouse Parkin, we undertook a multiple sequence alignment analysis of the human Lys-targeted sequences to identify a putative recognition motif for Parkin; however, this did not reveal a clear targeting sequence for Parkin-directed lysine ubiquitylation (fig. S15), consistent with previous studies that have suggested that Parkin lacks sequence and structural specificity for substrate ubiquitylation (*18*–*20*, *29*).

Previous analysis of VDAC1 has suggested that the topology of the protein at the MOM and surface exposure of Lys residues to the cytosolic side strongly influence Parkin targeting; however, it is not known if this is a general mechanism (*18*, *19*). Structural analysis to date has elaborated the mechanism of Parkin activation by PINK1 phosphorylation of Parkin and ubiquitin (*47*, *48*), and in future studies, it will be interesting to reconstitute Parkin-catalyzed ubiquitylation of model substrates and solve structures of Parkin bound to E2 and substrate to uncover any autonomous structural determinants of lysine recognition by Parkin, and our elaboration of its key substrates in neurons will aid in these studies.

The ubiquitylation of MOM proteins on damaged mitochondria is followed by the recruitment of the autophagy receptor proteins NDP52, p62, OPTN, Tax1bp, and Next to BRCA1 gene 1 protein (NBR1), and several studies suggests that NDP52 and OPTN are the main ubiquitin-dependent receptors for efficient mitophagic clearance of damaged mitochondria both in HeLa cells and neurons (*23*, *24*, *49*). The mechanism of how these are recruited to the MOM is not fully understood since NDP52 and OPTN have been demonstrated to bind diverse ubiquitin chain types including M1, K48, and K63 chains (*50*, *51*), and there are conflicting reports on how recruitment of these receptors is affected by phospho-ubiquitin (*19*, *23*, *50*, *52*). The Ub-binding domain in ABIN proteins and NEMO (UBAN) domain of OPTN preferentially binds M1 or K63-linked ubiquitin chains, and previous analysis has found that phosphorylation of OPTN by TANK-binding kinase 1 (TBK1) enhances its binding to ubiquitin chains (*52*). Phosphorylation of Ser^473^ not only enhanced affinity of OPTN to longer K63 chains but also promoted binding to other linkage types, which may not be relevant to mitophagic signaling in neurons (*24*, *52*). Our findings of defined substrates and specific accumulation of K63-linked ubiquitin chain types in neurons will be beneficial to studies underway to reconstitute mitophagy mechanisms in vitro (*53*, *54*) that may shed light on the initial steps of ubiquitin receptor recruitment to mitochondria. It was recently suggested that ubiquitin chains alone are sufficient to stimulate mitophagy (*55*), and our findings also lend tractability to undertake genetic analysis as to whether ubiquitylation of specific substrates is critical for mitophagy or not. FKBP8 has also been reported to act as mitophagy receptor in HeLa cells via an N-terminal LC3-interacting region that binds strongly to LC3A; however, this was independent of Parkin activation (*56*), and future studies should be directed at its role in mitophagy in neurons in light of our discovery of Parkin-dependent ubiquitylation.

A recent study has reported that two protein kinases, cyclin G-associated kinase (GAK) and PRKCδ, are previously unknown regulators of Parkin-independent mitophagy and recruit ULK1 to autophagosome structures close to mitochondria (*57*). While we did not detect either GAK or PRKCδ, we did detect multiple kinases in our mitochondrial enriched fractions that were ubiquitylated including PRKCβ, PRKCε, and PRKCγ isoforms, and in future work, it will be important to determine whether these and other kinases recruited to mitochondria regulate AO-induced mitophagy in neurons (table S5). Our analysis has also highlighted PINK1-independent and Parkin-independent ubiquitylation sites following AO stimulation. While BCL2/adenovirus E1B 19 kDa protein-interacting protein 3-like (NIX/BNIP3) and FUN14 domain-containing protein 1 (FUNDC1) receptor-mediated mitophagy have been linked to mitochondrial turnover during metabolic reprogramming or hypoxia, very little is known on PINK1-independent ubiquitin-mediated mechanisms regulating mitophagy (*5*). Our discovery of clear-cut PINK1-independent substrates suggests that there are parallel ubiquitin signaling pathways that are activated alongside PINK1-Parkin in neurons following AO treatment. We have compared our proteome dataset in neurons with the UbiHub online database (*58*) and found 40 E2, 165 “simple” E3, and 155 “complex” E3 components that are present within our mitochondrial-enriched neuron fractions, and we have deposited these data together with all detected ubiquitylation sites in a searchable online database that we have termed MitoNUb (https://shiny.compbio.dundee.ac.uk/MitoNUb/). Only two E3s, Mitochondrial ubiquitin ligase activator of NFKB 1 (MUL1) and F-box/LRR-repeat protein 4 (FBXL4), are located within the mitochondria, and the remainder are cytosolic; in future work, it will be interesting to define those that may be recruited and activated at the mitochondria upon AO treatment.

Intriguingly, DJ1 ubiquitylation was reduced by AO at Lys^93^ and, to a lesser extent, at Lys^130^ (fig. S7B). Both sites are highly conserved and lie close to Cys^106^ that is modified under oxidative stress conditions, thereby stimulating DJ1 recruitment from the cytosol to the mitochondria (fig. S16A) (*59*). Patients with DJ1 mutations resemble PINK1 and Parkin patients (*60*); however, we observed that the reduction of DJ1 ubiquitylation at Lys^93^ was PINK1 independent (fig. S16B), and in future work, it will be interesting to determine the mechanism of the functional impact of DJ1 ubiquitylation in the context of mitophagic signaling. We also observed many other diGLY sites that were down-regulated following mitochondrial depolarization, suggesting that mitochondrial depolarization may alter the activity of Deubiquitinating enzymes (DUBs) at the mitochondria by an as yet unknown mechanism.

Overall, this study together with previous findings in human iNeurons (*18*) provides a common signature of ubiquitylation events occurring in neuronal mitochondria that can be used to investigate Parkin activity in other neuronal cell types including dopaminergic neurons. The identification of a specific set of sites targeted by endogenous Parkin in cell types relevant to Parkinson’s will provide impetus to the development of facile proteomic and cell-based assays to monitor Parkin activity that will aid drug discovery and translational efforts to activate PINK1 and Parkin as a potential therapeutic strategy in PD (*61*).

## MATERIALS AND METHODS

### Antibodies for biochemical studies

The following primary antibodies were used: anti-Parkin phospho-Ser^65^ rabbit monoclonal antibody was raised by Epitomics/Abcam in collaboration with the Michael J. Fox Foundation for Research (please contact tools@michaeljfox.org for questions). The following antibodies were used: CISD1 (Cell Signaling Technology and Proteintech), glyceraldehyde-3-phosphate dehydrogenase (Santa Cruz Biotechnology), ubiquitin (BioLegend), CPT1α (Abcam), HK1 (Thermo Fisher Scientific and Cell Signaling Technology), GK (Abcam), DCAKD (Aviva Systems Biology), ABCD3 (Aviva Systems Biology), ACSL1 (Cell Signaling Technology), ACSL6 (Sigma-Aldrich), AGPAT5 (Abcam), MARC2 (Sigma-Aldrich), CYB5B (Novus Biologicals), CYB5R3 (Sigma-Aldrich), MFN1 (Abcam), MFN2 (Proteintech and Abcam), RHOT2 (Proteintech), TOMM70 (Aviva Systems Biology), SLC25A46 (Proteintech), FAM213A (Novus Biologicals), MAO-A (Proteintech), MAO-B (Abcam), HSDL1 (Proteintech), CAMK2α (Thermo Fisher Scientific), CAMK2β (Thermo Fisher Scientific), DCAMKL2 (Abcam), CAD (Novus), PRKCγ (Proteintech), ATAD1/Thorase (NeuroMab), TDRKH (Proteintech), FBXO41 (Proteintech), SNX3 (Sigma-Aldrich), CNN3 (Sigma-Aldrich), SH3BP4 (Novus Biologicals), MAPRE2 (Proteintech), RAB5C (MyBioSource), p23 (Thermo Fisher Scientific), VPS35 (Abcam), OPA1 (Cell Signaling Technology), Rab8A (Cell Signaling Technology), Rab8A phospho-Ser^111^ (Abcam), VDAC (Cell Signaling Technology), and horseradish-peroxidase–conjugated secondary antibodies (Sigma-Aldrich).

### Materials and reagents

HaloLink resin was purchased from Promega. All mutagenesis was carried out using the QuikChange site-directed mutagenesis method (Stratagene) with KOD polymerase (Novagen). All DNA constructs were verified by MRC Protein Phosphorylation and Ubiquitylation Unit (PPU) DNA Sequencing Service, School of Life Sciences, University of Dundee, using DYEnamic ET terminator chemistry (Amersham Biosciences) on Applied Biosystems automated DNA sequencers. DNA for bacterial protein expression was transformed into *E. coli* BL21 DE3 RIL (CodonPlus) cells (Stratagene). Stock solutions of antimycin A (Sigma-Aldrich) and oligomycin (Sigma-Aldrich) were used for experiments in cells. Unless otherwise specified, general reagents and chemicals were from Sigma-Aldrich (Merck), and cell culture reagents were from Gibco/Invitrogen (Thermo Fisher Scientific). All cDNA plasmids, antibodies, and recombinant proteins generated in-house for this study are available on request through our dedicated reagents website: https://mrcppureagents.dundee.ac.uk/. All newly generated resources are being registered with rrids.org/, and research resource identifier are included in the list of the key resources in the supplementary materials.

### Primary cortical neuron preparation and culture

Primary mouse cortical neurons were isolated from the brains of C57BL/6J embryos of either sex at E16.5. Embryonic cortices were collected in Hanks’ balanced salt solution, and cells were dissociated by incubation with trypsin-EDTA (#25300-054, Gibco) at 37°C. Cells were then diluted in Neurobasal medium containing B27 supplement, GlutaMAX, and penicillin-streptomycin and plated at a density of 5.0 × 10^5^ cells per well on six-well plates coated with poly-l-lysine (0.1 mg ml^−1^; Sigma-Aldrich). Neurons were cultured at 37°C in a humidified incubator with 5% CO_2_. Every 5 days, the medium was replaced with fresh medium containing B27. To depolarize mitochondrial membrane potential in neurons, cultures were treated for 5 hours with 10 μM antimycin A (Sigma-Aldrich) and 1 μM oligomycin (Sigma-Aldrich) in DMSO at 37°C. A detailed protocol describing the preparation of primary cortical mouse neurons has been reported (dx.doi.org/10.17504/protocols.io.bswanfae).

### Immunofluorescence and confocal microscopy

Mouse cortical neurons were seeded on poly-l-lysine–coated glass coverslips and were fixed for 20 min on ice in 4% paraformaldehyde (Sigma-Aldrich)/4%sucrose solution in phosphate-buffered saline (PBS; Gibco). Neurons were permeabilized for 1 hour in blocking solution, containing 0.2% Triton X-100 (Sigma-Aldrich) and 10% donkey serum (Sigma-Aldrich), and incubated overnight at 4°C with the primary antibodies MAP2 (1:100; Sigma-Aldrich, M2320) and GFAP (1:100; Abcam, ab7260) in blocking solution. Then, neurons were washed with PBS and incubated for 1 hour at room temperature with Hoechst and with anti-rabbit or anti-mouse secondary antibodies conjugated with Alexa Fluor 488 and Alexa Fluor 594 (1:1000; Thermo Fisher Scientific) in blocking solution. After three washes in PBS, neurons were mounted using VECTASHIELD. Images were acquired with an LSM 710 laser scanning confocal microscope (ZEISS; Plan-Neofluar ×40 objective) using ZEISS Zen Software. Images were quantified for MAP2-positive and GFAP-positive cells using Volocity 3D Image Analysis Software (Quorum Technologies, Ontario) using a custom written protocol.

### High-resolution respirometry of primary neurons

Primary mouse cortical neurons from PINK1 KO embryos and their wild-type littermates were cultured for DIV21. Neurons were washed twice with PBS and treated with 0.05% trypsin for 15 min, after which trypsin was neutralized with Dulbecco’s modified Eagle’s medium (DMEM)/nutrient mixture F-12 medium. Neurons were gently spun down (300*g* for 3 min), and the cells of two wells (of six well plates) were resuspended in DMEM F12 and used for high-resolution respirometry. After cell number determination, cells were gently spun down (300*g* for 3 min). The supernatant was aspirated, and 50 μl of MiR05 medium [110 mM sucrose, 60 mM lactobionic acid, 0.5 mM EGTA, 3 mM MgCl_2_, 20 mM taurine, 10 mM KH_2_PO_4_, 20 mM Hepes adjusted to pH 7.1 with KOH at 30°C, and bovine serum albumin (BSA; 1 g/liter) essentially fatty acid free] was added to the pellet. Pellets were resuspended (using a 1-ml tip to avoid damaging the cells) and placed in an oxygraphic chamber thermostated at 37°C with continuous stirring (Oxygraph-2k, Oroboros Instruments, Innsbruck, Austria). Mitochondrial respiratory rates were assessed using the substrate-uncoupler-inhibitor titration protocol number 2 (SUIT-002) (*62*). Briefly, after residual oxygen consumption (ROX; in the presence of 2.5 mM adenosine diphosphate) in the absence of endogenous fuel, substrates were measured; fatty acid oxidation pathway state (F) was measured by adding 0.1 mM malate and 0.2 mM octanoyl carnitine (OctMP). Membrane integrity was then evaluated by adding 10 μM cytochrome c (OctMcP). Subsequent to the F-pathway state, the reduced form of nicotinamide adenine dinucleotide electron transfer-pathway state (FN) was studied by adding 2 mM malate (OctM_P_), 5 mM pyruvate (OctPM_P_), and 10 mM glutamate (OctPGM_P_). Then, 10 mM succinate (OctPGMS_P_) was added to subsequently stimulate the S pathway (FNS), followed by 10 mM glycerophosphate (OctPGMSGp_P_) to reach convergent electron flow in the FNSGp pathway to the Q-junction. Uncoupled respiration was then evaluated by realizing a titration with CCCP (OctPGMSGp_E_). Complex I was then inhibited with 0.5 μM rotenone (SGp_E_), and last, ROX was measured by adding 2.5 μM antimycin A. ROX was then subtracted from all respiratory states to obtain mitochondrial respiration. Results are expressed in pmol · s^−1^ · 10^6^ cells. The P/E control ratio, which reflects the control by coupling and limitation by the phosphorylation system, was subsequently calculated by dividing the OctPGMSGp_P_ value by the OctPGMSGp_E_ value.

### Isolation of mitochondrial enriched membrane fraction

Cells were collected in ice-cold PBS containing sodium orthovanadate (1 mM), sodium glycerophosphate (10 mM), sodium fluoride (50 mM), sodium pyrophosphate (10 mM), phenylmethylsulfonyl fluoride (PMSF; 0.1 mM) protease inhibitor cocktail, and 200 mM chloroacetamide and centrifuged at 500*g* for 3 min at 4°C. Cell pellets were resuspended and lysed in homogenization buffer containing 250 mM sucrose, 300 mM imidazole, 1 mM sodium orthovanadate, phosphatase inhibitor cocktail 3 (Sigma-Aldrich), and protease inhibitor cocktail (Roche) and supplemented with 200 mM chloroacetamide at 4°C. Cells were disrupted using a metal handheld homogenizer (40 passes), and the lysates were clarified by centrifugation (2000 rpm at 4°C for 5 min). The supernatant was harvested and subjected to an additional centrifugation step at 40000 rpm for 1 hour at 4°C. The resulting pellet containing the membrane-enriched fraction that was resuspended in Mitobuffer [270 mM sucrose, 20 mM Hepes, 3 mM EDTA, 1 mM sodium orthovanadate, 10 mM sodium β-glycerophosphate, 50 mM sodium fluoride, 5 mM sodium pyrophosphate (pH 7.5), and protease inhibitor cocktail (Roche) supplemented with 200 mM chloroacetamide at 4°C] and solubilized with a probe sonicator (5 s, 20% amplitude). A detailed protocol describing the isolation of mitochondrial-enriched fractions from cortical mouse neurons has been reported (dx.doi.org/10.17504/protocols.io.bxjupknw).

### Whole-cell lysate preparation

Primary cortical neurons and SH-SY5Y cells were sonicated in lysis buffer containing tris**·**HCl (50 mm, pH 7.5), EDTA (1 mM), EGTA (1 mM), Triton (1%, w/v), sodium orthovanadate (1 mM), sodium glycerophosphate (10 mM), sodium fluoride (50 mM), sodium pyrophosphate (10 mM), sucrose (0.25 mM), benzamidine (1 mM), PMSF (0.1 mM) protease inhibitor cocktail (Roche), phosphatase inhibitor cocktail 2 and 3 (Sigma-Aldrich), and chloroacetamide (200 mM). Following sonication, lysates were incubated for 30 min on ice. Samples were spun at 20,800*g* in an Eppendorf 5417R centrifuge for 30 min. Supernatants were collected, and protein concentration was determined by using the Bradford kit (Pierce). A detailed protocol describing the lysis of primary cortical mouse neurons has been reported (dx.doi.org/10.17504/protocols.io.bsvcne2w).

### Ubiquitin enrichment

For ubiquitylated protein capture, Halo-tagged UBDs of TUBE, multi-DSK (yeast Dsk1 ubiquitin binding protein) and multi-DSK mutant were incubated with HaloLink resin (200 μl; Promega) in binding buffer [50 mm tris**·**HCl (pH 7.5), 150 mm NaCl, and 0.05% NP-40] overnight at 4°C. Membrane-enriched fraction (400 μg) was used for pulldown with HALO-UBDs. Halo tube beads (20 μl) were added to neuronal membrane enrichment and incubated ON at 4°C. The beads were washed three times with lysis buffer containing 0.25 mM NaCl and eluted by resuspension in 1× lithium dodecyl sulfate (LDS) sample buffer (20 μl) with 1 mM dithiothreitol (DTT) or 2.5% 2-mercaptoethanol. The method for ubiquitin capture has previously been reported for Halo-tagged UBDs of TUBE (*63*) and multi-DSK (yeast Dsk1 ubiquitin binding protein) (*64*).

### PINK1 immunoprecipitation

For immunoprecipitation of endogenous PINK1, 500 μg of whole-cell lysate or membrane fraction was incubated overnight at 4°C with 10 μg of PINK1 antibody (S774C; MRC PPU Reagents and Services) coupled to Protein A/G beads (10 μl of beads per reaction; Amintra) as previously reported (*65*). The immunoprecipitants were washed three times with lysis buffer containing 150 mM NaCl and eluted by resuspending in 10 μl of 2× LDS sample buffer and incubating at 37°C for 15 min under constant shaking (2000 rpm) followed by the addition of 2.5% (by volume) 2-mercaptoethanol.

### Immunoblotting

All the samples were subjected to SDS–polyacrylamide gel electrophoresis (SDS-PAGE; bis-tris 4 to 12% gels), CAD, and Nav1 proteins were separated by using tris-acetate (3 to 8% gels), and all the gels were transferred onto Protran 0.45 polyvinylidene difluoride membranes (Immobilon-P). Membranes were blocked for 1 hour at room temperature with 5% nonfat milk or BSA in tris-buffer saline + 0.1% Tween20 [TBS-T; 50 mM tris**·**HCl and 150 mM NaCl (pH 7.5)] containing 0.1% Tween-20 and probed with the indicated antibodies overnight at 4°C. Detection was performed using horseradish peroxidase–conjugated secondary antibodies and enhanced chemiluminescence reagent. A detailed protocol describing the biochemical analysis of PINK1-Parkin pathway in primary cortical neurons by immunoblotting has been reported (dx.doi.org/10.17504/protocols.io.bswanfae).

### Parkin ubiquitylation assay

In vitro ubiquitylation assays were performed using recombinant proteins purified from *E. coli* unless stated otherwise. Wild-type Parkin (0.75 μM) was incubated for 30 min at 37°C in a thermo shaker at 1000 rpm with 0.36 μM wild-type or kinase-inactive (D359A) Maltose-binding protein (MBP)–TcPINK1 (*T. castaneum* PINK1) in 15 μl of kinase buffer [50 mM tris-HCl (pH 7.5), 0.1 mM EGTA, 10 mM MgCl_2_, and 0.1 mM ATP]. The ubiquitin master mix (50 mM tris-HCl, 10 mM MgCl_2_, 2 mM ATP, 0.12 μM His-UbE1 expressed in Sf21 insect cells, 1 μM human UbE2L3, 50 μM Flag-ubiquitin, and 0.5 μM substrate) was added to a final volume of 30 μl, and the reaction was incubated at 37°C for 30 min in a thermo shaker at 1000 rpm. Reactions were terminated by the addition of 4× LDS loading buffer, and 5 μl of the final volume was resolved using SDS-PAGE on a 4 to 12% bis-tris gel and immunoblotted using an anti-FLAG, anti-His, or antibodies against the substrate being tested.

Samples were resolved using SDS-PAGE on 4 to 12% bis-tris gels in Mops buffer and transferred to nitrocellulose membranes. Membranes were blocked with 5% (w/v) milk powder in TBS + 0.1% Tween-20 (TBS-T) for 1 hour at room temperature and then immunoblotted against the primary antibody in 5% (w/v) BSA/TBS-T at 5° to 7°C overnight. Protein bands were detected by blotting against secondary antibodies labeled with 800- or 680-nm fluorophores in TBS-T for 1 hour at room temperature and imaged using LI-COR.

A detailed protocol describing the expression of MBP-TcPINK1 has been reported (dx.doi.org/10.17504/protocols.io.bsrend3e). A detailed protocol describing the recombinant Parkin in vitro ubiquitylation assay has been reported (dx.doi.org/10.17504/protocols.io.bsfrnbm6).

### Recombinant protein expression

#### Parkin

His_6_-SUMO cleaved wild-type Parkin was expressed on the basis of the method (*12*). Briefly, plasmids were transformed in BL21-CodonPlus (DE3)-RIL *E. coli*, overnight cultures were prepared and used to inoculate 12× 1 liter of LB medium containing carbenicillin (50 μg/ml) and 0.25 mM ZnCl_2_. These were initially incubated at 37°C until the cultures reached an optical density at 600 nm (OD_600_) of 0.4; the incubator temperature was lowered to 15°C, and once cultures reached an OD_600_, of 0.8 to 0.9 expression was induced by the addition of 25 μM isopropyl-β-d-thiogalactopyranoside (IPTG). After overnight incubation (16 hours), cells were pelleted by centrifugation (4200*g*, 25 min), the medium was removed, and the cell pellet was suspended in lysis buffer [50 mM tris-HCl (pH 7.5), 250 mM NaCl, 15 mM imidazole (pH 7.5), and 0.1 mM EDTA with 1 μM 4-(2-aminoethyl)benzenesulfonyl fluoride hydrochloride (AEBSF) and leupeptin (10 μg/ml) added]. Cells were burst by sonication; cell debris were pelleted by centrifugation (35,000*g* for 30 min at 4°C), and the supernatant was incubated with Ni–nitrilotriacetic acid (Ni-NTA) resin for 1 hour at 5° to 7°C. Ni-NTA resin was washed five times in 7× the resin volume of lysis buffer and twice in 7× the resin volume of cleavage buffer [50 mM tris (pH 8.3), 200 mM NaCl, 10% glycerol, and 1 mM TCEP (tris(2-carboxyethyl)phosphine)]. Parkin was cleaved from the resin at 4°C overnight by the addition of a 1:5 mass ratio of His-SUMO/Sentrin Specific Peptidase 1 (SENP1) to total protein mass bound to the resin. After cleavage, Parkin was concentrated and further purified using size exclusion chromatography on a Superdex S200 column (16/600). Parkin was eluted after 80- to 90-ml fractions were pooled and concentrated, and the purity was tested using SDS-PAGE.

#### *Cpt1*α

A catalytic domain-containing fragment of Cpt1α (residues 145 to 773; missing the transmembrane domain) was expressed as His-SUMO–tagged fusion protein. The cDNA of the protein coding sequences was subcloned into pET15b plasmid vectors and transformed into BL21-DE3. A 150-ml starter culture was inoculated using a swipe from the cell plate and incubated at 37°C overnight. One liter of LB medium (12×) containing carbenicillin (50 μg/ml) was each inoculated with 20 ml of starter culture. When cell cultures reached an OD_600_ of 0.8 to 0.9 and a temperature of 15°C, protein expression was induced by the addition of 50 μM IPTG. Cell cultures were left to express at 15°C overnight (~16 hours) and then harvested by centrifugation at 5020*g* for 25 min; the medium was removed, and the cell pellet was suspended in 25 ml per 1 liter (~5-ml pellet) of lysis buffer [50 mM tris-HCl (pH 7.5), 250 mM NaCl, 15 mM imidazole (pH7.5), and 0.1 mM EDTA with 1 μM AEBSF and leupeptin (10 μg/ml) added]. Cells were burst by sonication at 50% amplitude on ice using 6× 15 s pulses; glycerol (10%) was added, and the lysis buffer was made up to 500 mM NaCl. Cell debris were pelleted by centrifugation at 35,000*g* and 4°C for 30 min; then, the supernatant layer was transferred to Ni-NTA resin. The supernatant was incubated with Ni-NTA resin for 1 hour at 5° to 7°C and washed six times in 7× the resin volume of wash buffer [50 mM tris-HCl (pH 7.5), 500 mM NaCl, 15 mM imidazole (pH 7.5), 0.5 mM TCEP, and 10% glycerol]; then, His-SUMO-Cpt1α 145-773 was eluted by incubation with 4× the resin volume of elution buffer (wash buffer containing 400 mM imidazole) at 5° to 7°C for 1 hour. The solvent layer was dialyzed against 5 liters of dialysis buffer [50 mM tris-HCl (pH 7.5), 200 mM NaCl, 0.5 mM TCEP, and 10% glycerol] overnight at 4°C with a 1:10 mass ratio of His-SENP1:eluted protein mass added. Uncleaved Cpt1α and contaminants were depleted by incubating with Ni-NTA resin for 1 hour at 5° to 7°C. The solvent layer was concentrated and purified using size exclusion chromatography in dialysis buffer using a 16/600 Superdex SD200 column. Fractions containing Cpt1α 145-773 (peak at ~75 ml) were pooled and concentrated to give the recombinant.

#### GST-Miro1

GST-Miro1 cDNA was subcloned into a pGEX6 plasmid vector and transformed into BL21-CodonPlus (DE3)-RIL *E. coli*. A 150-ml starter culture was inoculated using a swipe from the cell plate and incubated at 37°C overnight. One liter of LB medium (12×) containing carbenicillin (50 μg/ml) was each inoculated with 20 ml of started culture. When cell cultures reached an OD_600_ of 0.8 to 0.9 and a temperature of 15°C, protein expression was induced by the addition of 50 μM IPTG. Cell cultures were left to express at 15°C overnight (~16 hours) and then harvested by centrifugation at 5020*g* for 25 min; the medium was removed, and the cell pellet was suspended in 25 ml per 1 liter (~5-ml pellet) of lysis buffer [50 mM tris-HCl (pH 7.5), 250 mM NaCl, 1 mM β-mercaptoethanol, and 0.1 mM EDTA with 1 μM AEBSF and leupeptin (10 g/ml) added]. Cells were burst by sonication at 50% amplitude on ice using 6× 15-s pulses. Cell debris were pelleted by centrifugation at 35,000*g* and 4°C for 30 min; then, the supernatant layer was transferred to glutathione resin. The supernatant was incubated with the GSH resin for 1 hour at 5° to 7°C; the GSH resin was washed six times in 14× the resin volume of wash buffer [50 mM tris-HCl (pH 7.5), 200 mM NaCl, 0.5 mM TCEP, and 10% glycerol], and the protein was eluted by incubation with wash buffer containing 10 mM glutathione for 1 hour at 5° to 7°C. The eluted supernatant was dialyzed against 5 liters of wash buffer at 5° to 7°C overnight and concentrated, and the final sample was flash-frozen.

#### ACSL1 and SNX3

Cleaved ACSL1 (missing the N-terminal transmembrane domain) and SNX3 were expressed as a His_6_-SUMO tagged constructs. cDNA of the protein coding sequences were subcloned into pET15b plasmid vectors and transformed into BL21-CodonPlus (DE3)-RIL *E. coli*. A 100-ml starter culture was inoculated using a swipe from the cell plate and incubated at 37°C overnight. One liter of LB medium (6×) containing carbenicillin (50 μg/ml) was each inoculated with 20 ml of started culture. When cell cultures reached an OD_600_ of 0.8–0.9 and a temperature of 15°C, protein expression was induced by the addition of 100 μM IPTG. Cell cultures were left to express at 15°C overnight (~16 hours) and then harvested by centrifugation at 5020*g* for 25 min; the medium was removed, and the cell pellet was suspended in 25 ml per 1 liter (~5-ml pellet) of lysis buffer [50 mM tris-HCl (pH 7.5), 250 mM NaCl, 15 mM imidazole (pH 7.5), and 0.1 mM EDTA with 1 μM AEBSF and leupeptin (10 μg/ml) added]. Cells were burst by sonication at 50% amplitude on ice using 6× 15-s pulses; glycerol (10%) was added, and the lysis buffer was made up to 500 mM NaCl. Cell debris were pelleted by centrifugation at 35,000*g* and 4°C for 30 min; then, the supernatant layer was transferred to Ni-NTA resin. The supernatant was incubated with Ni-NTA resin for 1 hour at 5° to 7°C, washed six times in 7× the resin volume of wash buffer [50 mM tris-HCl (pH 7.5), 500 mM NaCl, 15 mM imidazole (pH 7.5), 0.5 mM TCEP, and 10% glycerol] and then two times in 7× the resin volume of cleavage buffer [50 mM tris-HCl (pH 7.5), 200 mM NaCl, 0.5 mM TCEP, and 10% glycerol]. The resin was incubated overnight without agitation at 4°C in 4× the resin volume of cleavage buffer with a 1:10 mass ratio of His-Senp1:bound protein. The solvent layer was depleted against a further 1 ml of Ni-NTA resin at 5° to 7°C for 1 hour. The final solution was concentrated, flash-frozen in liquid nitrogen, and stored at −80°C.

#### *MBP-CamK2*α *and MBP-CamK2*β

CamK2α and CamK2β were expressed as MBP-tagged constructs. cDNAs of the protein coding sequences were subcloned into pMEX3Cb plasmid vectors and transformed into BL21-CodonPlus (DE3)-RIL *E. coli*. A 100-ml starter culture was inoculated using a swipe from the cell plate and incubated at 37°C overnight. One liter of LB medium (6×) containing carbenicillin (50 μg/ml) was each inoculated with 20 ml of started culture. When cell cultures reached an OD_600_ of 0.8 to 0.9 and a temperature of 15°C, protein expression was induced by the addition of 400 μM IPTG. Cell cultures were left to express at 15°C overnight (~16 hours) and then harvested by centrifugation at 5020*g* for 25 min; the medium was removed, and the cell pellet was suspended in 25 ml per 1 liter (~5-ml pellet) of lysis buffer [50 mM tris-HCl (pH 7.5), 250 mM NaCl, and 0.1 mM EDTA with 1 μM AEBSF and leupeptin (10 μg/ml) added]. Cells were burst by sonication at 50% amplitude on ice using 6× 15-s pulses; glycerol (10%) was added, and the lysis buffer was made up to 500 mM NaCl. Cell debris were pelleted by centrifugation at 35,000*g* and 4°C for 30 min. The supernatant was incubated with amylose resin for 1 hour at 5° to 7°C, washed six times in 7× the resin volume of lysis buffer, and then eluted in elution buffer [50 mM tris-HCl (pH 7.5), 500 mM NaCl, 10 mM maltose, 0.5 mM TCEP, and 10% glycerol]. The eluted sample was dialyzed overnight against 5 liters of sample buffer [50 mM tris-HCl (pH 7.5), 0.1 mM EGTA, 150 mM NaCl, 0.1% ß-mercaptoethanol, 270 mM sucrose, and 0.03% Brij-35]. The final solution was concentrated, flash-frozen, in liquid nitrogen, and stored at −80°C.

#### *GST-Fam213A, GST–MAO*-*A, and GST–MAO-B*

Fam213A, MAO-A, and MAO-B were expressed as GST-tagged constructs. cDNAs of the protein coding sequences were subcloned into pGEX6 plasmid vectors and transformed into BL21-CodonPlus (DE3)-RIL *E. coli*. A 100-ml starter culture was inoculated using a swipe from the cell plate and incubated at 37°C overnight. One liter of LB medium (6×) containing carbenicillin (50 μg/ml) was each inoculated with 20 ml of started culture. When cell cultures reached an OD_600_ of 0.8 to 0.9 and a temperature of 15°C, protein expression was induced by the addition of 50 μM IPTG. Cell cultures were left to express at 15°C overnight (~16 hours) and then harvested by centrifugation at 5020*g* for 25 min; the medium was removed, and the cell pellet was suspended in 25 ml per 1 liter (~5-ml pellet) of lysis buffer [50 mM tris-HCl (pH 7.5), 250 mM NaCl, and 0.1 mM EDTA with 1 μM AEBSF and leupeptin (10 μg/ml) added]. Cells were burst by sonication at 50% amplitude on ice using 6× 15-s pulses; glycerol (10%) was added, and the lysis buffer was made up to 500 mM NaCl. Cell debris were pelleted by centrifugation at 35,000*g* and 4°C for 30 min. The supernatant was incubated with GSH resin for 1 hour at 5° to 7°C, washed six times in 7× the resin volume of lysis buffer, and then eluted in elution buffer [50 mM tris-HCl (pH 7.5), 500 mM NaCl, 10 mM reduced glutathione, 0.5 mM TCEP, and 10% glycerol]. The eluted sample was dialyzed overnight against 5 liters of sample buffer [50 mM tris-HCl (pH 7.5), 0.1 mM EGTA, 150 mM NaCl, 0.1% β-mercaptoethanol, 270 mM sucrose, and 0.03% Brij-35]. The final solution was concentrated, flash-frozen in liquid nitrogen, and stored at −80°C.

### Reconstitution of Parkin ubiquitylation activity in vitro in purified mitochondria

#### Mitochondrial isolation

Wild-type and PINK1 KO MEFs or Hela cells were stimulated with 10 μM antimycin and 1 μM oligomycin (4 hours for MEFs and 2 hours for HeLa) to induce PINK1 stabilization on mitochondrial outer membrane. Cell were washed twice with ice-cold PBS and resuspended in hypotonic buffer containing 20 mM Hepes (pH 7.8), 5 mM KCl, 1.5 mM MgCl_2_, 2 mM DTT, 1 mM PMSF, and protease inhibitor cocktail (Roche) for 15 min on ice. Cells were disrupted using a metal handheld homogenizer (45 passes for MEFs and 25 passes for HeLa); then, 2.5× mannitol-sucrose buffer [2.5× MSH; 20 mM Hepes (pH 7.8), 525 mM mannitol, 175 mM sucrose, 5 mM EDTA, 1 mM PMSF, and protease inhibitor cocktail (Roche)] was added to the disrupted cells, and the cell homogenates were clarified by centrifugation (700*g* at 4°C for 10 min) to remove nuclei and cell debris. Supernatants were collected and spun down at 9000*g* for 10 min. Mitochondrial pellets were resuspended and washed twice in 1× MSH [20 mM Hepes (pH 7.8), 210 mM mannitol, 70 mM sucrose, 2 mM EDTA, 1 mM PMSF, and protease inhibitor cocktail (Roche)] and centrifuged at 9000*g* for 10 min at 4°C. In the final step, mitochondrial pellets were resuspended in Mito ubiquitylation buffer [MUB; 50 mM tris HCl (pH 7.5), 70 mM sucrose, 210 mM sorbitol, 50 mM sodium fluoride, 5 mM sodium pyrophosphate, and 10 mM sodium 2-glycerophosphate]. A detailed protocol describing the purification of mitochondria for in vitro ubiquitylation assay has been reported (dx.doi.org/10.17504/protocols.io.bxmypk7w).

#### Ubiquitylation assay

Mitochondria were isolated from cells and resuspended in MUB. Mitochondrial preparations (5 μg) were used as substrates for the in vitro ubiquitylation assay. Wild-type Parkin (1 μM) was incubated with 0.1 μM His-UbE1 expressed in Sf21 insect cells, 1 μM human UbE2L3, and 30 μM ubiquitin for 120 (MEFs) or 90 min (HeLa) at 30°C in a thermo shaker at 1000 rpm in 50 μl of reaction buffer [50 mM tris-HCl (pH 7.5), 5 mM MgCl_2_, 0.5 mM TCEP, and 2 mM ATP]. Reactions were terminated by the addition of 4× LDS loading buffer, containing 10% 2-mercaptoethanol. The final volume (20 μl) was resolved using SDS-PAGE on a 4 to 12% bis-tris gel and immunoblotted using antibodies against the substrate being tested. A detailed protocol describing the in vitro ubiquitylation assay using purified mitochondria has been reported (dx.doi.org/10.17504/protocols.io.bxmypk7w).

### Online database

diGLY peptide TMT ratios were normalized to the total proteome. For differential expression, these data were normalized to median in each sample to account for sample-to-sample variation. Differential expression was performed with limma (*66*) using logarithms of normalized peptide ratios. *P* values were corrected for multiple tests using Benjamini-Hochberg method. All analyses were done in R, and the code is available at https://github.com/bartongroup/MG_UbiMito (doi 10.5281/zenodo.5152822). An online interactive tool was created using Shiny framework, and it is publicly available at https://shiny.compbio.dundee.ac.uk/MitoNUb/ (RRID: SCR_021544). It allows for selection of peptides based on differential expression significance, fold change, presence or absence in MitoCarta, and/or total proteome. Selected peptides provide instantaneous Gene Ontology term and Reactome pathway enrichment results.

### Proteomics—Sample preparation for copy number analysis

Three biological replicates of C57Bl/6J mouse cortical neurons (technical duplicates each) were treated for 5 hours with 10 μM antimycin A and 1 μM oligomycin (AO) in DMSO at 37 °C in a incubator with 5% CO_2_. Neurons were lysed in lysis buffer containing tris·HCl (10 mM, pH 8.0), SDS (2%, w/v), sodium orthovanadate (1 mM), sodium glycerophosphate (10 mM), sodium fluoride (50 mM), sodium pyrophosphate (5 mM), protease inhibitor cocktail (Roche), and microcystin-LR (1 μg/ml). Lysates were boiled for 10 min at 95°C, followed by Bioruptor sonication for 10 min (30-s on and 30-s off, 10 cycles) at 4°C. Samples were spun at 20,000*g* in a centrifuge at 4°C for 20 min. Supernatants were collected, and protein concentration was determined by using the BCA kit (Pierce). Fifty micrograms of protein for each sample (*n* = 6 AO and *n* = 6 DMSO) and 300 μg of pooled cortical neurons for deep proteomic profile of cortical neurons were aliquoted and subjected to S-Trap assisted workflow as described in (*67*). Briefly, protein lysate was reduced with 10 mM TCEP and alkylated with 40 mM Iodoacetamide. Samples were then processed using S-Trap micro and mini columns. Samples were purified by washing with S-Trap wash buffer [100 mM Triethylammonium bicarbonate buffer (TEABC) (pH 7.2) in 90% methanol] four times. On-column Lys-C ± trypsin was added at 1:20 ratio and incubated at 47°C for 1.2 hours and then incubated overnight at room temperature. Peptides were eluted with sequential elution using 50 mM TEABC buffer, 0.15% formic acid (v/v), and 80% acetonitrile (ACN) in 0.15% formic acid (v/v); eluates were pooled and vacuum-dried. Pooled cortical neuron tryptic peptide digests were further fractionated using high-pH reversed-phase liquid chromatography (RPLC) fractionation, and 45 fractions were used for data-dependent acquisition (DDA) analysis. OA and DMSO treated samples were then dissolved in LC buffer [3% ACN in 0.1% formic acid (v/v)], and 2 μg of peptide was injected for DIA analysis. The main methods are described in a recent study (*67*).

### Proteomics—Copy number total proteomic analysis using DDA and DIA

Forty high-pH fractions were analyzed on Orbitrap Exploris 480 mass spectrometer coupled in line with Dionex 3000 RSLCnano liquid chromatography (LC) system. Each fraction of ~2 μg was dissolved in 15 μl of LC buffer [3% ACN in 0.1% formic acid (v/v)]. Sample was injected onto trap column (Acclaim PepMap 2 cm, 3 μm particle; C18#164569) and separated on a 50-cm analytical column at 300 nl/min (ES803; 50 cm, C18 2μ particle) and directly electrosprayed into the mass spectrometer using EASY nanoLC source. An 80-min nonlinear gradient was used to separate the peptides with a total run time of 100 min for each run. Data were acquired in a DDA mode by acquiring full MS at 60,000 resolution at a mass/charge ratio (*m*/*z*) of 200 and analyzed using Orbitrap mass analyzer. MS2 data were acquired at top speed for 2 s to acquire as many data-dependent scans by using 1.2-Da isolation window using quadrupole mass filter and fragmented using normalized 30% HCD (high-energy collision-induced dissociation); the MS fragment ion was measured at 15,000 resolution at *m*/z of 200 using Orbitrap mass analyzer. Automatic gain control (AGC) targets for MS1 was set at 300% and MS2 at 100% with a maximum ion-injection accumulation time at 25 and 80 ms, respectively. For the DIA analysis, 2 μg of peptide amount from each of the cortical neuron–treated samples (*n* = 6 AO and *n* = 6 DMSO) was acquired on an Orbitrap Exploris 480 mass spectrometer. Peptides were loaded on trap column and eluted on an analytical column by using a nonlinear gradient of 120 min and a total of 145-min run. MS1 data were acquired at 120,000 resolution at *m*/z of 200 and measured using Orbitrap mass analyzer. Variable DIA scheme was used by using a Quadrupole mass filter in the mass range of 400 to 1500 *m*/z. The DIA isolation window and instrument parameters were provided in table S1. Peptides were fragmented using a normalized steeped HCD collision energy (26, 28, and 30) and measured at 30,000 resolution at *m*/z of 200 using Orbitrap mass analyzer. AGC target for MS1 was set at 300% and for MS2 was set at 3000% with a maximum ion-injection accumulation time of 25 and 80 ms, respectively.

#### MS data analysis

DDA raw MS data were processed using Frag pipe software suite (version 15.0) using an in-built MS-Fragger search algorithm (*68*, *69*). Default closed search workflow was used and searched against Mouse UniProt database (March 2021, 34,350 entries). Precursor mass tolerance was set at −50 and ±50 ppm (parts per million), and fragment mass tolerance was set at 20 ppm. Trypsin as a strict protease by allowing a maximum missed cleavage of 2 and peptide length of 7 amino acid as minimum and 50 amino acid as maximum. Oxidation of Met and protein N-terminal acetylation were considerated as variable modifications. Carbamidomethylation of Cys as a fixed modification was used. MS1 quantification was performed using Ionquant algorithm by allowing match between runs. One percent false discovery rate (FDR) at peptide-spectrum match (PSM), peptide, and protein level was applied for the final output files. Protein group table was further processed using Perseus software suite to estimate copy numbers using histone proteomic ruler (*30*, *70*).

The DDA data were used to generate a spectral library using Spectronaut version 15 (Biognosys) pulsar search engine (*71*). This library was used for the library-based search for DIA data by using the default search parameters and enabling cross-run normalization. The search output protein group table was exported and processed using Perseus for further analysis. Student *t* test was carried out between AO and DMSO-treated cortical neurons by applying 1% permutation-based FDR for the identification of differentially regulated proteins (*70*). The main methods are described in a recent study (*67*).

### Proteomics—General sample preparation for TMT analysis

Protein extracts lysed in 8 M urea were subjected to disulfide bond reduction with 5 mM TCEP (room temperature, 10 min) and alkylation with 25 mM chloroacetamide (room temperature, 20 min). Methanol-chloroform precipitation was performed before protease digestion. Briefly, four parts of neat methanol were added to each sample and vortexed, one part chloroform was then added to the sample and vortexed, and last, three parts water was added to the sample and vortexed. The sample was centrifuged at 6000 rpm for 2 min at room temperature and subsequently washed twice with 100% methanol. Samples were resuspended in 100 mM 3-[4-(2-hydroxyethyl)piperazin-1-yl]propane-1-sulfonic acid (EPPS) pH 8.5 containing 0.1% RapiGest and digested at 37°C for 4 hours with LysC protease at a 200:1 protein-to-protease ratio. Trypsin was then added at a 100:1 protein-to-protease ratio, and the reaction was incubated for a further 6 hours at 37°C. Samples were acidified with 1% formic acid for 15 min and subjected to C18 solid-phase extraction (Sep-Pak, Waters). The Pierce Quantitative Colorimetric Peptide Assay (catalog no. 23275) was used to quantify the digest and to accurately aliquot the desired amount of peptides per sample needed for downstream application. A detailed protocol describing TMT proteomics has been reported (dx.doi.org/10.17504/protocols.io.buyunxww).

### Proteomics—Total proteomic analysis using TMT

The procedure is previously described in https://doi.org/10.1016/j.molcel.2019.11.013. Tandem mass tag labeling of each sample (100 μg of peptide input) was performed by adding 10 μl of the stock (20 ng/μl) of TMT reagent along with ACN to achieve a final ACN concentration of approximately 30% (v/v). Following incubation at room temperature for 1 hour, the reaction was quenched with hydroxylamine to a final concentration of 0.5% (v/v) for 15 min. The TMT-labeled samples were pooled together at a 1:1 ratio. The sample was vacuum-centrifuged to near dryness and subjected to C18 solid-phase extraction (50 mg; Sep-Pak, Waters).

Dried TMT-labeled sample was resuspended in 100 μl of 10 mM NH_4_HCO_3_ (pH 8.0) and fractionated using basic pH reversed-phase (BPRP) high-performance LC (HPLC) (*72*). Briefly, samples were offline-fractionated over a 90-min run, into 96 fractions by high-pH reverse-phase HPLC (Agilent, LC1260) through an Aeris peptide xb-c18 column (Phenomenex; 250 mm by 3.6 mm) with mobile phase A containing 5% ACN and 10 mM NH_4_HCO_3_ in LC-MS grade H_2_O and mobile phase B containing 90% ACN and 10 mM NH_4_HCO_3_ in LC-MS grade H_2_O (both pH 8.0). The 96 resulting fractions were then pooled in a noncontinuous manner into 24 fractions [as outlined in figure S5 of (*73*)], and 12 fractions (even numbers) were used for subsequent MS analysis. Fractions were vacuum-centrifuged to near dryness. Each consolidated fraction was desalted via StageTip, dried again via vacuum centrifugation, and reconstituted in 5% ACN and 1% formic acid for LC–tandem MS processing.

MS data were collected using an Orbitrap Fusion Lumos mass spectrometer (Thermo Fisher Scientific, San Jose, CA) coupled to a Proxeon EASY-nLC1200 LC pump (Thermo Fisher Scientific). Peptides were separated on a 100–μm–inner diameter microcapillary column packed in house with ∼35 cm of Accucore150 resin (2.6 μm, 150 Å; Thermo Fisher Scientific, San Jose, CA) with a gradient consisting of 5 to 21% (0 to 125 min), 21 to 28% (125 to 140 min) (ACN and 0.1% formic acid) over a total 150-min run at ∼500 nl/min. For analysis, we loaded one-tenth of each fraction onto the column. Each analysis used the Multi-Notch MS^3^-based TMT method (*74*) to reduce ion interference compared to MS^2^ quantification (*75*). The scan sequence began with an MS^1^ spectrum (Orbitrap analysis; resolution 120,000 at 200 Th; mass range of 400 to 1400 *m*/*z*; AGC target, 5 × 10^5^; maximum injection time, 50 ms). Precursors for MS^2^ analysis were selected using a Top10 method. MS^2^ analysis consisted of collision-induced dissociation [quadrupole ion trap analysis; Turbo scan rate; AGC, 2.0 × 10^4^; isolation window, 0.7 Th; normalized collision energy (NCE) 35; maximum injection time, 90 ms]. Monoisotopic peak assignment was used, and previously interrogated precursors were excluded using a dynamic window (150 s ± 7 ppm), and dependent scans were performed on a single charge state per precursor. Following acquisition of each MS^2^ spectrum, a synchronous-precursor-selection MS^3^ scan was collected on the top 10 most intense ions in the MS^2^ spectrum (*74*). MS^3^ precursors were fragmented by HCD and analyzed using the Orbitrap (NCE, 65; AGC, 3 × 10^5^; maximum injection time, 150 ms, resolution was 50,000 at 200 Th). A detailed protocol describing whole-cell proteomics and TMT-based proteomics has been reported (dx.doi.org/10.17504/protocols.io.bxa4pigw).

### Immunoprecipitation of diGLY-containing peptides

diGLY capture was performed largely as described (*25*). The diGLY monoclonal antibody (Cell Signaling Technology; D4A7 clone) (32 μg of antibody/1 mg of peptide) was coupled to Protein A Plus Ultralink resin (1:1 μl of slurry/μg of antibody) (Thermo Fisher Scientific) overnight at 4°C before its chemical cross-linking reaction. Dried peptides (1-mg starting material) were resuspended in 1.5 ml of ice-cold immuno affinity purification (IAP) buffer [50 mM Mops (pH 7.2), 10 mM sodium phosphate, and 50 mM NaCl] and centrifuged at maximum speed for 5 min at 4°C to remove any insoluble material. Supernatants (pH ∼ 7.2) were incubated with the antibody beads for 2 hours at 4°C with gentle end-over-end rotation. After centrifugation at 215*g* for 2 min, beads were washed three more times with ice-cold IAP buffer and twice with ice-cold PBS. The diGLY peptides were eluted twice with 0.15% trifluoroacetic acid, desalted using homemade StageTips, and dried via vacuum centrifugation, before TMT labeling. A detailed protocol describing the diGLY immunoprecipitation for TMT proteomics has been reported (dx.doi.org/10.17504/protocols.io.buyunxww).

### Proteomics—diGLY proteomic analysis using TMT

The procedure is previously described in https://doi.org/10.1016/j.molcel.2019.11.013. TMT-labeled diGLY peptides were fractionated according to the manufacturer’s instructions using high-pH reversed-phase peptide fractionation kit (Thermo Fisher Scientific) for a final six fractions and subjected to C18 StageTip desalting before MS analysis.

MS data were collected using an Orbitrap Fusion Lumos mass spectrometer (Thermo Fisher Scientific, San Jose, CA) coupled to a Proxeon EASY-nLC1200 LC pump (Thermo Fisher Scientific). Peptides were separated on a 100–μm–inner diameter microcapillary column packed in house with ∼35 cm of Accucore150 resin (2.6 μm, 150 Å; Thermo Fisher Scientific, San Jose, CA) with a gradient consisting of 3 to 26% (0 to 130 min) and 26 to 32% (130 to 140 min) (ACN and 0.1% formic acid) over a total 150-min run at ∼500 nl/min. For analysis, we loaded one-half of each fraction onto the column. Each analysis used the Multi-Notch MS^3^-based TMT method (*74*). The scan sequence began with an MS^1^ spectrum (Orbitrap analysis; resolution, 120,000 at 200 Th; mass range, 400 to 1250 *m*/*z*; AGC target, 1 × 10^6^; maximum injection time, 100 ms). Precursors for MS^2^ analysis were selected using a Top 4 s method. MS^2^ analysis consisted of collision-induced dissociation (quadrupole Orbitrap analysis; AGC, 1 × 10^5^; isolation window, 0.7 Th; NCE, 35; maximum injection time, 300 ms; resolution was 7500 at 200 Th). Monoisotopic peak assignment was used, and previously interrogated precursors were excluded using a dynamic window (120 s ± 7 ppm). As described previously, only precursors with a charge state between 3 and 6 were selected for downstream analysis (*25*). Following acquisition of each MS^2^ spectrum, a synchronous-precursor-selection MS^3^ scan was collected on the top 10 most intense ions in the MS^2^ spectrum (*74*). MS^3^ precursors were fragmented by HCD and analyzed using the Orbitrap (NCE, 65; AGC, 2 × 10^5^; maximum injection time, 500 ms; resolution was 50,000 at 200 Th). A detailed protocol describing the diGLY immunoprecipitation-based TMT proteomics has been reported (dx.doi.org/10.17504/protocols.io.buyunxww).

### Proteomics—Data analysis

Mass spectra were processed using a SEQUEST-based or Comet-based (2014.02 rev. 2) in-house software pipeline (*76*, *77*). Spectra were converted to mzXML using a modified version of ReAdW.exe. Database searching included all entries from the Mouse Reference Proteome (2017-05) UniProt database, as well as an in-house curated list of contaminants. This database was concatenated with one composed of all protein sequences in the reversed order. Searches were performed using a 20-ppm precursor ion tolerance for total protein level analysis. The product ion tolerance was set to 0.9 Da (0.03 Da for diGLY searches). These wide mass tolerance windows were chosen to maximize sensitivity in conjunction with SEQUEST searches and linear discriminant analysis (*77*, *78*). TMT tags on lysine residues and peptide N termini (+229.163 Da) and carbamidomethylation of cysteine residues (+57.021 Da) were set as static modifications, while oxidation of methionine residues (+15.995 Da) was set as a variable modification. For phosphorylation dataset search, phosphorylation (+79.966 Da) on serine or threonine and deamidation (+0.984 Da) on asparagine or glutamine were set as additional variable modifications. For diGLY dataset search, GlyGly modification (+114.0429 Da) was also set as a variable modification. PSMs were adjusted to a 1% FDR (*79*). PSM filtering was performed using a linear discriminant analysis, as described previously (*77*), while considering the following parameters: XCorr (or Comet Log Expect), ΔCn (or Diff Seq. Delta Log Expect), missed cleavages, peptide length, charge state, and precursor mass accuracy. For TMT-based reporter ion quantitation, we extracted the summed signal-to-noise ratio for each TMT channel and found the closest matching centroid to the expected mass of the TMT reporter ion (integration tolerance of 0.003 Da). For protein-level comparisons, PSMs were identified, quantified, and collapsed to a 1% peptide FDR and then collapsed further to a final protein-level FDR of 1%. Moreover, protein assembly was guided by principles of parsimony to produce the smallest set of proteins necessary to account for all observed peptides. Phosphorylation or ubiquitylation site localization was determined using the AScore algorithm (*78*). AScore is a probability-based approach for high-throughput protein phosphorylation site localization. Specifically, a threshold of 13 corresponded to 95% confidence in site localization. Proteins and phosphorylated or ubiquitylated peptides were quantified by summing reporter ion counts across all matching PSMs using in-house software, as described previously (*77*). PSMs with poor quality, MS^3^ spectra with isolation specificity less than 0.7, or with TMT reporter summed signal-to-noise ratio that were less than 150, or had no MS^3^ spectra were excluded from quantification (*80*).

Protein or peptide quantification values were exported for further analysis in Microsoft Excel, GraphPad Prism, and Perseus (*70*). For whole-proteome analysis, each reporter ion channel was summed across all quantified proteins and normalized assuming equal protein loading of all samples. For diGLY samples, the data were normalized to each individual protein abundance measured in parallel when available to correct for variation in protein abundance between treatments. Supplementary tables list all quantified proteins and associated TMT reporter ratio to control channels used for quantitative analysis.

Annotations for bona fide organellar protein markers were assembled using the proteins which had scored with confidence “very high” or “high” from the HeLa dataset previously published by Itzhak *et al.* (*81*). The following database containing mitochondrial protein was used: MitoCarta 3.0 (*34*). Detailed protocols describing the analysis for diGLY immunoprecipitation and TMT proteomics (dx.doi.org/10.17504/protocols.io.buyunxww) and whole-cell TMT proteomics (dx.doi.org/10.17504/protocols.io.bxa4pigw) have been reported.

### Mitochondrial UB and poly-UB capture and proteomics

Mitochondrially derived ubiquitylated proteins were purified using Halo-4×UBA^UBQLN1^ and Halo-5xUBA^DSK2^ as described (*15*, *82*). Briefly, whole-cell extracts (0.5 mg) or mitochondrial extracts (0.5 mg) that were lysed in lysis buffer containing 50 mM chloroacetamide were incubated at 4°C for 16 hours with 25 μl of Halo-4×UBA^UBQLN1^ beads (pack volume). Subsequently, the supernatant was incubated with 15 μl of Halo-5xUBA^DSK2^ for 2 hours to capture any possible residual mono-ubiquitylated proteins. Halo beads were combined and following four washes with lysis buffer containing 0.5 M NaCl and one final wash in 10 mM tris (pH 8.0); proteins were released from the Halo-UBA resin using 6 M guanidine HCL. Samples were subjected to TCA precipitation and digested overnight at 37°C with Lys-C and trypsin [in 100 mM tetraethylammonium bromide, 0.1% Rapigest (Waters Corporation), 10% (v/v) ACN]. Digests were acidified with an equal volume of 5% (v/v) formic acid to a pH of ∼2 for 30 min, dried down, resuspended in 5% (v/v) formic acid, and subjected to C18 StageTip (packed with Empore C18; 3 M Corporation) desalting. Samples were analyzed by LC/tandem MS for AQUA proteomics as described below. Ubiquitin capture for proteomics was performed largely as described previously (*15*, *82*).

### UB-AQUA proteomics

UB-AQUA was performed largely as described previously (*15*, *82*). A collection of 21 heavy-labeled reference peptides (*15*, *82*), each containing a single ^13^C/^15^N-labeled amino acid, was produced at Cell Signaling Technologies and quantified by amino acid analysis. UB-AQUA peptides from working stocks [in 5% (v/v) formic acid] were diluted into the digested sample [in 5% (v/v) formic acid] to be analyzed to an optimal final concentration predetermined for individual peptide such that each peptide’s intensity would range between 10^6^ and 10^8^. Samples and AQUA peptides were oxidized with 0.1% hydrogen peroxide for 30 min, subjected to C18 StageTip, and resuspended in 5% (v/v) formic acid. Replicate experiments were performed and analyzed sequentially by LC/MS on an Orbitrap Fusion Lumos instrument coupled to an Easy-nLC 1200 (Thermo Fisher Scientific) ultra-HPLC pump. Peptides were separated on a 100–μm–inner diameter microcapillary column packed in house with ∼35 cm of Accucore150 resin (2.6 μm, 150 Å; Thermo Fisher Scientific, San Jose, CA). The column was equilibrated with buffer A (3% ACN + 0.125% formic acid). Peptides were loaded onto the column in 100% buffer A. Separation and elution from the column were achieved using a 75-min 0 to 28% gradient of buffer B [100% (v/v) ACN + 0.125% formic acid]. The scan sequence began with FTMS^1^ spectra (resolution of 120,000; mass range, 300 to 1000 *m*/*z*; AGC target, 5 × 10^5^; maximum injection time of 100 ms). The second scan sequence consisted of a targeted-MS^2^ method that was MS^2^ precursors of interest was isolated using the quadrupole and analyzed in the Orbitrap (FTMS^2^) with a 0.7 Th isolation window, 30,000 resolution, 5 × 10^4^ AGC target, and a maximum injection time of 54 ms. MS2 precursors were fragmented by HCD at an NCE of 32%. LC-MS data analysis was performed using Skyline software (*83*) with manual validation of precursors and fragments. The results exported to Excel and GraphPad Prism for further analysis and plotting. Total UB was determined as the average of the total UB calculated for each individual locus, unless specified otherwise.

### Proteomics—Ubiquitylated site ranking analysis

In addition to TMT-based reporter ion quantitation, we also extracted the MS1 precursor abundance (intensity-based) for each diGLY peptide, a value indicative of the relative abundance of the peptide in the tryptic sample. Each MS1-based abundance measured should be a representation of the sum of all the respective TMT-labeled peptides combined. Therefore, for a rudimentary metric of site abundance across samples, we divided the total MS1 abundance for individual diGLY peptides by their respective TMT summed signal-to-noise ratio to each TMT channel.

### Mouse breeding and maintenance

The C57BL/6J mice were obtained from Charles River Laboratories (Kent-UK); the *PINK1* and *Parkin* KO mouse models used in this study were generated as previously described (*29*) housed in a specific pathogen–free facility in temperature-controlled rooms at 21°C, with 45 to 65% relative humidity and 12-hour light/12-hour dark cycles. Mice had ad libitum access to food and water and regularly monitored by the School of Life Science Animal Unit Staff. All animal studies and breeding were approved by the University of Dundee Ethical Review Committee and further subjected to approved study plans by the Named Veterinary Surgeon and Compliance Officer (N. Dennison). Experiments were conducted in accordance with the Animal Scientific Procedures Act (1986) and with the Directive 2010/63/EU of the European Parliament and of the Council on the protection of animals used for scientific purposes (2010, no. 63).
