# Supplementary material for: Global ubiquitylation analysis of mitochondria in primary neurons identifies endogenous Parkin targets following activation of PINK1
Source: Sci Adv. 2021 Nov 12;7(46):eabj0722. doi: 10.1126/sciadv.abj0722 (PMC8589319; doi:10.1126/sciadv.abj0722)
Supplement: Supplementary file 1 — Supplementary Materials and Methods Figs. S1 to S16 Legends for tables S1 to S5 [file sciadv.abj0722_sm.pdf]

Science Advances
AAAS

Supplementary Materials for

Global ubiquitylation analysis of mitochondria in primary neurons identifies endogenous Parkin targets following activation of PINK1

Odetta Antico, Alban Ordureau, Michael Stevens, Francois Singh, Raja S. Nirujogi, Marek Gierlinski, Erica Barini, Mollie L. Rickwood, Alan Prescott, Rachel Toth, Ian G. Ganley, J. Wade Harper*, Miratul M. K. Muqit*

*Corresponding author. Email: wade_harper@hms.harvard.edu (J.W.H.); m.muqit@dundee.ac.uk (M.M.K.M.)

Published 12 November 2021, *Sci. Adv.* **7**, eabj0722 (2021)
DOI: 10.1126/sciadv.abj0722

The PDF file includes:

Supplementary Materials and Methods
Figs. S1 to S16
Legends for tables S1 to S5

Other Supplementary Material for this manuscript includes the following:

Tables S1 to S5

Materials and Methods

List of the key resources

REAGENT	COMPANY	CAT. NUMBER	RRID/ URL
**Antibodies**			
**Anti-CPT1α**	Abcam	ab128568	RRID:AB_11141632
**Anti-CISD1**	Proteintech	16006-1-AP	RRID:AB_2080268
**Anti-CISD1**	Cell Signaling Technology	83775	RRID:AB_2800031
**Anti-ACSL1**	Cell Signaling Technology	4047	RRID:AB_2222411
**Anti- ACSL6**	Sigma-Aldrich	HPA040470	RRID:AB_10794033
**Anti-PARKIN**	Santa-Cruz	sc-32282	RRID:AB_628104
**Anti-Ubiquitin**	BioLegend	646302	RRID:AB_1659269
**Anti-pS65-Ubiquitin**	MRC PPU Products & Reagents	–	https://mrcppureagents.dundee.ac.uk/reagents-antibodies/
**Anti-pS65-Ubiquitin**	Cell Signaling Technology	62802	RRID:AB_2799632
**Anti-pS65-Parkin**	Michael J. Fox Foundation for Research	–	–
**Anti-PINK1 (235–580aa)**	MRC PPU Products & Reagents	S774C (DU17570)	https://mrcppureagents.dundee.ac.uk/reagents-view-antibodies/589008
**Anti- PINK1 (175–250 aa)**	MRC PPU Products & Reagents	S086D (DU34559)	https://mrcppureagents.dundee.ac.uk/reagents-view-antibodies/589183
**Anti-AGPAT5**	Abcam	ab82783	RRID:AB_1859760
**Anti-ATAD1**	UC Davis/NIH NeuroMab Facility	N125/10	RRID:AB_2877348
**Anti-ABCD3**	Aviva Systems Biology	ARP43641_P050	RRID:AB_10642411
**Anti-MFN1**	Abcam	ab126575	RRID:AB_11141234
**Anti-MFN2**	Proteintech	12186-1-AP	RRID:AB_2266320
**Anti-MFN2**	Abcam	ab124773	RRID:AB_10999860
**Anti-CYB5R3**	Sigma-Aldrich	SAB2501315	RRID:AB_10628632
**Anti-CYB5B**	Novus	NBP188039	RRID:AB_11029659
**Anti-MARC2**	Sigma-Aldrich	HPA015085	RRID:AB_1848780
**Anti-MAO-B**	Abcam	ab137778	
**Anti-MAO-A**	Proteintech	10539-1-AP	RRID:AB_2137251
**Anti-RHOT2**	Proteintech	11237-1-AP	RRID:AB_2179539
**Anti-TOMM70**	Aviva Systems Biology	OAAN01138	–
**Anti- DCAKD**	Aviva Systems Biology	OAAB13160	–
**Anti-FAM213A**	Novus	NBP2-48573	–
**Anti-GK**	Abcam	ab126599	RRID:AB_11129767
**Anti-TDRKH**	Proteintech	13528-1-AP	RRID:AB_2303299
**Anti-HK1**	Thermo Fisher Scientific	MA5-15680	RRID:AB_10979325
**Anti-HK1**	Cell Signaling Technology	2024	RRID:AB_2116996

**Anti-RAB5c**	MyBiosource	MBS448070	–
**Anti-ARHGAP33**	Sigma-Aldrich	HPA030117	RRID:AB_10599306
**Anti-CaMKII alpha**	Thermo Fisher Scientific	13-7300	RRID:AB_2533032
**Anti-CaMKII beta**	Thermo Fisher Scientific	13-9800	RRID:AB_2533045
**Anti-CDK16**	Biorbyt	orb353214	–
**Anti-DCAMKL2**	Abcam	ab106639	RRID:AB_10887397
**Anti-Nav1.7**	Novus	NBP2-12904	–
**Anti-PKC Gamma**	Proteintech	66429-1-Ig	RRID:AB_2881800
**Anti-RIMS4**	Proteintech	20091-1-AP	RRID:AB_10667403
**Anti-RUFY3**	Aviva Systems Biology	ARP34340_P050	RRID:AB_2184076
**Anti-SH3BP4**	Novus	NBP1-77031	RRID:AB_11033475
**Anti-SNX3**	Sigma-Aldrich	SAB1404616	RRID:AB_10759530
**Anti-CAD**	Novus	NB100-61613	RRID:AB_959373
**Anti- FBXO41**	Proteintech	24519-1-AP	RRID:AB_2879586
**Anti-CNN3**	Sigma-Aldrich	SAB140886	–
**Anti-MAPRE2**	Proteintech	10364-1-AP	RRID:AB_2141649
**Anti-p23**	Thermo Fisher Scientific	MA3-414	RRID:AB_2175197
**Anti-HSDL1**	Proteintech	16988-1-AP	RRID:AB_2120235
**Anti-GAPDH**	Santa Cruz Biotechnology	sc-32233	RRID:AB_627679
**Anti-VPS35**	Abcam	ab157220	RRID:AB_2636885
**Anti-OPA1**	Cell Signaling Technology	80471S	RRID:AB_2734117
**Anti-Rab8A**	Cell Signaling Technology	6975	RRID:AB_10827742
**Anti-Rab8A (phospho S111) [MJF-R27-30]**	Abcam	ab267492	–
**Anti-VDAC**	Cell Signaling Technology	4661	RRID:AB_10557420
**Anti_MAP2**	Sigma-Aldrich	M2320	RRID:AB_609904
**Anti-GFAP**	Abcam	ab7260	RRID:AB_305808
**Anti-Rabbit IgG (H+L), HRP Conjugate**	Thermo Fisher Scientific	31460	RRID:AB_228341
**Anti-Mouse IgG (H+L), HRP Conjugate**	Thermo Fisher Scientific	31450	RRID:AB_228427
**Donkey Anti-Mouse Alexa-Fluor-488**	Thermo Fisher Scientific	A32766	RRID:AB_2762823
**Donkey Anti-Rabbit Alexa-Fluor-594**	Thermo Fisher Scientific	A32754	RRID:AB_2762827
**PTMScan Ubiquitin Remnant Motif (K-ε-GG) (D4A7)**	Cell Signaling Technology	Custom order	–

**Chemicals**			
**Hoechst33342**	Thermo Fisher Scientific	62249	
**Oligomycin A**	Sigma-Aldrich	75351	
**Antimycin A**	Sigma-Aldrich	A8674	
**Phosphatase Inhibitor Cocktail 3**	Sigma-Aldrich	P0044	
**Phosphatase Inhibitor Cocktail 2**	Sigma-Aldrich	P5726	
**cOmplete™ EDTA-free Protease Inhibitor Cocktail**	Merck	11873580001	
**TCEP**	Gold Biotechnology	TCEP2	
**Hydrogen Peroxide**	Sigma-Aldrich	H1009	
**Formic Acid**	Sigma-Aldrich	94318	
**Trysin**	Promega	V511C	
**Lys-C**	Wako Chemicals	129-02541	
**EPPS**	Sigma-Aldrich	E9502	
**2-Chloroacetamide**	Sigma-Aldrich	C0267	
**PR-619**	Selleck Chem	S7130	
**Protein A Plus Ultralink resin**	Thermo-Fisher Scientific	53142	
**Halo-Link Resin**	Promega	G1913	
**Recombinant Proteins**			
**His-Halo-TUBE**	MRC PPU Products & Reagents	DU23799	
**His-Halo-multiDSK**	MRC PPU Products & Reagents	DU55458	
**His-Halo-multiDSK mutant**	MRC PPU Products & Reagents	DU55636	
**Critical Commercial Assays**			
**Pierce™ High pH Reversed-Phase Peptide Fractionation Kit**	Thermo Fisher Scientific	84868	
**High-Select™ Fe-NTA Phosphopeptide Enrichment Kit**	Thermo Fisher Scientific	A32992	
**Tandem Mass Tags**	Thermo Fisher Scientific	90406	
**Quantitative Colorimetric Peptide Assay**	Thermo Fisher Scientific	23275	
**Bio-Rad Protein Assay Dye Reagent Concentrate**	Bio-Rad	5000006	
**Experimental Models**			
**Mouse model:C57BL/6J**	Charles River Laboratories Kent-UK	N/A	
**Mouse model: *Pink1* KO mice**	Dr. L. Miguel Martins, Leicester UK; (Mice generated by Lexicon Pharmaceuticals, Inc.)	N/A	
**Mouse model: *Parkin* KO mice**	Dr. Olga Corti, Paris FRANCE	N/A	
**Mouse model: *VPS35 D620N* mice**	Dr. M.J.Farrer, The University of British Columbia; (Mice generated by The Jackson laboratory and Ozgene)	N/A	
**Recombinant DNA**			
**pET15 His-SUMO-Parkin**	MRC PPU Products & Reagents	DU42315	https://mrcppureagents.dundee.ac.uk/search/site/42315
**pMal4C MBP-tcPink1 (1-570)**	MRC PPU Products & Reagents	DU34701	https://mrcppureagents.dundee.ac.uk/reagents-view-cdna-clones/567800

**pMal4C MBP-tcPink1 (1-570) D359A**	MRC PPU Products & Reagents	DU34832	https://mrcppureagents.dundee.ac.uk/reagents-view-cdna-clones/567925
**pMEX3Cb MBP-3C-CamK2A**	MRC PPU Products & Reagents	DU61023	https://mrcppureagents.dundee.ac.uk/reagents-view-cdna-clones/694277
**pMEX3Cb MBP-3C-CamK2B**	MRC PPU Products & Reagents	DU61028	https://mrcppureagents.dundee.ac.uk/reagents-view-cdna-clones/694279
**pGEX6P1 GST-3C-MAOA**	MRC PPU Products & Reagents	DU63463	https://mrcppureagents.dundee.ac.uk/reagents-view-cdna-clones/694772
**pGEX6P1 GST-3C-MAOB**	MRC PPU Products & Reagents	DU63466	https://mrcppureagents.dundee.ac.uk/reagents-view-cdna-clones/694775
**pGEX6P1 GST-3C-Fam213A**	MRC PPU Products & Reagents	DU63451	https://mrcppureagents.dundee.ac.uk/reagents-view-cdna-clones/694760
**pGEX6P1 GST-3C-Miro1 (1-592)**	MRC PPU Products & Reagents	DU43034	https://mrcppureagents.dundee.ac.uk/reagents-view-cdna-clones/581618
**pET15 His-SUMO-SNX3**	MRC PPU Products & Reagents	DU61034	https://mrcppureagents.dundee.ac.uk/reagents-view-cdna-clones/694281
**pET15 His-SUMO-ACSL1 (T46-end)**	MRC PPU Products & Reagents	DU67025	https://mrcppureagents.dundee.ac.uk/reagents-view-cdna-clones/698447
**pET15 His-SUMO-CPT1a (R145-K773-end)**	MRC PPU Products & Reagents	DU63833	https://mrcppureagents.dundee.ac.uk/reagents-view-cdna-clones/697469
**pET24-Ubiquitin (silent a220c)**	MRC PPU Products & Reagents	DU20027	https://mrcppureagents.dundee.ac.uk/reagents-view-cdna-clones/576373
**pFastBac HTb Ube1**	MRC PPU Products & Reagents	DU32888	https://mrcppureagents.dundee.ac.uk/reagents-view-cdna-clones/560236
**pGEX6P-1-UBCH7**	MRC PPU Products & Reagents	DU3772	https://mrcppureagents.dundee.ac.uk/reagents-view-cdna-clones/556142
**B58 mod -6His-SUMO-parkin**	MRC PPU Products & Reagents	DU42598	https://mrcppureagents.dundee.ac.uk/reagents-view-cdna-clones/591470
**Software and Algorithms**			
**PyMOL**	The PyMOL Molecular Graphics System, v.1.8.6.0, Schrodinger, LLC		https://pymol.org
**Prism**	GraphPad, v7		https://www.graphpad.com/scientific-software/prism/
**In-house mass spectrometry data analysis software**	Huttlin et al Cell. (2010) 143:1174-89.		N/A
**SEQUEST**	Eng et al., 1994		N/A
**Comet**	Eng et al., 2012		http://comet-ms.sourceforge.net/
**Perseus**	Tyanova et al., Nat Methods. (2016) 13:731-40.		http://www.perseus-framework.org
**Other**			
**Orbitrap Fusion Lumos Mass Spectrometer**	ThermoFisher Scientific	IQLAAEGAAPFADBMBHQ	
**Easy-nLC 1200**	ThermoFisher Scientific	LC140	
**Aeris™ 2.6 μm PEPTIDE XB-C18 100 Å 250 x 4.6 mm**	Phenomenex	00G-4505-E0	

**Sep-Pak tC18 1cc Vac Cartridge, 50 mg**	Waters	WAT054960	
**SOLA HRP SPE Cartridge, 10 mg**	Thermo Fisher Scientific	60109-001	
**Empore™ SPE Disks C18**	3M Bioanalytical Technologies	2215	
**Bio-Rad Protein Assay Dye Reagent Concentrate**	Bio-Rad	5000006	

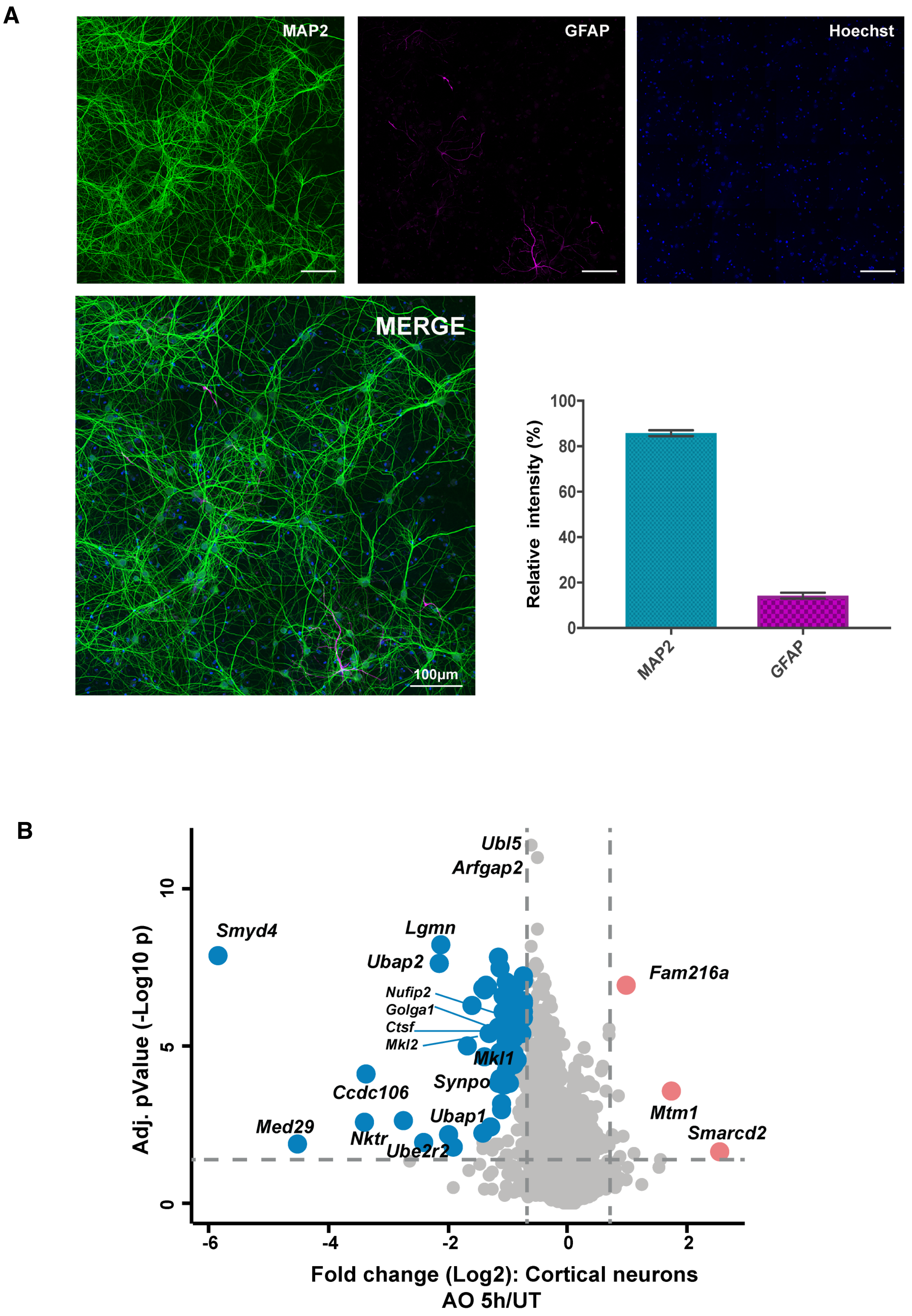

A
MAP2
GFAP
Hoechst
MERGE
100μm
Relative intensity (%)
100
80
60
40
20
0
MAP2
GFAP
B
Adj. pValue (-Log10 p)
Fold change (Log2): Cortical neurons
AO 5h/UT
Ubl5
Arfgap2
Smyd4
Lgmn
Ubap2
Fam216a
Nufip2
Golga1
Ctsf
Mkl2
Mkl1
Synpo
Ccdc106
Med29
Nktr
Ubap1
Ube2r2
Mtm1
Smarcd2
10
5
0
-6
-4
-2
0
2

Fig. S1. Characterisation of mouse cortical neuronal culture.

A. Immunocytochemistry in C57BL/6J mouse cortical neuronal cultures and relative quantification. Cell type distribution was visualized by immunofluorescence staining for the astrocytic protein GFAP (magenta), the neuronal protein MAP2 (green), and nuclei were stained with Hoechst (blue). Scale bar, 100 µm. 2 independent experiments. Histogram graph represents the percent area of signal by dividing the number of magenta or green pixels by the total number of magenta and green, multiplied by 100. Data are shown as mean ± SEM ($MAP2^+$/total 85.76% ± 1.28; $GFAP^+$/total 14.27% ± 1.28).

B. Volcano plot: DIA proteomic analysis of AO and DMSO treated (n=6) cortical neurons depicted as volcano plot showing differentially regulated proteins. T-test analysis was carried out using Perseus software and the permutation-based 1% FDR was applied to denote the significance.

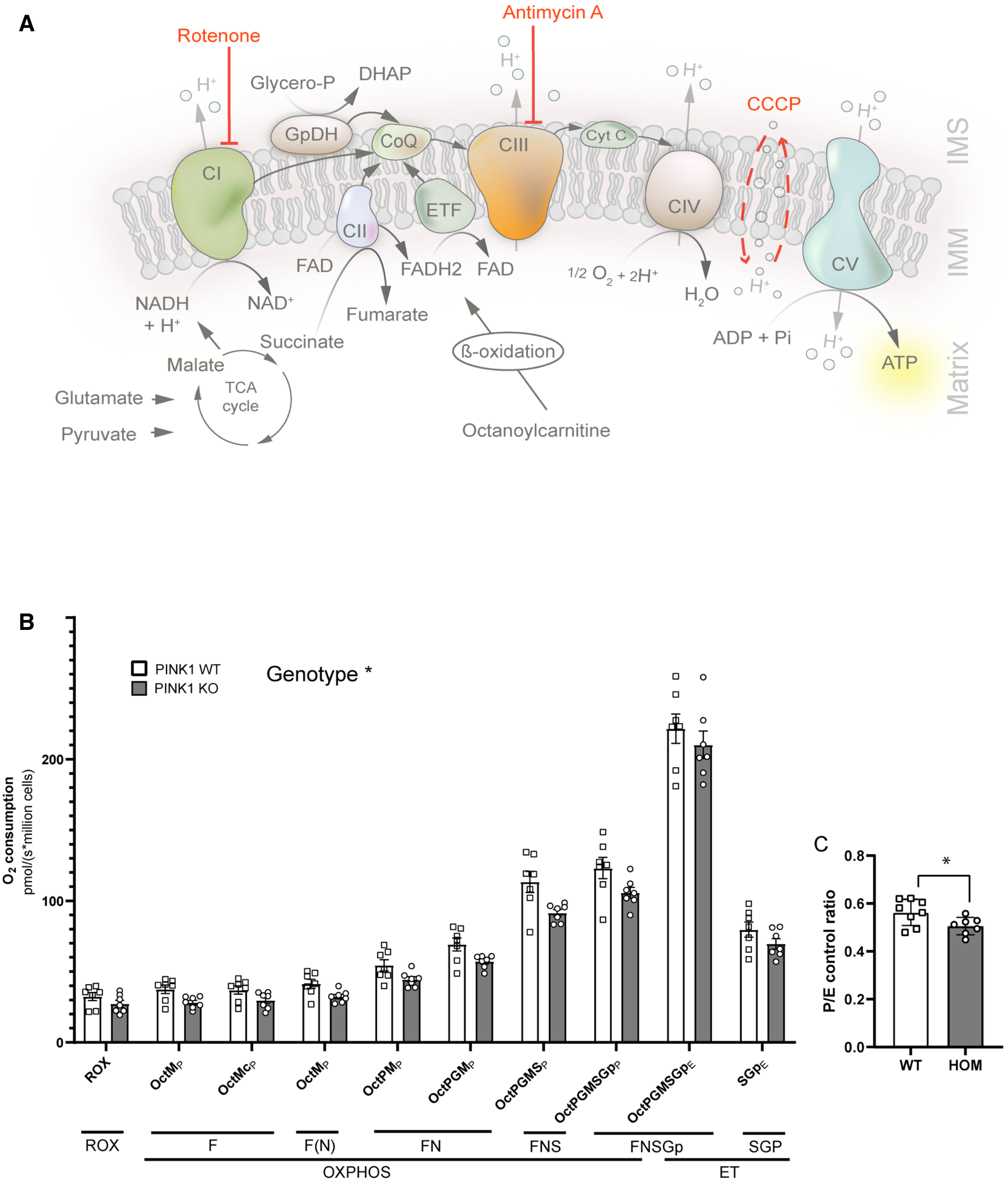

A
Rotenone
Antimycin A
CCCP
H+
Glycero-P
DHAP
GpDH
CoQ
CI
CII
ETF
CIII
Cyt C
CIV
CV
IMS
IMM
Matrix
FAD
FADH2
FAD
1/2 O2 + 2H+
H2O
NADH
+ H+
NAD+
Fumarate
Succinate
Malate
TCA
cycle
Glutamate
Pyruvate
ß-oxidation
Octanoylcarnitine
ADP + Pi
ATP
B
PINK1 WT
PINK1 KO
Genotype *
O2 consumption
pmol/(s*million cells)
0
100
200
ROX
OctMP
OctMcP
OctMP
OctPMP
OctPGMP
OctPGMSP
OctPGMSGpP
OctPGMSGpE
SGpE
ROX
F
F(N)
FN
FNS
FNSGp
SGP
OXPHOS
ET
C
P/E control ratio
0.0
0.2
0.4
0.6
0.8
*
WT
HOM

Fig. S2. Mitochondrial respiration in primary mouse cortical neurons.

A. Schematic representation of the mitochondrial electron transport chain located within the inner mitochondrial membrane (IMM). Electron transfer is coupled to the transfer of protons (H^+) from the matrix to the intermembrane space (IMS), resulting in the creation of a proton gradient. This gradient is coupled to the ATP synthesis by complex V. Respiratory capacity of mitochondria is evaluated by coupled oxidative phosphorylation (OXPHOS) of fuel substrates (malate, pyruvate, glutamate, succinate, Octanoylcarnitine, and Glycerophosphate) and by uncoupled respiration induced by titration of an established uncoupler (CCCP) to collapse the proton gradient across the IMM and measure electron-transfer-pathway capacity (ET). **B.** High-resolution respirometry of PINK1 KO and WT primary neuron cultures. **C.** P/E control ratio of B. Data is displayed as mean +/- SEM. *$p<0.05$. (Glycero-P: Glycerophosphate, DHAP: Dihydroxyaceton phosphate).

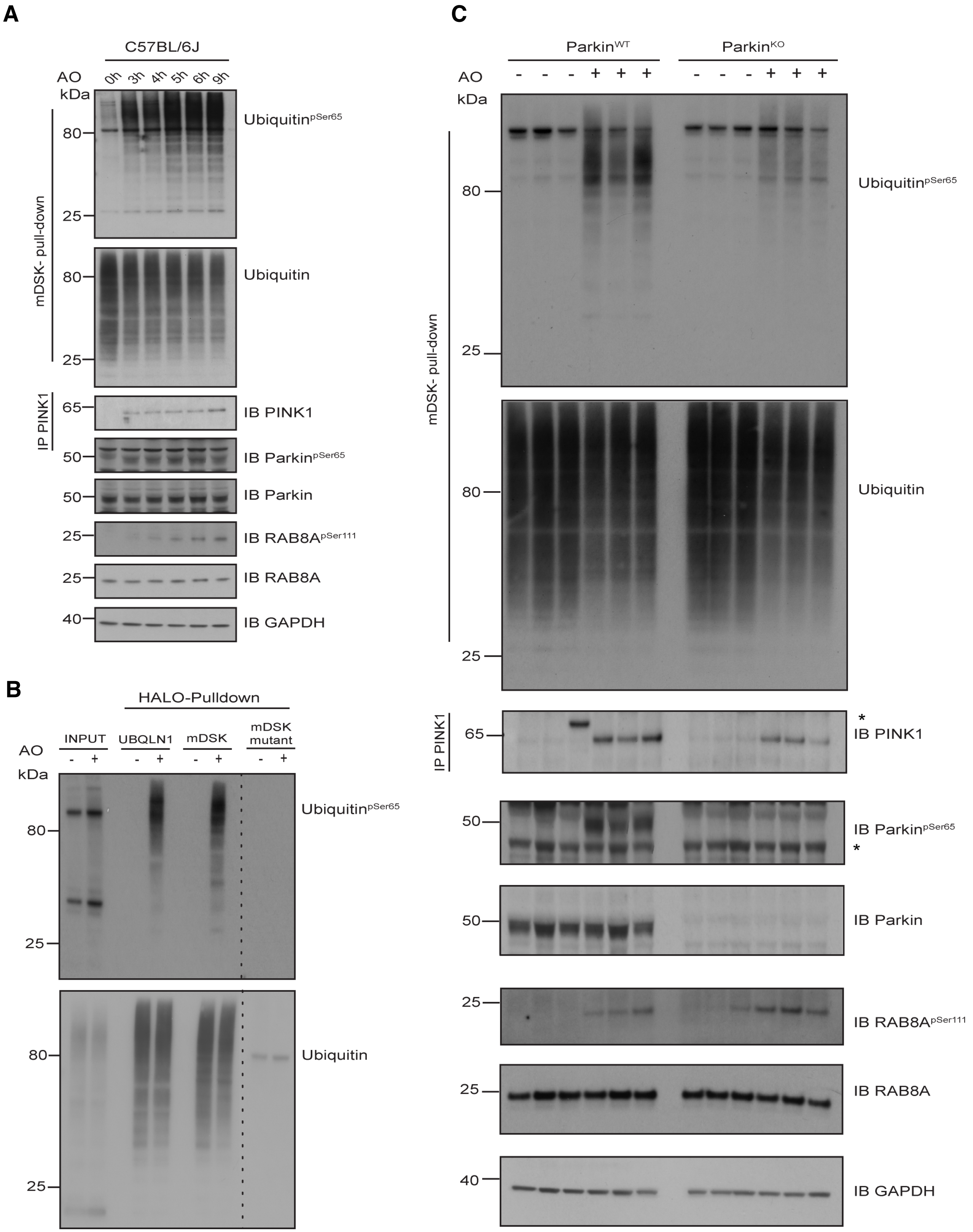
A
C57BL/6J
AO
0h
3h
4h
5h
6h
9h
kDa
80
25
mDSK- pull-down
Ubiquitin^{pSer65}
Ubiquitin
IP PINK1
65
IB PINK1
50
IB Parkin^{pSer65}
IB Parkin
IB RAB8A^{pSer111}
IB RAB8A
40
IB GAPDH
B
HALO-Pulldown
INPUT
UBQLN1
mDSK
mDSK mutant
- +
C
Parkin^{WT}
Parkin^{KO}
- - - + + +
*

Fig. S3. Characterisation of PINK1 signalling in mouse cortical neurons.

A. Time-course analysis of phospho-Ser65 ubiquitin levels in C57BL/6J mouse cortical neurons upon AO stimulation. Membrane lysates were subjected to Halo-multiDSK (mDSK) pull-down assay and immunoblotting with anti-phospho-Ser65 ubiquitin and anti-ubiquitin antibodies. PINK1 stabilisation was detected by immunoprecipitation-immunoblot. Immunoblot analysis with anti-Parkin, anti-phospho-Ser65 Parkin, anti-phospho-Ser111 RAB8A, anti-RAB8A and anti-GAPDH antibodies. **B.** Comparison of phospho-Ser65 ubiquitin detection using Halo-TUBE and/or Halo-multiDSK (mDSK) proteins enrichment. Mutant non-binding form of Halo-multiDSK (mDSK) used as negative control for the ubiquitin pull-down assay. Immunoblotting with anti-ubiquitin antibody was used as loading control. **C.** Immunoblots showing comparative analysis of phospho-Ser65 Ubiquitin levels in primary cortical neuron cultures from wild-type and Parkin knockout (KO) mice. Membrane lysate were enriched for ubiquitin by incubating with Halo-multiDSK (mDSK). Enriched lysates were subjected to immunoblotting with anti-phospho-Ser65 ubiquitin and anti-ubiquitin antibodies. IP-immunoblot showed PINK1 protein stabilisation after mitochondrial depolarisation. Membrane lysate were also subjected to SDS-PAGE and immunoblot analysis with anti-Parkin, anti-phospho-Ser65 Parkin, anti-phospho-Ser111 RAB8A, anti-RAB8A and anti-GAPDH antibodies. * Non-specific band.

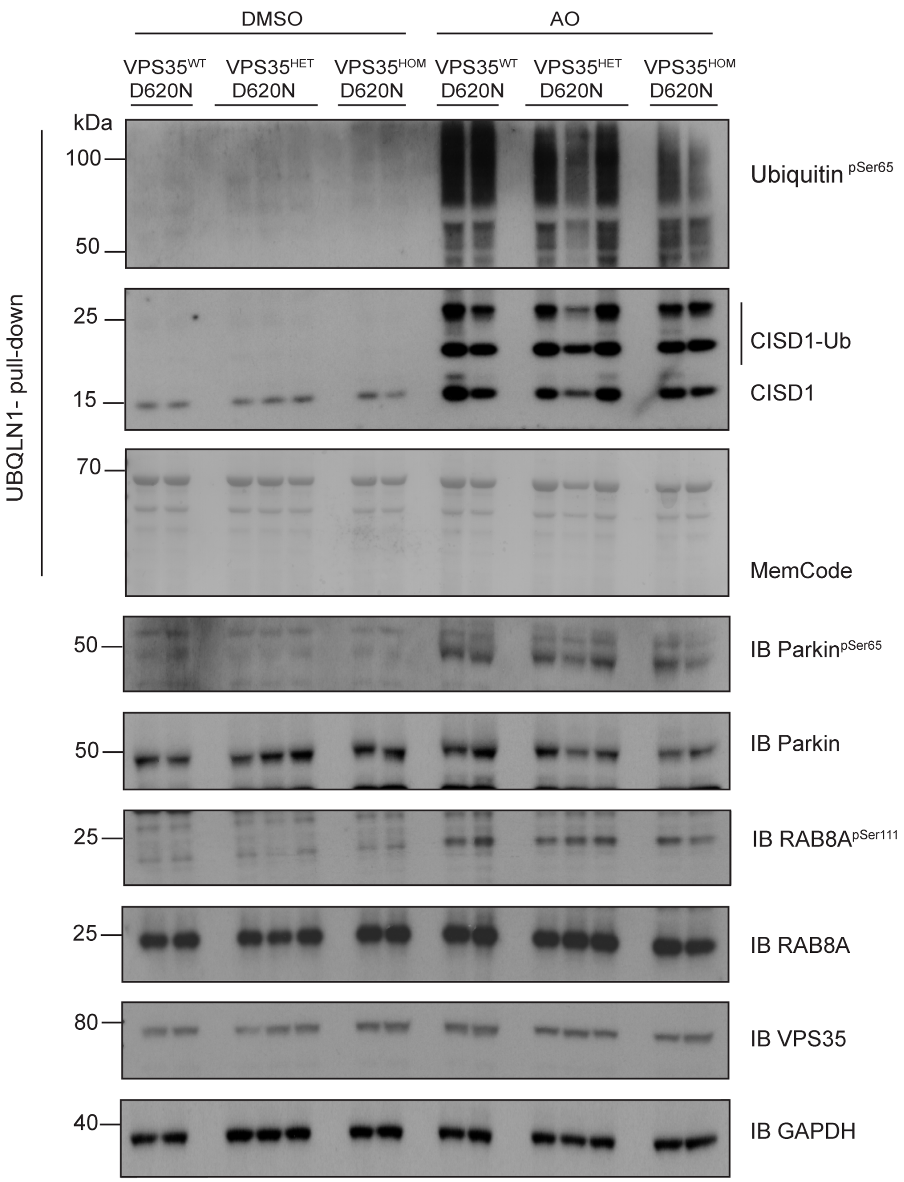

Fig. S4. Parkin activation is not affected in VPS35 D620N neurons.

Phospho-Ser65 ubiquitin levels were detected in *VPS35* D620N mouse cortical neurons after 5 hours of AO stimulation. Whole cell lysates were enriched for ubiquitin by incubating with Halo-UBQLN1. Enriched lysates were subjected to immunoblotting with anti-phospho-Ser65 ubiquitin and anti-CISD1 antibodies. MemCode was used as loading control. Whole cell lysates were also subjected to SDS-PAGE and immunoblot analysis with anti-Parkin, anti-phospho-Ser65 Parkin, anti-phospho-Ser111 RAB8A, anti-RAB8A, ant-VPS35 and anti-GAPDH antibodies.

A

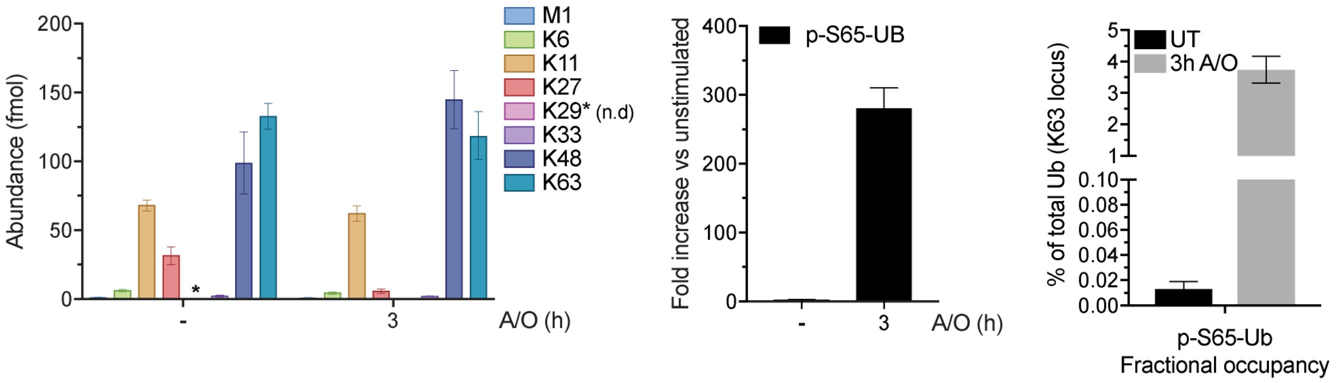

B

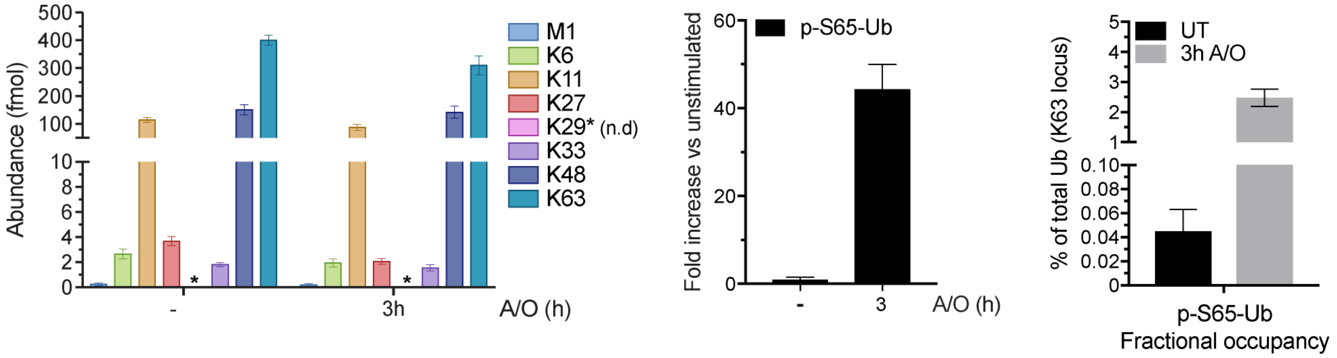

Fig. S5. Ubiquitin Linkage analysis in DIV 16 neurons.

A. Cortical neurons were depolarized with AO (3h) and extracts subject to Ub-AQUA proteomics. Abundance (fmol) or fold increase for individual Ub chain linkage types or pS65-Ub phosphorylation are plotted. **B.** Same as (A) but extracts were first subjected to sequential Halo-4×UBA^{UBQLN1}- and Halo-5xUBA^{DSK2}- coupled resin enrichment (see Methods) to isolate ubiquitin and ubiquitylated proteins prior to Ub-AQUA analysis. Error bars represent SEM, n=4. n.d., not determined.

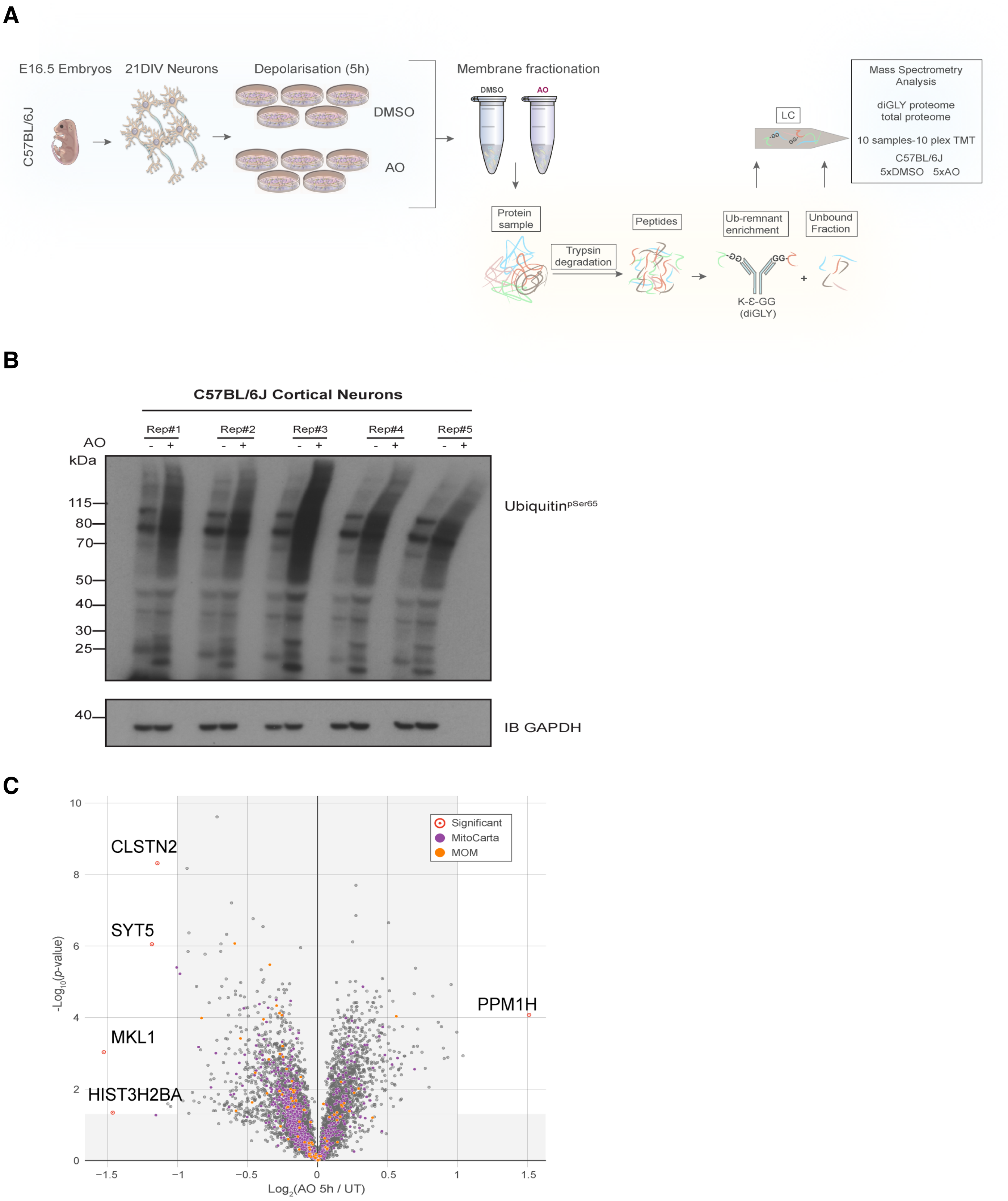

A
E16.5 Embryos
21DIV Neurons
Depolarisation (5h)
C57BL/6J
DMSO
AO
Membrane fractionation
DMSO
AO
Protein sample
Trypsin degradation
Peptides
Ub-remnant enrichment
Unbound Fraction
K-ε-GG (diGLY)
LC
Mass Spectrometry Analysis
diGLY proteome
total proteome
10 samples-10 plex TMT
C57BL/6J
5xDMSO 5xAO
B
C57BL/6J Cortical Neurons
Rep#1
Rep#2
Rep#3
Rep#4
Rep#5
AO
kDa
115
80
70
50
40
30
25
Ubiquitin pSer65
40
IB GAPDH
C
Significant
MitoCarta
MOM
CLSTN2
SYT5
MKL1
HIST3H2BA
PPM1H
-Log10(p-value)
Log2(AO 5h / UT)

Fig. S6. Proteomic and Biochemical analysis of mouse neurons.

A. Schemata of diGly affinity capture in C57BL/6J primary cortical neurons stimulated with AO for 5 h. **B.** 5 replicates of E16.5 derived C57Bl/6J primary cortical neurons were stimulated with 10 µM Antimycin A and 1 µM Oligomycin (AO) for 5 h. DMSO as vehicle. Phospho-Ser65 ubiquitin was detected in membrane enriched lysates. GAPDH was used as a loading control. **C.** Total protein abundance in neurons after treatment with AO. C56BL/6J primary cortical neurons were depolarized with AO (5 h) and membrane enriched lysates were subjected to quantitative proteomics. Fold increase for individual protein is shown in the Volcano plot. The x-axis specifies the fold-changes (FC) and the y-axis specifies the negative logarithm to the base 10 of the t-test p-values (Welch's t-test (S0=2), corrected for multiple comparison by permutation-based FDR (1%)). Proteins associated with mitochondria (MitoCarta 3.0) or Mitochondrial outer membrane localization are indicated.

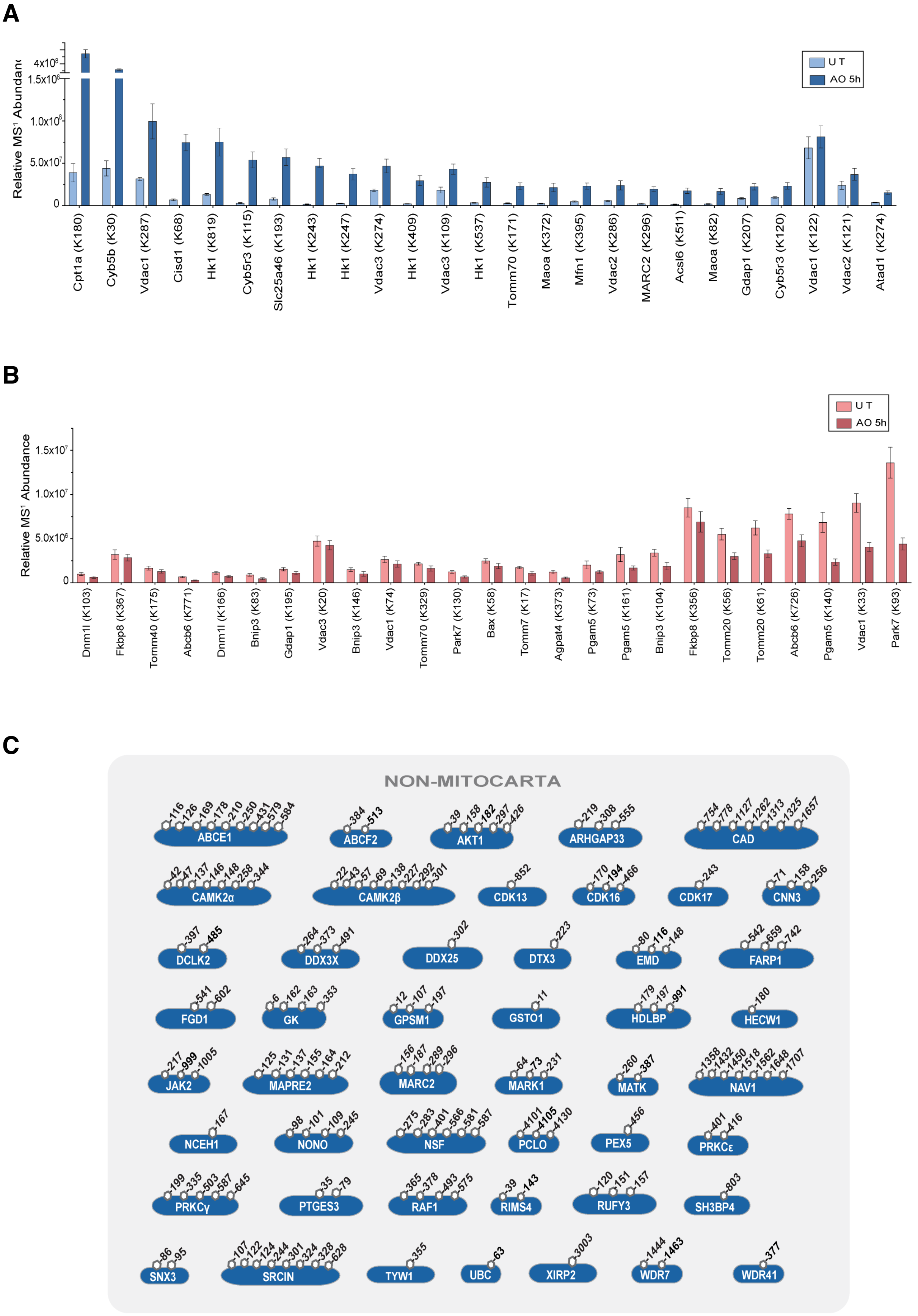
A
Relative MS¹ Abundance
4x10⁸
1.5x10⁸
1.0x10⁸
5.0x10⁷
0
U T
AO 5h
Cpt1a (K180)
Cyb5b (K30)
Vdac1 (K287)
Cisd1 (K68)
Hk1 (K819)
Cyb5r3 (K115)
Slc25a46 (K193)
Hk1 (K243)
Hk1 (K247)
Vdac3 (K274)
Hk1 (K409)
Vdac3 (K109)
Hk1 (K537)
Tomm70 (K171)
Maoa (K372)
Mfn1 (K395)
Vdac2 (K286)
MARC2 (K296)
Acsl6 (K511)
Maoa (K82)
Gdap1 (K207)
Cyb5r3 (K120)
Vdac1 (K122)
Vdac2 (K121)
Atad1 (K274)
B
Relative MS¹ Abundance
1.5x10⁷
1.0x10⁷
5.0x10⁶
0
U T
AO 5h
Dnm1l (K103)
Fkbp8 (K367)
Tomm40 (K175)
Abcb6 (K771)
Dnm1l (K166)
Bnip3 (K83)
Gdap1 (K195)
Vdac3 (K20)
Bnip3 (K146)
Vdac1 (K74)
Tomm70 (K329)
Park7 (K130)
Bax (K58)
Tomm7 (K17)
Agpat4 (K373)
Pgam5 (K73)
Pgam5 (K161)
Bnip3 (K104)
Fkbp8 (K356)
Tomm20 (K56)
Tomm20 (K61)
Abcb6 (K726)
Pgam5 (K140)
Vdac1 (K33)
Park7 (K93)
C
NON-MITOCARTA
ABCE1 116 126 169 178 210 250 431 579 584
ABCF2 384 513
AKT1 39 158 182 297 426
ARHGAP33 219 308 555
CAD 754 778 1127 1262 1313 1325 1657
CAMK2α 42 47 137 146 148 258 344
CAMK2β 22 43 57 69 138 227 292 301
CDK13 852
CDK16 170 194 466
CDK17 243
CNN3 71 158 256
DCLK2 397 485
DDX3X 264 373 491
DDX25 302
DTX3 223
EMD 80 116 148
FARP1 542 659 742
FGD1 541 602
GK 6 162 163 353
GPSM1 12 107 197
GSTO1 11
HDLBP 179 197 991
HECW1 180
JAK2 217 999 1005
MAPRE2 125 131 137 155 164 212
MARC2 156 187 289 296
MARK1 64 73 231
MATK 260 387
NAV1 1358 1432 1450 1518 1562 1648 1707
NCEH1 167
NONO 98 101 109 245
NSF 275 283 401 566 581 587
PCLO 4101 4105 4130
PEX5 456
PRKCε 401 416
PRKCγ 199 335 503 587 645
PTGES3 35 79
RAF1 365 378 493 575
RIMS4 39 143
RUFY3 120 151 157
SH3BP4 803
SNX3 86 95
SRCIN 107 122 124 244 301 324 328 628
TYW1 355
UBC 63
XIRP2 3003
WDR7 1444 1463
WDR41 377

Fig. S7. diGLY analysis of neurons stimulated with mitochondrial depolarisation

A. Ranking analysis of top mito Ub sites – MS1- and TMT-based intensity of all diGLY peptides was extracted and the top 25 diGLY sites with the largest relative abundance change upon 5h depolarization is indicated. Error bars represent SEM (n = 5). **B.** Ranking analysis of least abundant mito Ub sites – MS1- and TMT-based intensity of all diGLY peptides was extracted and the bottom 25 diGLY sites with the smallest relative abundance change upon 5h depolarization is indicated. Error bars represent SEM (n = 5). **C.** Diagram showing the sites of non MitoCarta 3.0 proteins in mouse cortical neurons. Residue numbers for diGLY modified Lys residues are shown.

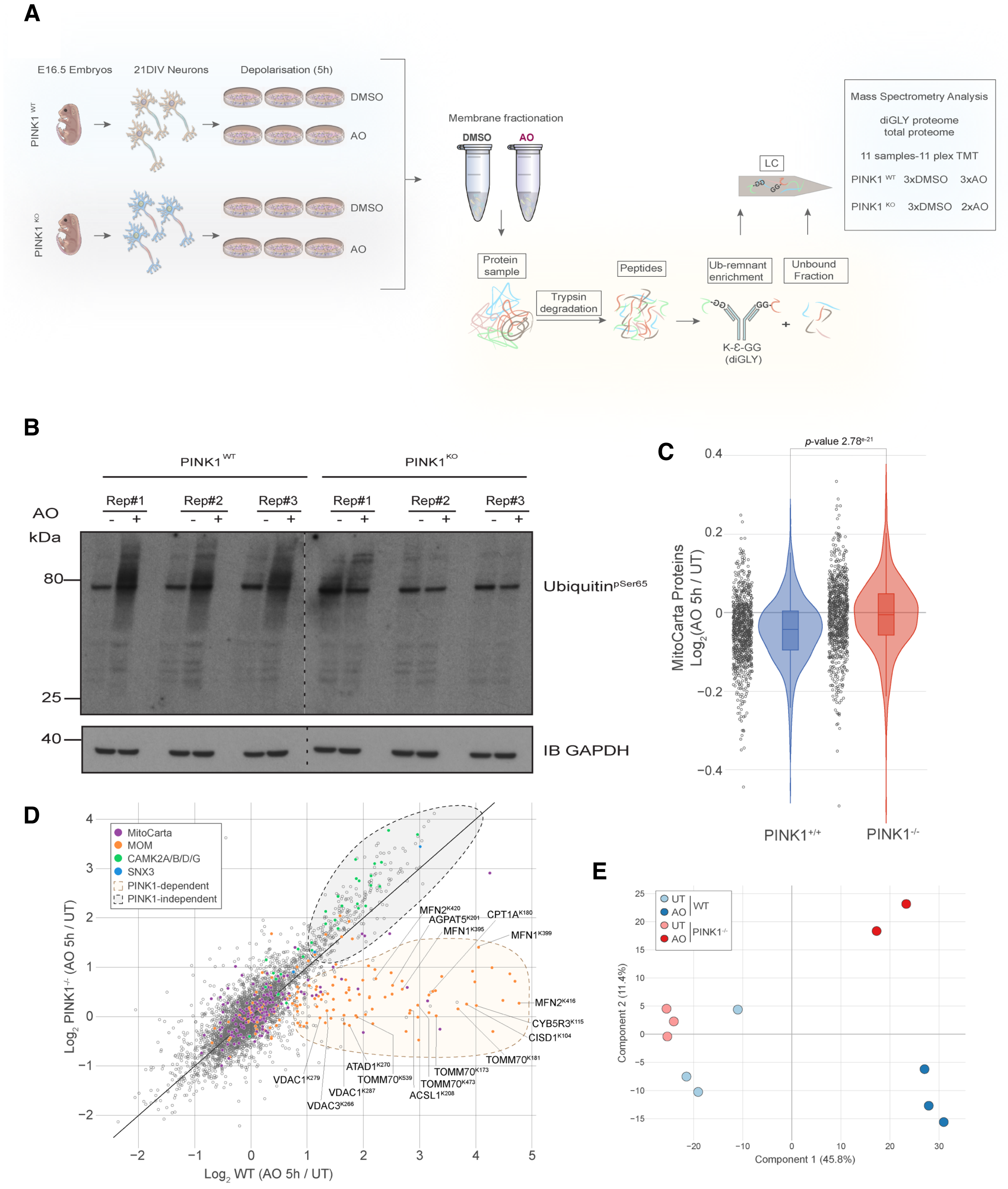
A
E16.5 Embryos
21DIV Neurons
Depolarisation (5h)
PINK1 WT
PINK1 KO
DMSO
AO
DMSO
AO
Membrane fractionation
DMSO
AO
Protein sample
Trypsin degradation
Peptides
Ub-remnant enrichment
Unbound Fraction
K-Ɛ-GG (diGLY)
LC
Mass Spectrometry Analysis
diGLY proteome
total proteome
11 samples-11 plex TMT
PINK1 WT 3xDMSO 3xAO
PINK1 KO 3xDMSO 2xAO
B
PINK1 WT
PINK1 KO
Rep#1
Rep#2
Rep#3
Rep#1
Rep#2
Rep#3
AO
- + - + - + - + - + - +
kDa
80
25
40
Ubiquitin pSer65
IB GAPDH
C
p-value 2.78e-21
MitoCarta Proteins
Log2(AO 5h / UT)
0.4
0.2
0
−0.2
−0.4
PINK1+/+
PINK1-/-
D
MitoCarta
MOM
CAMK2A/B/D/G
SNX3
PINK1-dependent
PINK1-independent
Log2 PINK1-/- (AO 5h / UT)
Log2 WT (AO 5h / UT)
MFN2K420
AGPAT5K201
MFN1K395
CPT1AK180
MFN1K399
MFN2K416
CYB5R3K115
CISD1K104
TOMM70K181
ATAD1K270
TOMM70K173
TOMM70K539
TOMM70K473
VDAC1K279
VDAC1K287
ACSL1K208
VDAC3K266
E
UT
AO
WT
UT
AO
PINK1-/-
Component 2 (11.4%)
Component 1 (45.8%)

Fig. S8. Validation of PINK1 activation in AO stimulated PINK1 knockout and wild-type cortical neurons.

A. Schematic representation of experimental setup in PINK1 knockout (KO) and wild-type primary cortical neurons. 3 biological replicates of E 16.5 mouse cortical neurons mouse were cultured for 21 days in vitro. Membrane enrichment performed after mitochondrial depolarisation induced with 10 μM of Antimycin A combined with 1 μM of Oligomycin for 5 h. **B.** 3 biological replicates of PINK1 WT and 3 biological replicates of PINK1 KO primary cortical neurons were stimulated with AO for 5h, DMSO as vehicle. Phospho-Ser65 ubiquitin was detected in membrane enriched lysates. GAPDH was used as a loading control. **C.** Violin plots for MitoCarta 3.0 proteins in wild-type and $PINK1^{-/-}$ primary cortical neurons. Relative protein abundance fold change after 5h depolarization is plotted for the MitoCarta 3.0 proteins quantified (black circles). Violin plots represent the distribution and density of the whole dataset (centre line, median; box limits correspond to the first and third quartiles; box whiskers, 1.5x interquartile range; violin limits, minimum and maximum values). Results (*p*-value) of a 2-tailed Mann-Whitney U-test comparing log_2(AO 5h/UT) between the $PINK1^{+/+}$ and $PINK1^{-/-}$ cell line is indicated. **D**. Correlation plots (data from Fig 3C&D) for the diGly sites quantified. diGLY-peptide of proteins associated with mitochondria (MitoCarta 3.0) or Mitochondrial outer membrane localization are indicated. Light coloured areas indicate PINK1-dependent or -independent sites. **E.** Principal component analysis of diGLY proteomics data obtained for the 11-plex experiment described in (A).

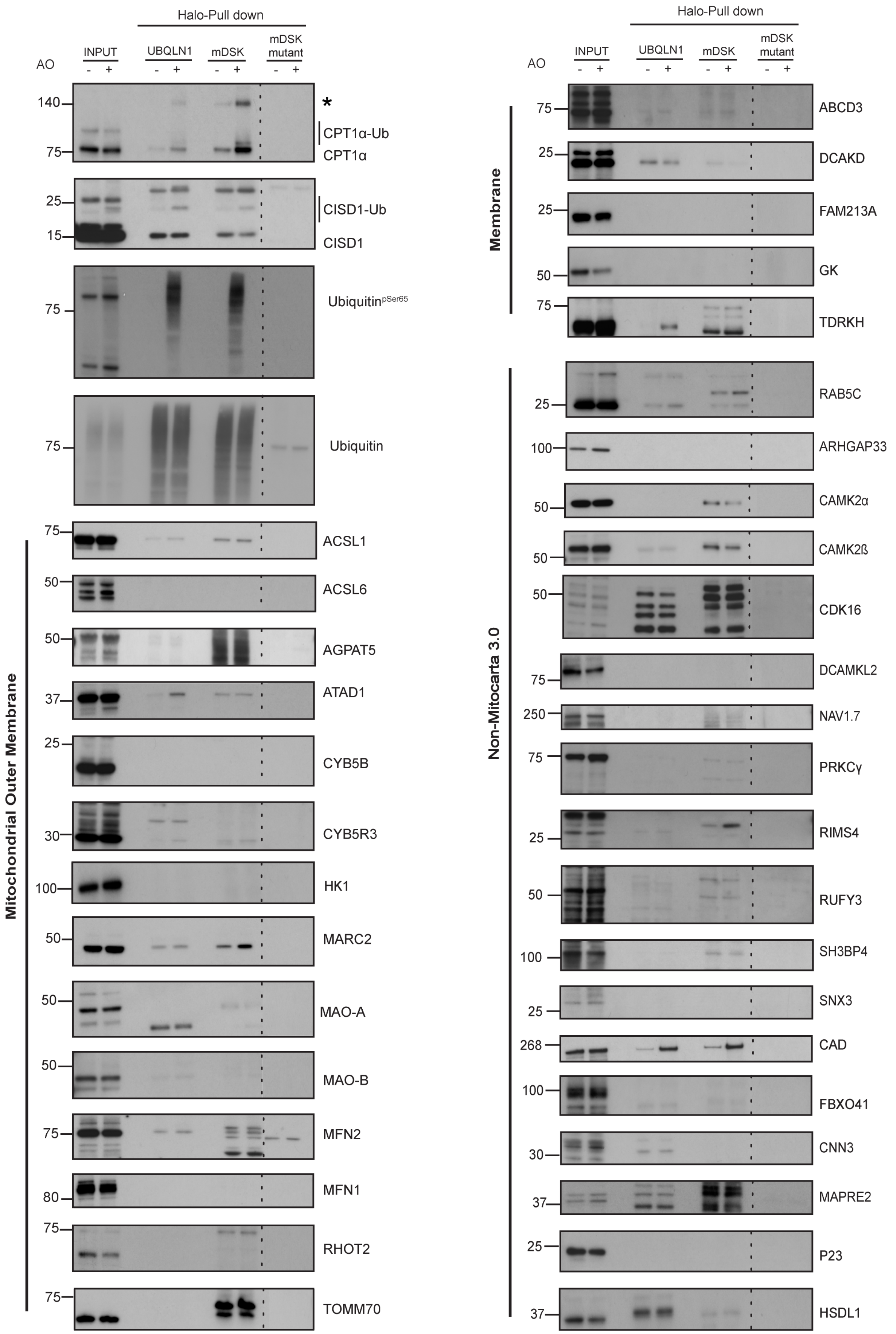

Halo-Pull down
AO
INPUT
UBQLN1
mDSK
mDSK mutant
- + - + - + - +
140
75
*
CPT1α-Ub
CPT1α
25
15
CISD1-Ub
CISD1
75
Ubiquitin pSer65
75
Ubiquitin
Mitochondrial Outer Membrane
75
ACSL1
50
ACSL6
50
AGPAT5
37
ATAD1
25
CYB5B
30
CYB5R3
100
HK1
50
MARC2
50
MAO-A
50
MAO-B
75
MFN2
80
MFN1
75
RHOT2
75
TOMM70
Membrane
75
ABCD3
25
DCAKD
25
FAM213A
50
GK
75
TDRKH
Non-Mitocarta 3.0
25
RAB5C
100
ARHGAP33
50
CAMK2α
50
CAMK2ß
50
CDK16
75
DCAMKL2
250
NAV1.7
75
PRKCγ
25
RIMS4
50
RUFY3
100
SH3BP4
25
SNX3
268
CAD
100
FBXO41
30
CNN3
37
MAPRE2
25
P23
37
HSDL1

Fig. S9. Biochemical analysis of ubiquitylated targets in AO stimulated C56BL/6J cortical neurons.

Membrane-enriched lysates from C57BL/6J cortical neurons, after 5 hours of AO stimulation, were subjected to ubiquitin capture using TUBE and multiDSK (mDSK) pull-down assay prior to immunoblotting with indicated antibodies. Ubiquitylated forms of CPT1α and CISD1 were detected after mitochondrial depolarisation. Anti-pSer65 Ubiquitin and Ubiquitin antibodies were used as controls. Targets were classified using MitoCarta 3.0

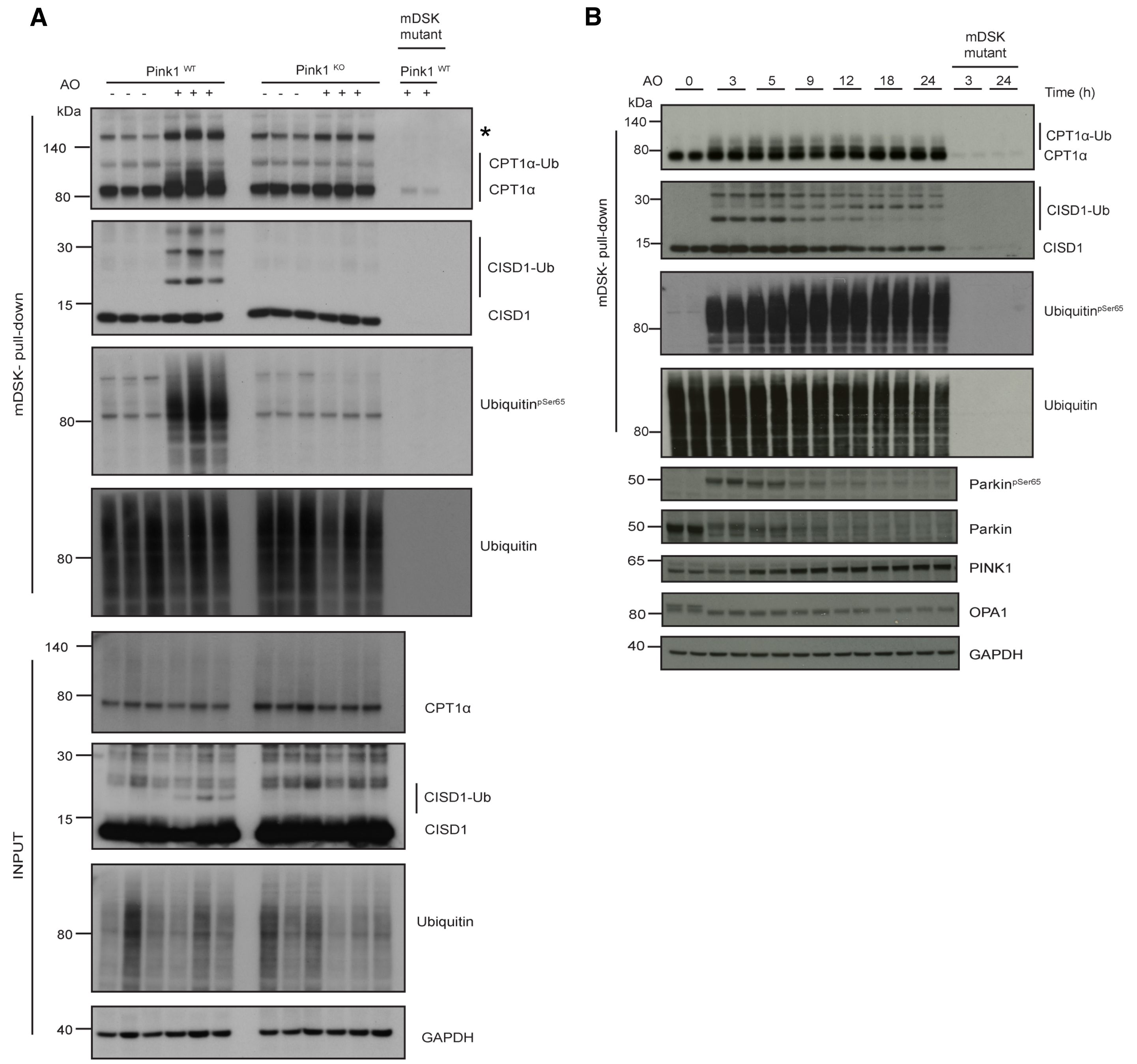
A
mDSK mutant
Pink1 WT
Pink1 KO
Pink1 WT
AO
kDa
140
80
*
CPT1α-Ub
CPT1α
30
15
CISD1-Ub
CISD1
80
Ubiquitin pSer65
80
Ubiquitin
mDSK- pull-down
140
80
CPT1α
30
15
CISD1-Ub
CISD1
80
Ubiquitin
40
GAPDH
INPUT
B
mDSK mutant
AO
0
3
5
9
12
18
24
3
24
Time (h)
kDa
140
80
CPT1α-Ub
CPT1α
30
15
CISD1-Ub
CISD1
80
Ubiquitin pSer65
80
Ubiquitin
mDSK- pull-down
50
Parkin pSer65
50
Parkin
65
PINK1
80
OPA1
40
GAPDH

Fig. S10. Analysis of CPT1α in PINK1 KO neurons and SH-SY5Y cells.

A. Membrane-enriched lysates of PINK1 WT and KO cortical neurons after 5 h of AO stimulation, were subjected to ubiquitylated-protein capture by Halo-multiDSK (mDSK), prior to immunoblot with anti-CPT1α, anti-CISD1, anti-phosphoSer65 Ubiquitin and anti-Ubiquitin antibodies. **B.** Time-course analysis of PINK1-Parkin pathway in SH-SY5Y cell lines. Ubiquitylated forms of CPT1α, CISD1 were found starting from 3 h of AO stimulation in SH-SY5Y cell lines, by using multiDSK ubiquitin pulldown assay. PhosphoSer65 Ubiquitin shows biochemical activation of PINK1.Total ubiquitin is used as loading control. Detection Parkin expression and Parkin Ser65 phosphorylation (Parkin^{pS65}) upon AO stimulation (3h, 5h, 9 h, 12h, 18h and 24h). PINK1 stabilisation and OPA1 cleavage are observed after mitochondrial depolarization. GAPDH is used as a loading control.

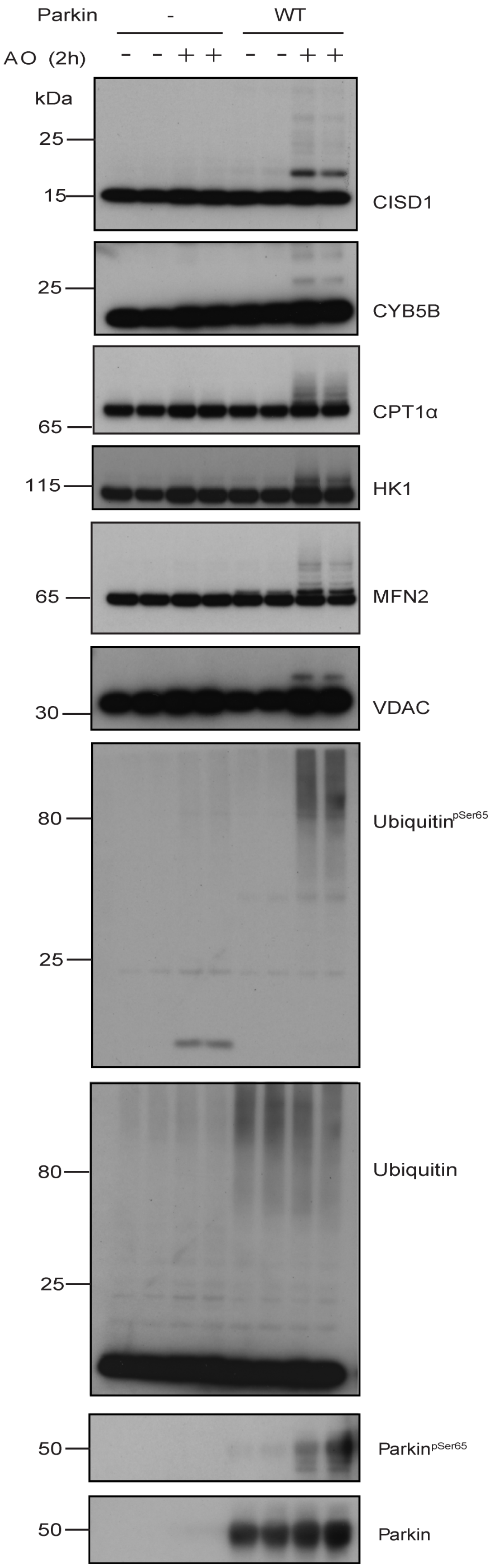
Parkin
-
WT
AO (2h)
- - + + - - + +
kDa
25
15
CISD1
25
CYB5B
CPT1α
65
115
HK1
65
MFN2
30
VDAC
80
25
Ubiquitin^pSer65
80
25
Ubiquitin
50
Parkin^pSer65
50
Parkin

Fig. S11. Validation of Parkin-dependent substrates in Hela cells.

HeLa cells were depolarized with AO for 2 hours, mitochondria were isolated and incubated with recombinant Parkin for *in vitro* ubiquitylation assay against endogenous mitochondrial substrates. Ubiquitylated proteins were detected with the indicated antibodies.

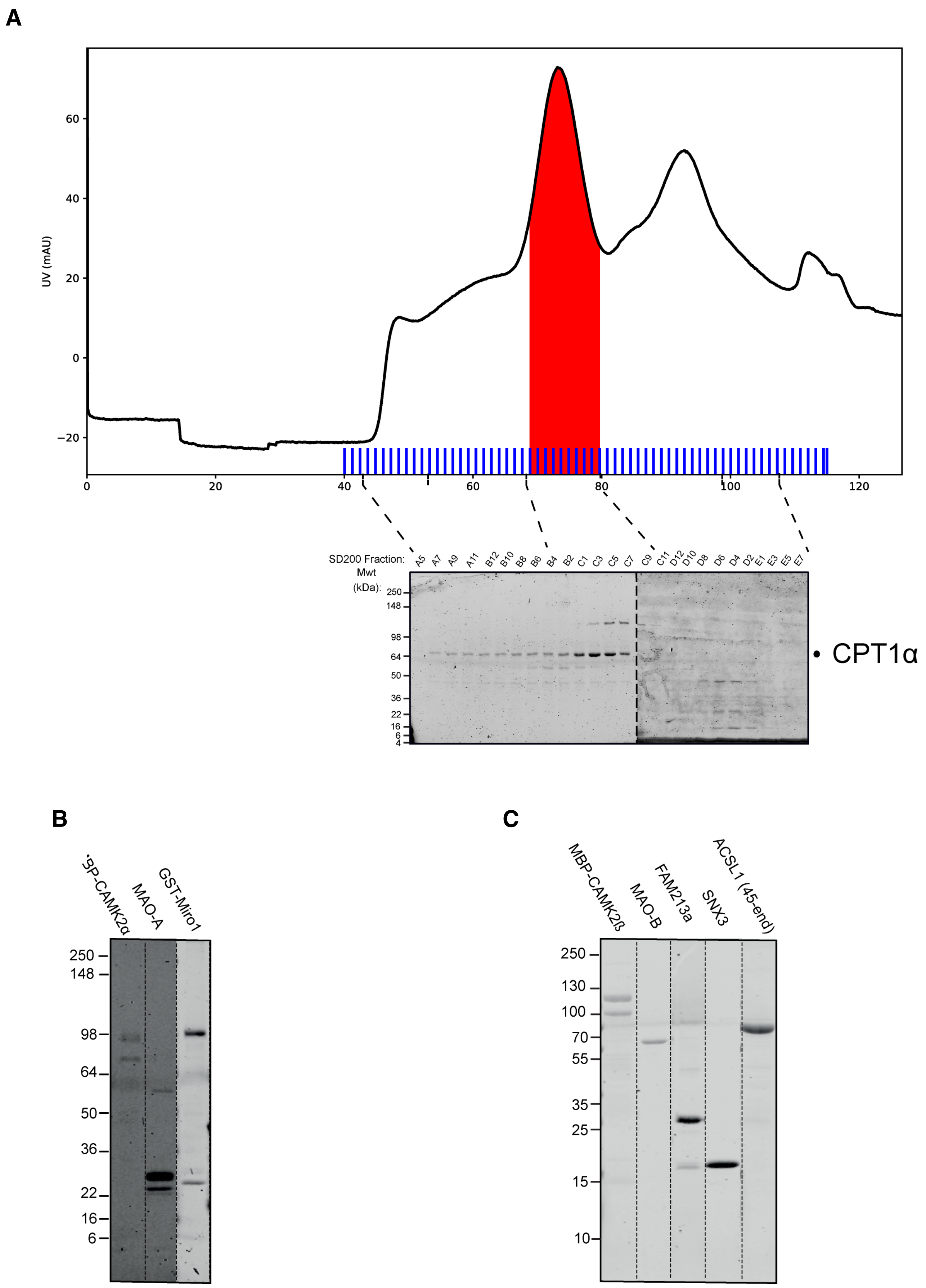

A
UV (mAU)
60
40
20
0
−20
0
20
40
60
80
100
120
SD200 Fraction:
Mwt
(kDa):
A5
A7
A9
A11
B12
B10
B8
B6
B4
B2
C1
C3
C5
C7
C9
C11
D12
D10
D8
D6
D4
D2
E1
E3
E5
E7
250
148
98
64
50
36
22
16
6
4
• CPT1α
B
SP-CAMK2α
MAO-A
GST-Miro1
250
148
98
64
50
36
22
16
6
C
MBP-CAMK2β
MAO-B
FAM213a
SNX3
ACSL1 (45-end)
250
130
100
70
55
35
25
15
10

Fig. S12. Purification of recombinant Parkin substrates and proteins.

A. 280 nm UV absorption trace for Cpt1α resolved on a Superdex 200 16/60 size exclusion column (black), with bounds of collected fractions in blue (top panel). Cpt1α fractions under UV absorption peaks resolved on a 4-20 % SDS-PAGE gel and stained using Coomassie brilliant blue (bottom panel). **B.** Purified parkin targets for MBP-CamK2α, MAO-A and GST Miro1; **C.** MBP-CamK2β, MAO-B, Fam213a, SNX3 and ACSL1 (45-end); in both cases 1μg of the final recombinant protein stock was resolved using 4-20 % SDS-PAGE and stained using Coomassie brilliant blue.

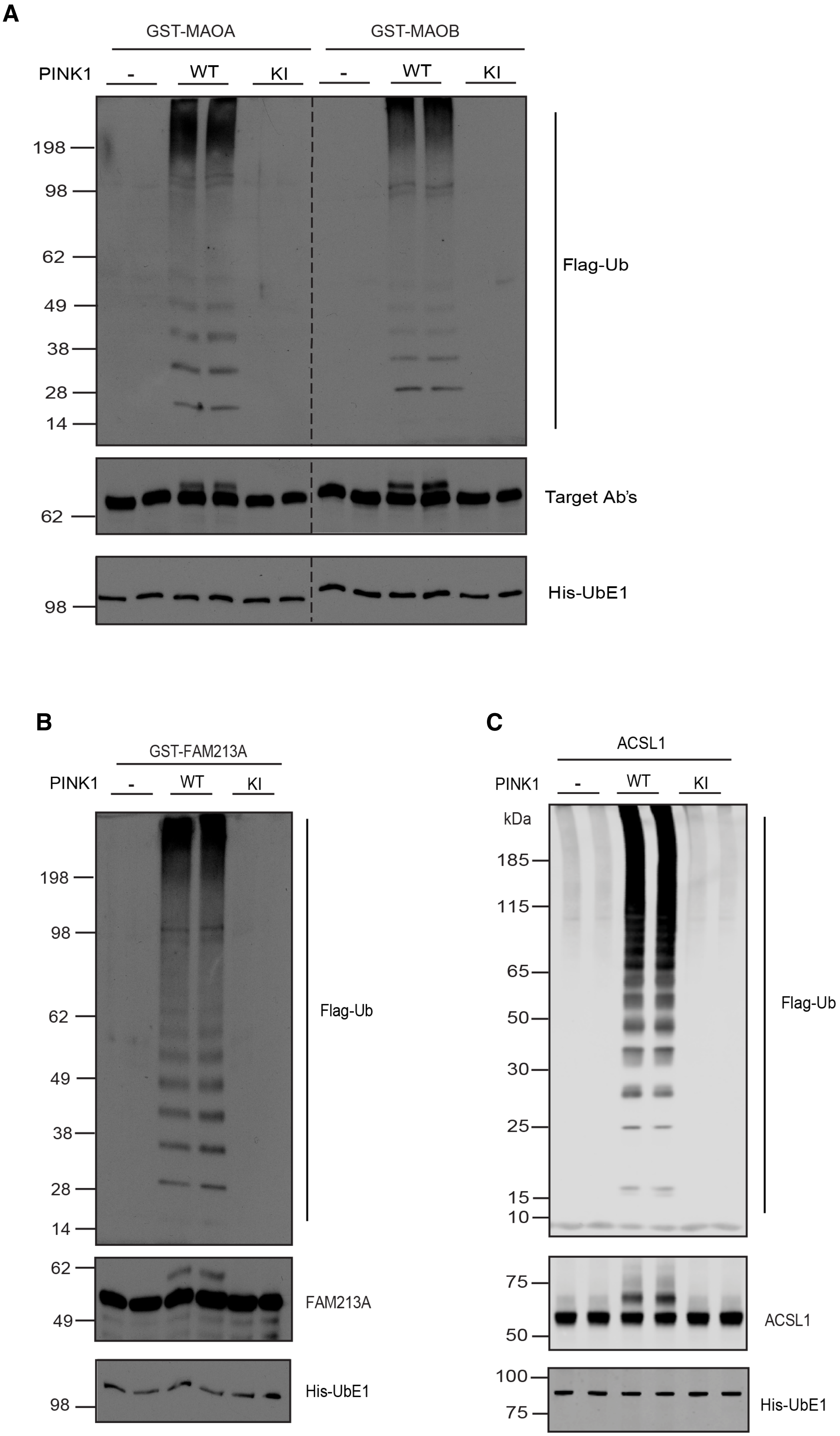
A
GST-MAOA
GST-MAOB
PINK1
-
WT
KI
198
98
62
49
38
28
14
Flag-Ub
Target Ab's
62
His-UbE1
98
B
GST-FAM213A
PINK1
-
WT
KI
198
98
62
49
38
28
14
Flag-Ub
62
FAM213A
49
His-UbE1
98
C
ACSL1
PINK1
-
WT
KI
kDa
185
115
65
50
30
25
15
10
Flag-Ub
75
ACSL1
50
100
His-UbE1
75

Fig. S13. *In vitro* Ubiquitylation assays of Parkin substrates.

A. Recombinant MAO-A and MAO-B proteins, **B.** FAM213A and **C.** ACSL1 were assessed for ubiquitylation by Parkin. For ubiquitylation assays Parkin was first activated using a 30 min kinase reaction at 37 °C in the presence or absence of wild type (WT) or kinase inactive (KI) TcPink1. Ubiquitylation reactions were started by the addition of the target protein, Flag-Ubiquitin, His-UbE1 and UbE2L3. Ubiquitylation reactions were stopped after a 30 minutes incubation at 37 °C unless stated otherwise by the addition of 4 x LDS loading buffer. Target proteins were resolved on a 4 to 12 % Bis-Tris gels, transferred to nitrocellulose membranes, and immunoblotted using anti-Flag, anti-His, and anti-targeted protein respectively. Membranes were imaged using LiCor.

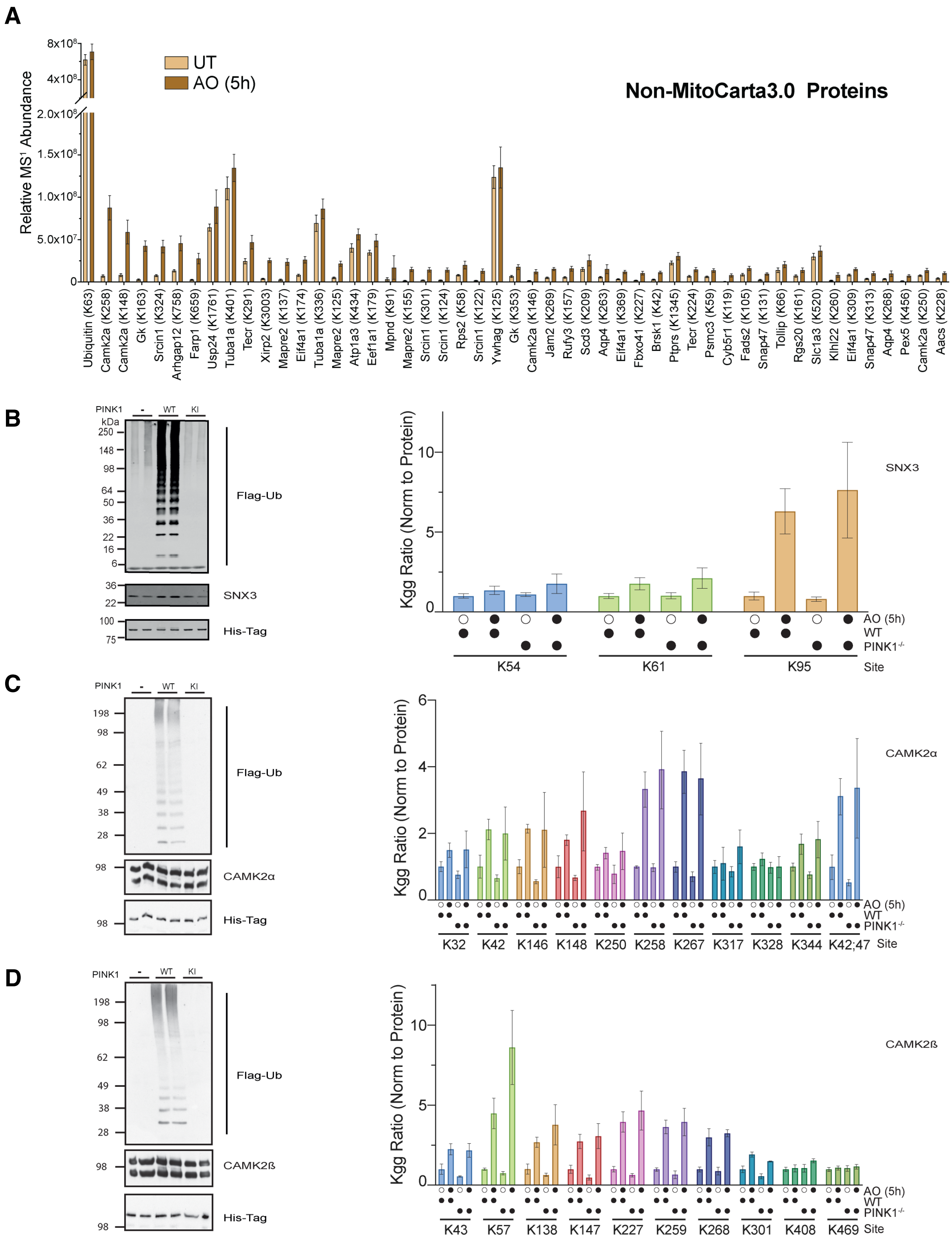
A
UT
AO (5h)
Non-MitoCarta3.0 Proteins
Relative MS¹ Abundance
8x10⁸
4x10⁸
2.0x10⁸
1.5x10⁸
1.0x10⁸
5.0x10⁷
0
Ubiquitin (K63)
Camk2a (K258)
Camk2a (K148)
Gk (K163)
Srcin1 (K324)
Arhgap12 (K758)
Farp1 (K659)
Usp24 (K1761)
Tuba1a (K401)
Tecr (K291)
Xirp2 (K3003)
Mapre2 (K137)
Eif4a1 (K174)
Tuba1a (K336)
Mapre2 (K125)
Atp1a3 (K434)
Eef1a1 (K179)
Mpnd (K91)
Mapre2 (K155)
Srcin1 (K301)
Srcin1 (K124)
Rps2 (K58)
Srcin1 (K122)
Ywhag (K125)
Gk (K353)
Camk2a (K146)
Jam2 (K269)
Rufy3 (K157)
Scd3 (K209)
Aqp4 (K263)
Eif4a1 (K369)
Fbxo41 (K227)
Brsk1 (K42)
Ptprs (K1345)
Tecr (K224)
Psmc3 (K59)
Cyb5r1 (K119)
Fads2 (K105)
Snap47 (K131)
Tollip (K66)
Rgs20 (K161)
Slc1a3 (K520)
Klhl22 (K260)
Eif4a1 (K309)
Snap47 (K313)
Aqp4 (K268)
Pex5 (K456)
Camk2a (K250)
Aacs (K228)
B
PINK1
-
WT
KI
kDa
250
148
98
64
50
36
22
16
6
Flag-Ub
36
22
SNX3
100
75
His-Tag
Kgg Ratio (Norm to Protein)
10
5
0
SNX3
AO (5h)
WT
PINK1⁻/⁻
K54
K61
K95
Site
C
PINK1
-
WT
KI
198
98
62
49
38
28
Flag-Ub
98
CAMK2α
98
His-Tag
Kgg Ratio (Norm to Protein)
6
4
2
0
CAMK2α
AO (5h)
WT
PINK1⁻/⁻
K32
K42
K146
K148
K250
K258
K267
K317
K328
K344
K42;47
Site
D
PINK1
-
WT
KI
198
98
62
49
38
28
Flag-Ub
98
CAMK2ß
98
His-Tag
Kgg Ratio (Norm to Protein)
10
5
0
CAMK2ß
AO (5h)
WT
PINK1⁻/⁻
K43
K57
K138
K147
K227
K259
K268
K301
K408
K469
Site

Fig. S14. PINK1 and Parkin independent ubiquitylation sites.

A. Ranking analysis of most abundant Ub sites not associated with MitoCarta 3.0 (data from Fig.2A) – MS1- and TMT-based intensity of all diGLY peptides was extracted and the top 50 diGLY sites with the largest relative abundance change upon 5h depolarization is indicated. Error bars represent SEM (n = 5). **B.** Recombinant SNX3 protein was assessed for ubiquitylation by Parkin and relative mass spectrometry analysis of ubiquitylation sites in wild-type and PINK1 knockout neurons. Kgg fold change to untreated wild-type cells (data from Fig S6A) is indicated. Error bars represent SEM, n = 3, 3, 3, 2. **C.** Recombinant CAMK2α protein was assessed for ubiquitylation by Parkin and relative mass spectrometry analysis of ubiquitylation sites in wild-type and PINK1 knockout neurons. Kgg fold change to untreated wild-type cells (data from Fig S6A) is indicated. Error bars represent SEM, n = 3, 3, 3, 2. **D.** Recombinant CAMK2β protein was assessed for ubiquitylation by Parkin and relative mass spectrometry analysis of ubiquitylation sites in wild-type and PINK1 knockout neurons. Kgg fold change to untreated wild-type cells (data from Fig S6A) is indicated. Error bars represent SEM, n = 3, 3, 3, 2.

A

ACSL1 207	VDKPEKAKLLLEGVE
AGPAT5 201	QRGLAVLKHVLTPRI
ATAD1 112	DTVILPIKKKHLFEN
ATAD1 270	KQREAILKLILKNEN
CPT1 180	RLPVPAVKDTVNRYL
CYB5R3 42	TLESPDIKYPLRLID
CYB5R3 115	LVIKVYFKDTHPKFP
CYB5R3 120	YFKDTHPKFPAGGKM
CYB5R3 154	GLLVYQGKGKFAIRP
CYB5R3 173	NPIIRTVKSVGMIAG
FKBP8 334	PILRAALKLEPSNKT
FKBP8 340	LKLEPSNKTIHAELS
GDAP1 207	DNVKYLKKILDELEK
GHTM 29	TKASPVVKNSITKNQ
MFN1 395	NLLTLDVKKKIKEVT
MFN1 399	LDVKKKIKEVTEEVA
MFN2 416	ELLAQDYKLRIKQIT
MFN2 420	QDYKLRIKQITEEVE
MFN2 720	EIAAMNKKIEVLDSL
RAB24 156	QLFETSSKTGQSVDE
RHOT1 187	EMKPACIKALTRIFK
RHOT2 409	EKRLDQEKGQTQRSV
SYNJ2BP 48	GIYVSRIKENGAAAL
TDRKH 112	NEIGAIEKAVIWPQY
TOMM70 170	FEQLQKWKEVAQDCT
TOMM70 178	EVAQDCTKAVELNPK
TOMM70 245	EKAKEKYKNREPLMP
TOMM70 536	RGLELISKAIEIDNK
VDAC1 266	LSALLDGKNVNAGGH
VDAC1 274	NVNAGGHKLGLGLEF
VDAC2 64	SSNTDTGKVTGTLET
VDAC2 285	SINAGGHKVGLALEL
VDAC3 53	HAYTDTGKASGNLET
VDAC3 266	LSALIDGKNFSAGGH
VDAC3 274	NFSAGGHKVGLGFEL

B

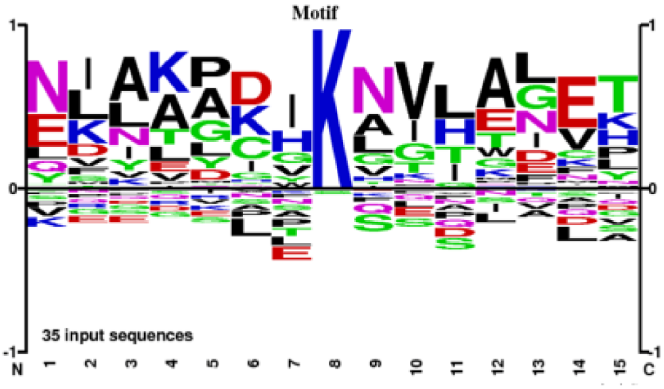

Fig. S15. Motif analysis for Parkin substrates.

A. Multiple sequence alignment analysis of human sequences (-7 residues to +7 residues of Lys) across common Parkin substrates identified. CISD1 Lys104 and TOMM70 Lys604 were excluded from analysis since Lys residues were located within 5 amino acids of C-terminus. **B.** Sequence Logo Analysis showed no putative targeting sequence for Parkin-directed Lysine ubiquitylation. Performed using PhosphoSitePlus v6.5.9.3 (https://www.phosphosite.org/sequenceLogoAction.action) online tool.

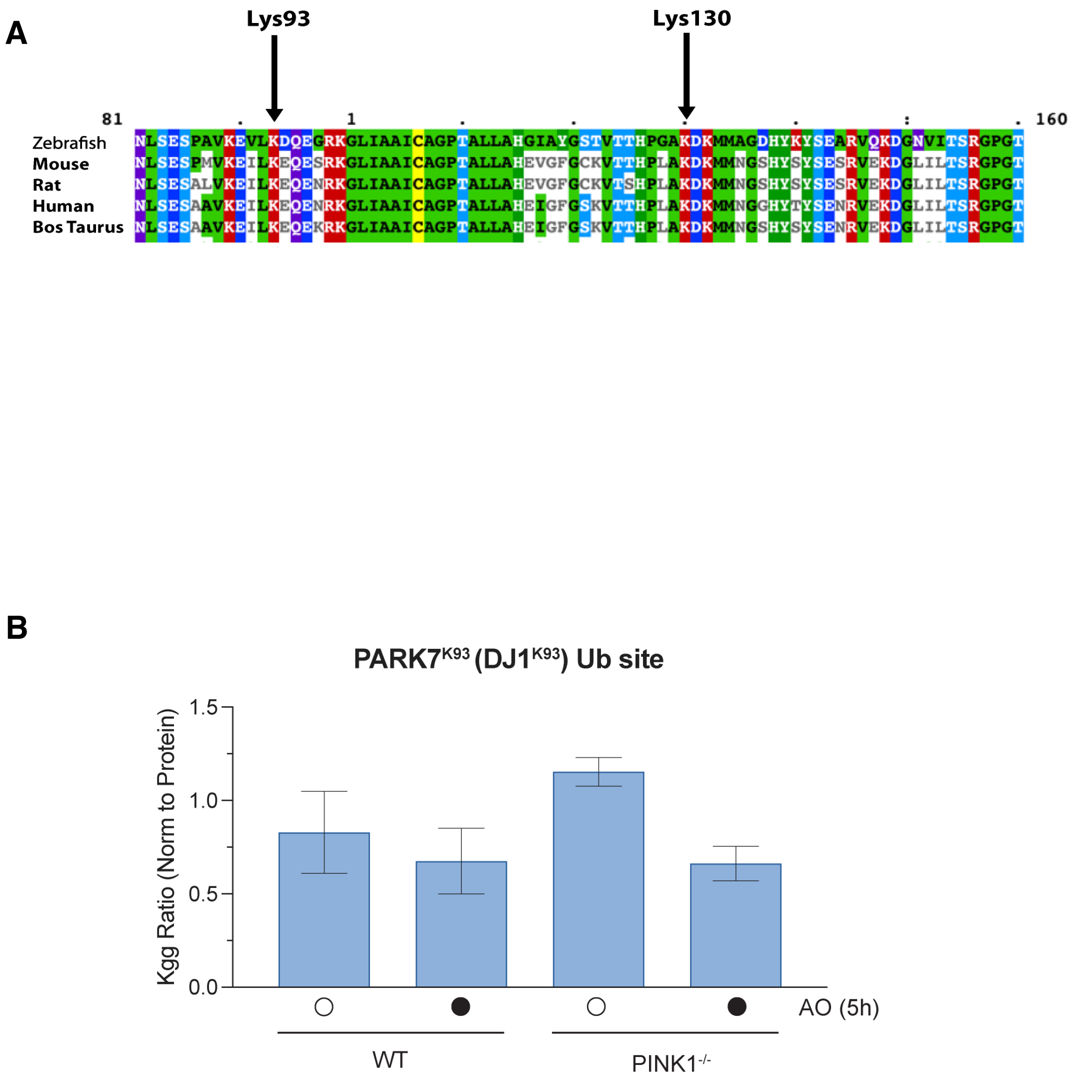

Fig. S16. DJ1 sites regulated by PINK1.

A. Multiple sequence alignment across species encompassing the ubiquitylation sites of DJ1 identified by mass spectrometry. **B.** Kgg fold change to untreated wild-type cells (data from FigS6A) is indicated for PARK7 lysine 93 ubiquitylation. Error bars represent SEM, n = 3, 3, 3, 2.

Table S1. Proteomic copy number analysis data in C57BL/6J Mouse Neurons.

Table S2. TMT total proteome and TMT diGLY peptide data in C57BL/6J Mouse Neurons under basal conditions and following mitochondrial depolarisation.

Table S3. Cytosolic ubiquitylated proteins elevated in Mouse Neurons following mitochondrial depolarisation.

Table S4. Kinases and Phosphatases ubiquitylated in Mouse Neurons following mitochondrial depolarization.

Table S5. TMT total proteome and TMT diGLY peptide data in PINK1 wild-type and knockout Mouse Neurons under basal conditions and following mitochondrial depolarization.
